# GALLO: An R package for genomic annotation and integration of multiple data sources in livestock for positional candidate loci

**DOI:** 10.1093/gigascience/giaa149

**Published:** 2020-12-30

**Authors:** Pablo A S Fonseca, Aroa Suárez-Vega, Gabriele Marras, Ángela Cánovas

**Affiliations:** University of Guelph, Department of Animal Biosciences, Centre for Genetic Improvement of Livestock, 50 Stone Rd E, Guelph N1G 2W1, ONT, Canada; University of Guelph, Department of Animal Biosciences, Centre for Genetic Improvement of Livestock, 50 Stone Rd E, Guelph N1G 2W1, ONT, Canada; University of Guelph, Department of Animal Biosciences, Centre for Genetic Improvement of Livestock, 50 Stone Rd E, Guelph N1G 2W1, ONT, Canada; The Semex Alliance, 5653 ON-6, Guelph N1G 3Z2, ONT, Canada; University of Guelph, Department of Animal Biosciences, Centre for Genetic Improvement of Livestock, 50 Stone Rd E, Guelph N1G 2W1, ONT, Canada

**Keywords:** multi-omics integration, QTL annotation, gene annotation, data mining, QTL enrichment analysis, livestock

## Abstract

**Background:**

The development of high-throughput sequencing and genotyping methodologies has enabled the identification of thousands of genomic regions associated with several complex traits. The integration of multiple sources of biological information is a crucial step required to better understand patterns regulating the development of these traits.

**Findings:**

Genomic Annotation in Livestock for positional candidate LOci (GALLO) is an R package developed for the accurate annotation of genes and quantitative trait loci (QTLs) located in regions identified in common genomic analyses performed in livestock, such as genome-wide association studies and transcriptomics using RNA sequencing. Moreover, GALLO allows the graphical visualization of gene and QTL annotation results, data comparison among different grouping factors (e.g., methods, breeds, tissues, statistical models, studies), and QTL enrichment in different livestock species such as cattle, pigs, sheep, and chickens.

**Conclusions:**

Consequently, GALLO is a useful package for annotation, identification of hidden patterns across datasets, and data mining previously reported associations, as well as the efficient examination of the genetic architecture of complex traits in livestock.

## Background

The identification of quantitative trait loci (QTLs), genomic regions linked to complex traits through association tests using genetic markers and phenotypic traits, is a crucial step in the improvement of genomic selection and economic profitability in livestock [1–4]. The development of high-throughput methodologies (e.g., genome-wide association studies [GWAS], transcriptomics, metabolomics, proteomics) for the study of the genetic architecture of complex traits allows for the identification of potential candidate genes associated with economically relevant traits in livestock. Taken together, these technologies can substantially improve the accuracy of detection of candidate regions associated with economically important traits across the genome in livestock species [5]. Consequently, the number of QTLs identified across the genome in livestock species has increased substantially in the past few years. As of October 2020, the Animal Quantitative Trait Loci Database (Animal QTLdb) can retrieve information about QTLs previously identified in cattle (159,844), chickens (12,508), horses (2,451), pigs (30,871), rainbow trout (584), and sheep (3,411) [6]. The proper integration of results obtained from different methodologies and technologies is a crucial step for the accurate identification of the biological processes regulating complex traits, as well as the identification of potential functional candidate genes for each trait or those shared among traits [5, 7–9]. The integration of both structural and functional data can help scrutinize the genetic architecture of economically relevant traits and, consequently, help to better elucidate complex biological patterns regulating the expression of these traits, such as pleiotropic effects, epistasis, and genetic hitchhiking, among others.

Despite the potential to improve the identification of functional candidate genes and/or QTLs through the integration of multiple data sources, the current process poses limitations in the pipelines and algorithms implemented in the tools available for livestock. Currently, there are several tools that implement functions for gene (i.e., Biomart and BEDTools) and QTL annotation (Animal QTLdb) [6, 10, 11]. However, these tools have limitations regarding the automatization process to analyze results from multiple candidate regions (Biomart web application and the R package and Animal QTLdb) or for the visualization of the results. Moreover, although automatization is possible, there is no direct link between the candidate regions and/or markers with the annotated genes and QTLs. Consequently, this gap forces the user to back-solve the overlap between the input and output files in order to perform the proper association between the candidate region and/or markers and the annotated genes and/or positional co-localized QTLs. In addition, there is still a need for customized QTL enrichment analyses in the available software and databases. Genomic Annotation in Livestock for positional candidate LOci (GALLO) is an R package designed to provide an automatized and a straightforward environment for gene and QTL annotation in multiple candidate regions, as well as the integration of data from multiple sources. Additionally, the QTL enrichment analysis can be performed directly by GALLO using the output obtained from the QTL annotation step. GALLO also provides a set of functions for graphical visualization of the annotation, comparison, integration, and QTL enrichment results. In this context, the GALLO package was developed as an alternative tool (i) to allow the integration and simultaneous annotation of multiple datasets for genes and QTLs, (ii) to provide graphical visualization tools to visually integrate the annotation and similarity against datasets, and (iii) to perform QTL enrichment analysis for the positional candidate genomic regions and/or markers associated with economically relevant traits in livestock.

### Implementation

The GALLO package was written in the R language [12]. The stable release is available as an R package on CRAN [13]. The code was extensively tested with several datasets from different sources and methodologies and reviewed to ensure that it meets the package's high quality standards. Additionally, the vignettes were created to be comprehensive and to present practical examples to provide a user-friendly tutorial.

**Figure 1:**
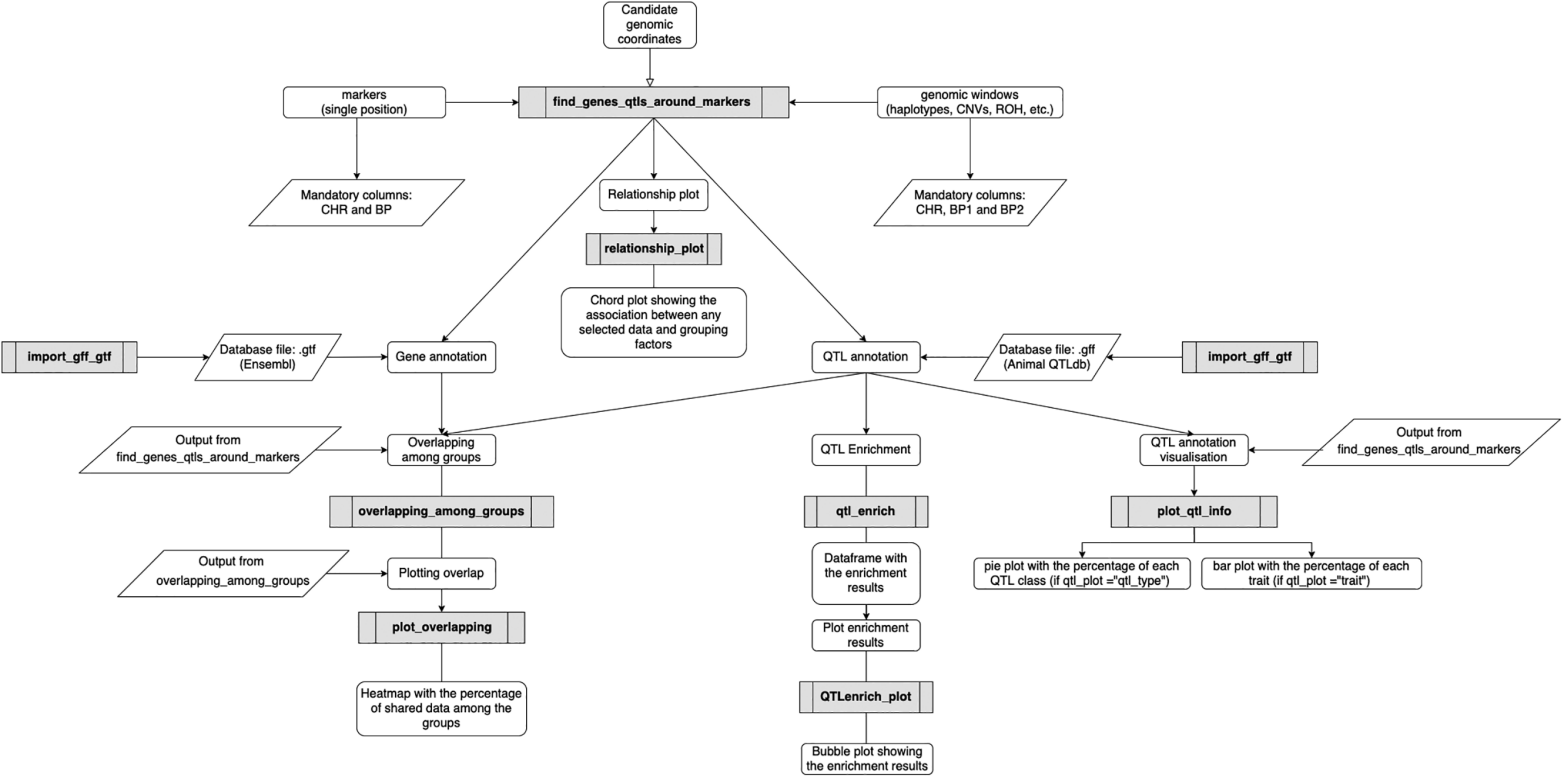
Workflow explaining the main functions implemented on GALLO. The grey rectangles represent the functions, while the rounded, sharp rectangles and parallelograms represent the main goal of that respective function, its input and outputs, respectively.

The GALLO package provides a useful set of functions that gives a straightforward approach to data integration, comparison, gene and QTL annotation, and visualization of several data sources and methodologies, such as variants from GWAS, RNA sequencing, whole-genome sequencing, and so forth (Fig. 1 and Table 1). The main advantage to performing an automated analysis from multiple datasets is the ability to handle the output using different subsets (e.g., traits, populations, models) in the same environment without generating multiple intermediate output files.

**Figure 4:**
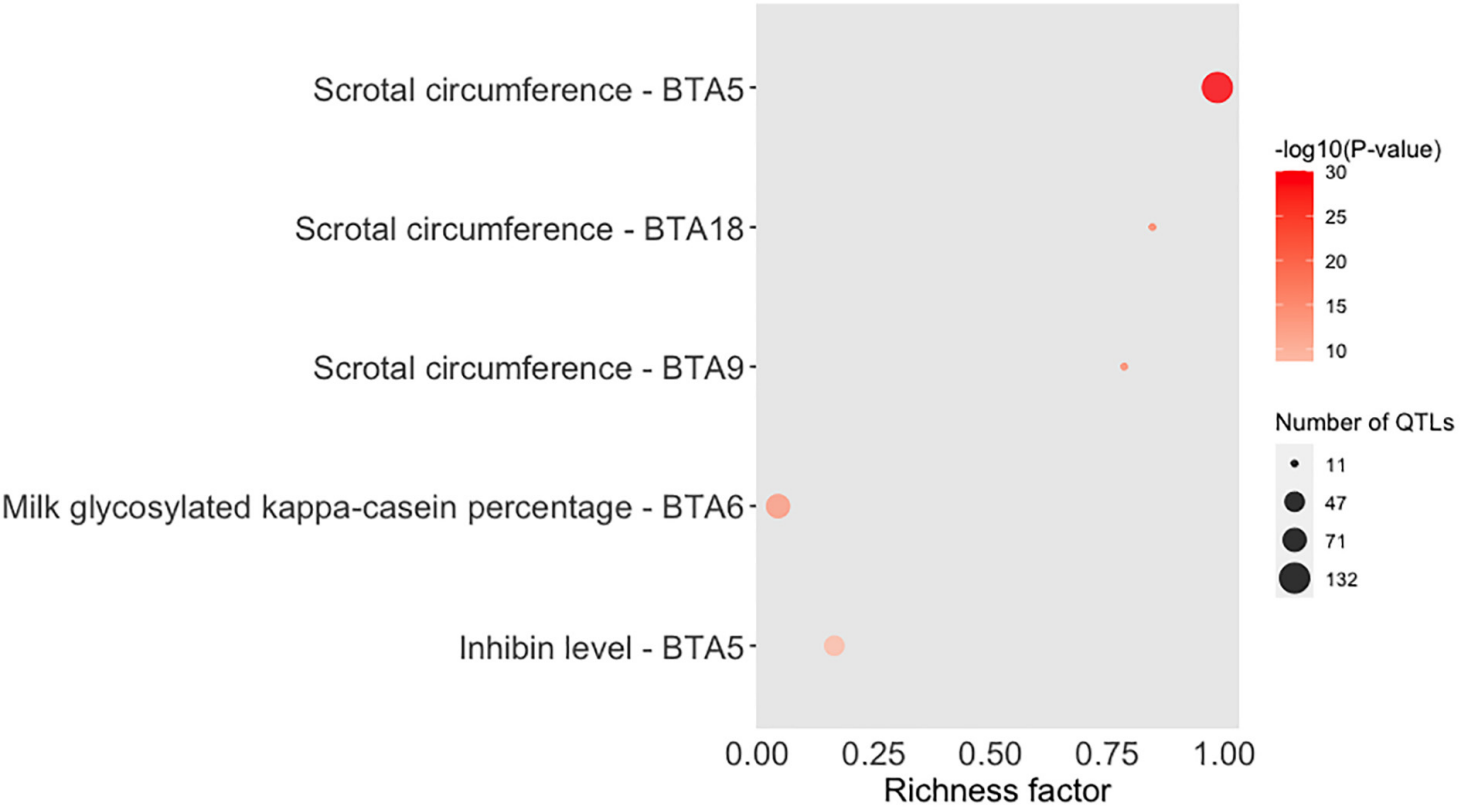
Bubble plot displaying the enrichment results for the top 5 enriched QTLs identified using the QTLs annotated within the candidate regions from Feugang et al. [15] and Buzanskas et al. [14]. The darker the red shade in the circles, the more significant the enrichment. The area of the circles is proportional to the number of QTLs. The x-axis shows a richness factor obtained by the ratio of the number of QTLs annotated in the candidate regions and the total number of each QTL (and chromosome in the case of this plot) in the reference database.

**Table 1:** Description of the functions implemented in the GALLO package

Function	Description	Output
**Gene and QTL annotation**		
import_gff_gtf	Import the gff and gtf files used for QTL and gene annotation, respectively	A dataframe composed by the information present in the gtf and gff files
find_genes_qtls_around_markers	Annotation of genes and QTLs around candidate regions	A dataframe composed of the columns present in the input file and the genes or QTLs mapped within or around (if interval provided) the candidate regions
**Data visualization**		
overlapping_among_groups	Overlap between grouping factors (e.g., different traits, statistical models, populations, studies)	A list with 3 matrices: (i) a matrix with the number of overlapping data, (ii) a matrix with the percentage of overlap, and (iii) a matrix with the combination of (i) and (ii)
plot_overlapping	Plot overlap between data and grouping factors	A heat map with the overlap between groups
plot_qtl_info	Plot QTL information from the gene or QTL annotation output	A pie chart (if QTL class is chosen) or a bar plot (if trait name is chosen) for the annotated QTLs
relationship_plot	Plot the relationship among the candidate regions or grouping factors with the annotated genes and QTLs	A chord plot linking a grouping factor (e.g., genomic regions, traits, populations) with the annotated genes or QTLs
**QTL enrichment**		
qtl_enrich	Performs a QTL enrichment analysis based on a bootstrap simulation for each QTL class or trait	A dataframe composed of the enrichment results for QTL classes or traits present in the input file: (i) QTL: the QTL class or trait used for the enrichment; (ii) CHR: the chromosome for that specific QTL or trait (if the option “chromosome” is informed to the argument enrich_type); (iii) N_QTLs: number of observed QTLs or traits in the dataset; (iv) N_QTLs_db: number of each annotated QTL in the qTL database; (v) Total_annotated_QTLs: total number of annotated QTLs; (vi) Total_QTLs_db: total number of QTLs in the QTL database; (vii) pvalue: *P*-value for the enrichment analysis; (viii) adj.pval: the adjusted *P*-value based on the multiple test correction selected by the user; (ix) QTL_type: the QTL type for each annotated trait
QTLenrich_plot	Creates a bubble plot with the QTL enrichment results	A plot with the QTL enrichment results

### Case study—Candidate regions for scrotal circumference and fertility in cattle

The dataset used to present the basic usage and advantages of the GALLO package is composed by the markers significantly associated with scrotal circumference in the Canchim breed [14] and noncompensatory fertility in Holstein cattle [15]. These 2 studies were previously analyzed together in a systematic review regarding male fertility in cattle [8]. Therefore, the data used herein comprise a multi-study and multi-breed analysis. These candidate markers (527 single-nucleotide polymorphisms [SNPs]) are available in Supplementary Table S1. In addition to the candidate markers, we present, as Supplementary Files S1 and S2, the annotation gff file containing the QTL database information for cattle (obtained from the Animal QTLdb) [16] and the gtf file containing the genes annotated in the cattle genome obtained from Ensembl [17]. The genomic coordinates of both files were based on the bovine reference genome version UMD 3.1 due to the original coordinates used to report the location of the candidate markers in the original studies. Here, the analysis performed follows the same logical order to the one presented in the GALLO vignette [18]. However, the dataset used in the user practical tutorial is a subset of the data presented here, aiming to reduce the computational demand for the user. The script with all the commands used to perform the analysis presented here is available in Supplementary File S3. All the tests were performed using a desktop with an Intel Core i5 2.4 GHz processor with 8 GB of RAM.

### Importing datasets and annotating genes and QTLs around candidate markers

The first step in the pipeline consists of importing the databases that will be used for the analysis with the import_gff_gtf() function. In our specific example, we imported both cattle gene annotation (gtf) and QTL (gff) databases. The import_gff_gtf() function receives the database file (db_file) and the file type (file_type = “gff” or “gtf”) as arguments and creates a dataframe with the respective information from each file. The system time taken to import the gtf and gff files was 0.045 and 0.311 seconds, respectively, indicating an efficient importing process. The file containing the candidate markers can be imported using any available function in the R environment such as read.table() and read.csv().

The main function of GALLO, find_genes_qtls_around_markers(), performs the annotation of genes and/or co-localized QTLs within or nearby candidate markers or genomic regions (using the user's defined interval/window). This function uses the information provided in the .gtf file (for gene annotation) or .gff (for QTL annotation) to retrieve the requested information. The output combines the information available in the input file provided by the user with the information available for the genes and QTLs mapped in the candidate genomic regions. For example, for an input file composed of 3 genomic coordinates where 4 genes are annotated in each of the intervals determined by the user, the output file of find_genes_qtls_around_markers() will contain 12 rows. The minimum information necessary for the gene and QTL annotation procedures is a dataframe with 2 columns containing the chromosome (CHR) and position in base pairs (BP) in the case of the candidate SNPs input file. In the case of the candidate haplotypes, windows, copy number variations (CNVs), or candidate regions, the input file is composed by 3 columns corresponding to the chromosome (CHR), the start position in base pairs (BP1), and the end position in base pairs (BP2). Data examples for the candidate markers and windows input files can be obtained using the data(“QTLmarkers”) and data(“QTLwindows”) commands in R. Additionally, examples of QTL and gene annotation results are accessible through the data(“gtfGenes”) and data(“gffQTLs”) commands, respectively. These outputs can be easily handled by summary functions in R, such as table(), to obtain information such as the total number of genes and QTLs and the number of genes and QTLs annotated per variant. The gene annotation process was compared with the getBM() function from the biomaRt package. The gene annotation process on GALLO needed 0.424 seconds to completely annotate the genes in a 200-kb interval (upstream and downstream) from candidate markers, while the biomaRt function required 0.019 seconds. The QTL annotation on GALLO was compared with the Bedtools -wao -C command, resulting in 0.851 and 0.12 seconds required for each approach, respectively. It is important to highlight that for both gene and QTL annotation using biomaRt and Bedtools, respectively, a posterior processing of the output file is required in order to match the candidate markers and the genes and QTLs mapped within the candidate intervals. On the other hand, the output file from the find_genes_qtls_around_markers() function was designed to allow this match in an intuitive way, combining the rows of both candidate marker filse and database files (gff and gtf). Additionally, GALLO allows the user to perform both annotations for genes and QTLs with a single software package and programming language. Consequently, GALLO obtains a more elaborate and informative output without substantially compromising the computational demand required for the analysis. The output files obtained in the gene and QTL annotation are available in Supplementary Tables S2 and S3, respectively.

### Comparing and visualizing the overlapping of genes and QTLs annotated within the candidate regions

**Figure 2:**
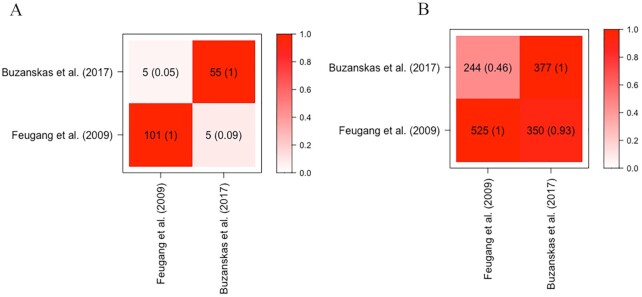
Overlapping between genes (A) and QTLs (B) annotated within the candidate regions (100 kb downstream and upstream from the significant markers) from Feugang et al. and Buzanskas et al. [14, 15]. The darker the color within the squares, the higher the percentage of shared genes or QTLs.

The output file generated by the find_genes_qtls_around_markers() function can be used as an input file for the other set of GALLO functions. An advantage from the output of the find_genes_qtls_around_markers() function is that any additional information present in the input file will be retained in the output file. Consequently, this information can be used to compare the retrieved information between groups of population, methodologies, statistical models, and so forth. For example, the functions overlapping_among_groups() and plot_overlapping() can be used to create matrices with the overlapping values among groups and to visualize this overlap. Figure 2 shows the genes and QTLs overlapping between the positional markers obtained in the 2 selected studies from the dataset of markers analyzed, Feugang et al. [15] and Buzanskas et al. [14]. It is important to highlight that the overlapping matrix informing the percentage of shared records is not symmetrical. The percentages of genes from Study A shared with Study B, and vice versa, are calculated as a function of the total number of genes in A or B, respectively. Briefly, this matrix is not symmetrical because GALLO calculates the percentage of records shared as a function of the total number of records for each group. For example, Groups A and B shared 5 records, where Group A has 10 records in total and Group B has 5 records. Consequently, the percentage of shared records in A is 50% while the percentage of shared records in B is 100%. In the present example, it can be noted that only a small percentage of the positional candidate genes were shared between the studies. However, the analyses of overlapping QTLs (using the trait name as reference ID) indicated a higher similarity between the studies: 46% of the QTLs annotated in the candidate regions from Feugang et al. [15] were also present in Buzanskas et al. [14] and 93% of the QTLs annotated in the candidate regions from Buzanskas et al. were also present in Feugang et al.

### Understanding the QTL context of the candidate regions

**Figure 3:**
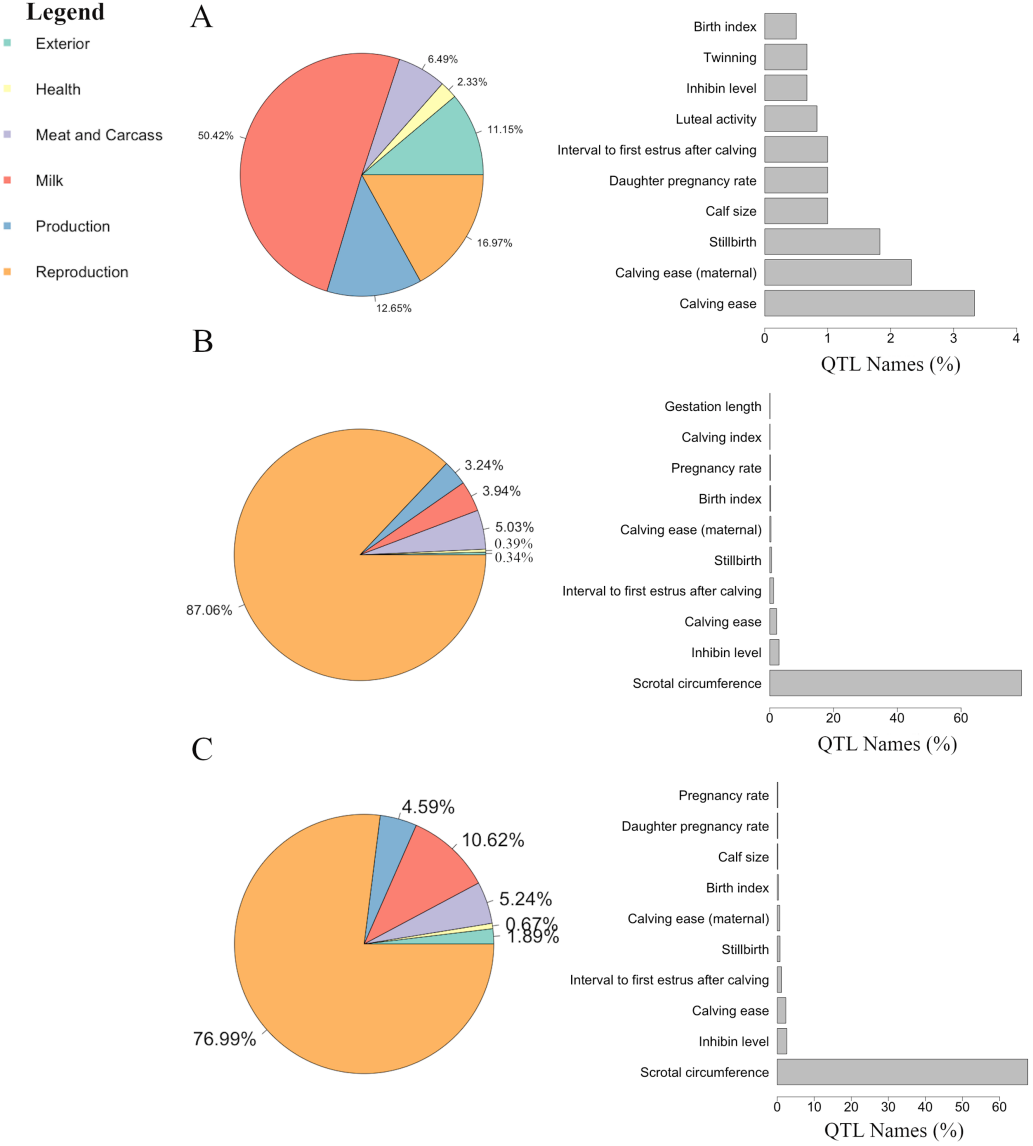
Percentage of QTL type (pie chart) and trait related to peproduction QTLs (barplots) for the QTL annotation results obtained for (A) Feugang et al. [15], (B) Buzanskas et al. [14], and (C) the combined analysis (using both studies).

A more precise investigation of the QTL representativeness and diversity can help to better elucidate the genomic context of the candidate regions. The recurrent association of particular genomic regions with multiple traits might suggest the presence of complex genetic mechanisms regulating that region, such as pleiotropy, epistasis, or hitchhiking effect, among others [19, 20]. The plot_qtl_info() function from GALLO allows for the graphical visualization of the summary of QTL types and traits annotated. The percentage of each QTL type for cattle (i.e., milk, meat and carcass, health, production, reproduction, and exterior) annotated within the candidate regions is presented in a pie chart through the use of the argument qtl_plot = "qtl_type," while the percentage of each trait associated with a specific QTL type can be plotted using the argument qtl_plot = "qtl_name" and informing the additional argument qtl_class (that must receive the name of the QTL class to be plotted). Fig. 3 shows that for Feugang et al. [15] the 2 most frequent QTL types were milk (50.42%) and reproduction (16.97%), while for Buzanskas et al. [14] the most frequent QTL types were reproduction (87.06%) and meat and carcass (5.03%). An in-depth analyses can be performed for each QTL type in order to observe the frequency of each trait associated with a specific QTL type. The most frequent traits related to reproduction QTLs were calving ease (>3%) and scrotal circumference (>60%) for Feugang et al. and Buzanskas et al. [14, 15], respectively (Fig. 3). The comparison between the frequency of traits related to reproduction QTLs annotated in Feugang et al. and Buzanskas et al. [14, 15] indicated that among the top 10 most frequent QTLs, calving ease, inhibin levels, stillbirth, interval to first estrus after calving, and birth index were shared between the studies. The combined analysis (not filtering by study) indicated that the reproduction and milk QTL types were the 2 most frequent classes, with 76.99% and 10.62% of all QTL types, respectively. In addition, scrotal circumference, inhibin level, and calving ease were the most frequent reproduction QTL–related traits in the combined analysis.

### QTL enrichment analysis

In some cases, the biases produced with more research in certain areas/traits of higher relevance to animal production (such as milk production–related traits in the QTL database for cattle) may result in a larger proportion of records for these traits in the QTL database. Consequently, the simple investigation of the proportion of each QTL type might not be totally useful. The GALLO package allows the user to perform a QTL enrichment analysis to test the significance of the QTL representativeness. The QTL enrichment analysis function in the GALLO package is based on a hypergeometric test approach, where the number of QTLs annotated within the candidate regions for each QTL type or trait is compared with the observed number of QTLs in the reference database. Briefly, using an enrichment for individual traits in a chromosome-wide approach as an example, the number of traits per chromosome annotated within the candidate regions and the total number of each individual trait in the QTL database are computed. Subsequently, this information is integrated into a hypergeometric test to estimate whether the number of observed records, for a specific trait, in a chromosome is larger than expected by chance. The qtl_enrich() function allows the user to perform the QTL enrichment analysis for both QTL types and traits (qtl_type = “QTL_type” or “Name”), for the whole genome or chromosome-wide (enrich_type = “genome” or “chromosome”), and for all the annotated chromosomes or a subset (chr.subset = NULL or the object with the subset of chromosomes). The use of a chromosome-wide enrichment analysis might help to detect specific regions across the genome with a high number of QTLs for a specific trait, e.g., BTA14 in cattle for milk production [21]. A total of 161 unique pairs of traits and chromosomes were tested for the enrichment using the annotated QTLs from both studies. The system time required to perform the enrichment analysis was 5.32 seconds, suggesting efficient processing. The top 10 enriched QTLs (false discovery rate [FDR] < 0.05) for the combined analysis are reported in Table 2 and the enrichment results for all the annotated QTLs are presented in Supplementary Table S4. Additionally, GALLO also allows the user to obtain a graphical visualization, in a bubble plot, of the enrichment results using the QTLenrich_plot() function. This function receives the enriched table obtained from qtl_enrich(), the name of the column with the trait names to be plotted, and the name of the column with the *P*-values to be plotted as arguments. A total of 28 pairs of traits and chromosomes were found to be enriched in the combined analysis, with scrotal circumference (BTA 5, 18, 9, and 21), milk glycosylated κ-casein percentage (BTA 6 and 16), inhibin level (BTA 5), triglyceride level (BTA 5), milk κ-casein percentage (BTA 6), and milk iron content (BTA 23) in the list of top 10 most enriched traits. Fig. 4 shows the top 5 enriched QTLs identified in this analysis.

**Table 2:** Top 10 enriched QTLs for the combined analysis performed with the candidate regions from the 2 studies, Feugang et al. [15] and Buzanskas et al. [14], used in the example dataset

QTL	CHR	No. QTLs	No. QTLs db	Total No. QTLs	Total No. QTLs db	*P*-value	FDR	QTL type
Scrotal circumference	5	132	134	347	5,942	1.56E−171	4.98E−169	Reproduction
Scrotal circumference	18	11	13	41	2,147	2.20E−18	3.52E−16	Reproduction
Scrotal circumference	9	11	14	30	1,395	2.04E−17	2.18E−15	Reproduction
Milk glycosylated κ-casein percentage	6	71	1,607	204	12,158	1.86E−15	1.49E−13	Milk
Inhibin level	5	47	285	347	5,942	3.38E−11	2.16E−09	Reproduction
Scrotal circumference	21	4	5	12	3,606	3.51E−10	1.87E−08	Reproduction
Milk κ-casein percentage	6	76	2,637	204	12,158	2.39E−07	1.01E−05	Milk
Triglyceride level	5	6	7	347	5,942	2.53E−07	1.01E−05	Health
Milk glycosylated κ-casein percentage	16	7	44	21	1,440	1.29E−06	4.58E−05	Milk
Milk iron content	23	4	8	19	1,159	3.48E−06	0.000111329	Milk

### Relationship between studies and enriched QTLs

**Figure 5:**
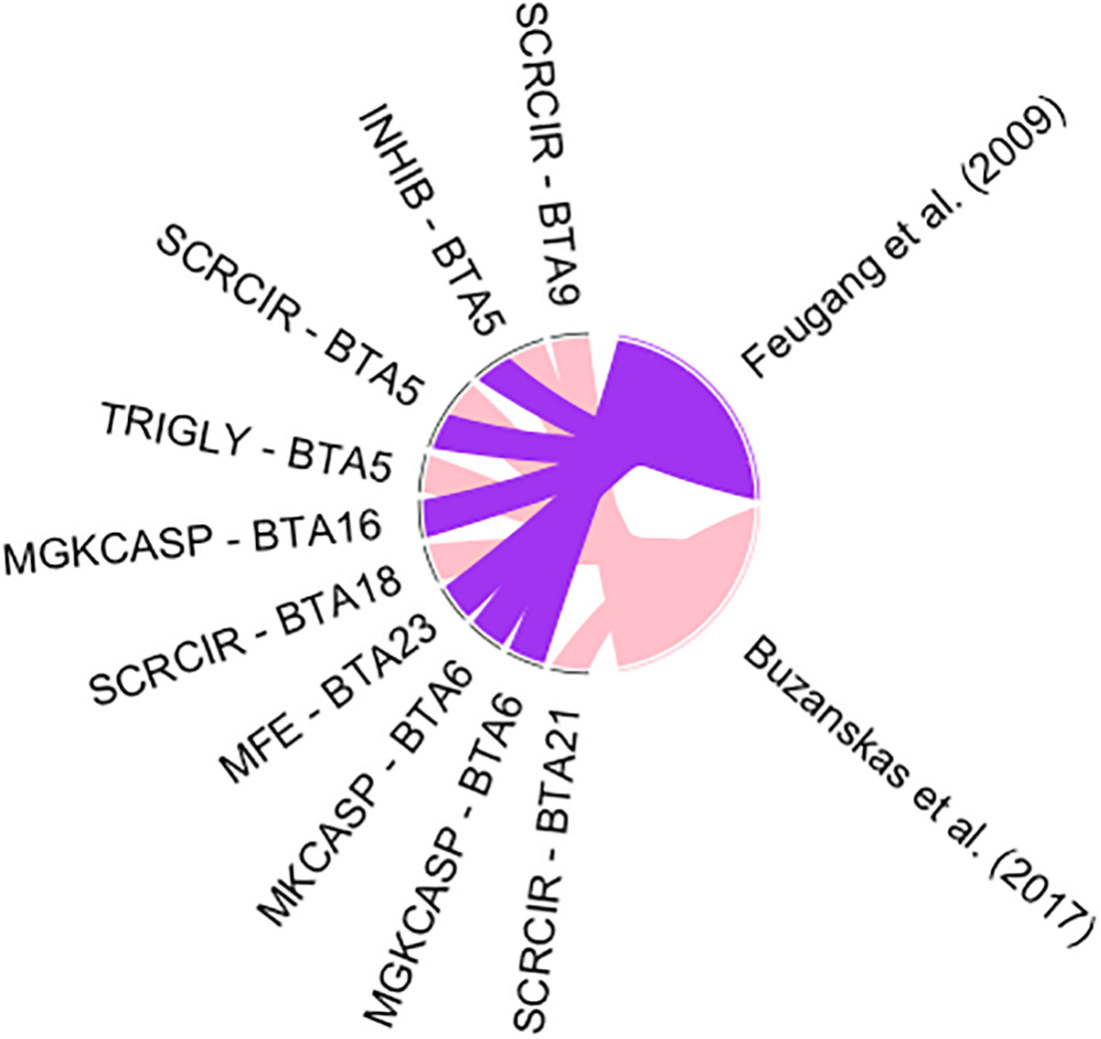
Chord plot showing the relationship between the top 10 enriched QTLs (scrotal circumference [SCRCIR], inhibin level [INHIB], triglyceride level [TRIGLY], milk glycosylated κ-casein percentage [MGKCASP], milk iron content [MFE], milk κ-casein percentage [MKCASP]) and the studies (Feugang et al. [15] in purple and Buzanskas et al. [14] in pink).

An interesting functionality of GALLO is the graphical visualization of the relationship between groups using a chord plot. The relationship_plot() function receives as arguments a dataframe (it can use the gene or QTL annotation results, the QTL enrichment, or any other table with 2 groups of information to be compared), the 2 groups to be compared (arguments x and y), and the graphical arguments to set the size, color, and gap between the sectors in the chord plot. Fig. 5 shows the chord plot obtained using a subset of the QTL annotation dataframe composed only by the top 10 enriched traits and the studies from which these traits were annotated. This plot indicates that only inhibin levels and scrotal circumference on BTA5 are shared between Feugang et al. and Buzanskas et al. [14, 15]. Additionally, milk glycosylated κ-casein percentage (BTA 6 and 16), milk κ-casein percentage (BTA 6), and milk iron content (BTA 23) were annotated only in Feugang et al. [15] and scrotal circumference (BTA 9, 18, 21) and triglyceride level (BTA 5) were annotated only in Buzanskas et al. [14]. Inhibin is produced by the Sertoli cells and can be used as a biomarker for sexual development [22]. In addition, the inhibin levels were already associated with both scrotal circumference and sperm quality traits in several studies, suggesting an important role in male fertility [23–27]. The results obtained here through the integration of the GWAS results from 2 independent studies followed by QTL annotation reinforce this association. Additionally, QTLs not associated with reproductive phenotypes were identified in the enrichment analysis, suggesting the presence of complex biological mechanisms such as a pleiotropic effect, epistasis, and genetic hitchhiking effect. Previous studies have highlighted the possible role of genomic regions with these kinds of processes in the cattle genome [28, 29]. An additional integration of the QTL annotation and enrichment analysis performed here with the gene annotation and prospection for functional candidate genes can be a powerful tool to better reveal the genetic architecture and the relationship among complex traits.

## Discussion

The GALLO package is composed of a group of functions designed to perform an efficient and direct downstream analysis for the gene and QTL annotation for candidate markers/SNPs, haplotypes, genomic windows, runs of homozygosity, CNVs, and so forth. The functions implemented in GALLO were designed to allow the integration of multiple datasets simultaneously. A brief summary of these functions is provided in Table 1. For example, GWAS results from multiple traits and/or populations or breeds can be analyzed together and compared or individually analyzed in the downstream analysis. This can be easily performed by adding an extra column in the input file with the grouping factors to classify each dataset. These input files can be easily adapted from the output of software packages that are commonly used to analyze high-throughput genomic data, such as PLINK, BLUPF90, and DESeq2 [30–32]. In addition, GALLO provides a set of functions designed for the visualization of the annotation results, overlap among groups, relationship between groups (e.g., markers and candidate genes, datasets and QTLs, models and positional candidate genes), and QTL enrichment results. This set of functions provides the capability of integrating several results from multiple sources including different methodologies (e.g., GWAS, RNA sequencing, proteomics), populations (e.g., breeds, time points), traits, or the different combination of these groups or others. Taken together, this set of functions provide the possibility to perform all the steps of gene/QTL annotation, comparison, and summary in the same environment. Additionally, the output obtained using GALLO was designed to allow a direct connection between the candidate genomic regions and the genes/QTLs that overlap those regions. Therefore, compared with outputs provided by other tools, such as biomaRt and Bedtools, the interpretation of the output provided by GALLO is straightforward and easy to handle. Finally, the QTL enrichment analysis available with GALLO is a useful and new approach that has the potential to better elucidate the relationship between candidate genomic regions and the target phenotype. It is important to highlight that despite the fact that GALLO was primarily designed for livestock species, the package can perform gene annotation and data comparison for any other species without any additional alterations to the input files. Regarding the QTL annotation and the respective graphical visualization, the user should provide the gff file from the QTL database in a format matching the gff files available on Animal QTLdb.

A summary of use examples and output descriptions for all the functions available on GALLO can be found in the reference manual (https://cran.r-project.org/web/packages/GALLO/GALLO.pdf). It is important to highlight that the 2 studies used as an example here are also part of the bovine QTL database. Consequently, the results obtained here for annotation and enrichment would be expected, once the candidate regions from the example file are present in the database used for the annotation. This approach was used as a proof of concept of the methodology and indicates a precise annotation of the candidate regions.

## Conclusion

The integration of multiple datasets for gene and QTL annotation is one of the major bottlenecks for the automatization of functional analysis of the results obtained using high-throughput methodologies. The GALLO package provides a user-friendly and straightforward environment to perform gene and QTL annotation, visualization, data comparison, and QTL enrichment for functional studies in livestock species. Consequently, the use of GALLO in the analyses of data generated from high-throughput methodologies may improve the identification of hidden patterns across datasets and data mining of previously reported associations, as well as efficiency in the examination of the genetic architecture of complex traits in livestock.

## Availability of Source Code and Requirements

Project name: Genomic Annotation in Livestock for positional candidate LOci (GALLO)

Project home page: https://github.com/pablobio/GALLO

Operating system(s): Platform independent

Programming language: R

Other requirements: Depends:  R (≥3.5.0)

License: GPL-3

RRID:SCR_019212

Bio.tools: biotools: genomic_annotation_in_livestock_for_ positional_candidate_loci_gallo

## Data Availability

All of the data analyzed in the present study can be accessed in the public repository hosting the R package [33]. The input files and results used as examples in the text are available in Supplementary Tables S1–S4. A manual including use examples and output descriptions for all the functions available on GALLO can be found in the package vignette [34]. An archival copy of the code and supporting data is available via the *GigaScience* repository, GigaDB [32].

## Additional Files

Supplementary Table S1. Genomic coordinates of the 527 markers used in the analysis for gene and QTL annotation.

Supplementary Table S2. Output from GALLO for gene annotation.

Supplementary Table S3. Output from GALLO for QTL annotation.

Supplementary Table S4. QTL enrichment results obtained using GALLO.

Supplementary File S1. Example of gff file used for QTL annotation on GALLO.

Supplementary File S2. Example of gtf file used for gene annotation on GALLO.

Supplementary File S3. R script with the commands used to produce the QTL and gene annotations, plots and enrichment analysis presented in the manuscri

## Abbreviations

Animal QTLdb: Animal Quantitative Trait Loci Database; BP: position in base pairs; BP1: start position in base pairs; BP2: end position in base pairs; CHR: Chromosome; CNV: copy number variation; FDR: false discovery rate; GALLO: Genomic Annotation in Livestock for positional candidate Loci; GWAS: genome-wide association study; kb: kilobase pairs; QTL: quantitative trait locus; RAM: random access memory; SNP: single-nucleotide polymorphism.

## Competing Interests

The authors declare that they have no competing interests.

## Funding

This study was supported by the Sustainable Beef and Forage Science Cluster (FDE.13.17) funded by the Canadian Beef Cattle Check-Off, Beef Cattle Research Council,  Alberta Beef Producers, Alberta Cattle Feeders’ Association, Beef Farmers of Ontario, La Fédération des Productuers de bovins du Québec, and Agriculture and Agri-Food Canada's Canadian Agricultural Partnership. The funders had no role in study design, data collection and analysis, decision to publish, or preparation of the manuscript.

## Authors' Contributions

P.A.S.F. and A.C. were responsible for the conceptualization. P.A.S.F., A.S.V., and A.C. were responsible for the data processing and review of the codes. P.A.S.F. and A.S.V. were responsible for data curation. P.A.S.F. and G.M. were responsible for the implementation of the bioinformatic pipeline, integration of datasets, and the coding. A.C. was responsible for funding acquisition.

## Supplementary Material

giaa149_GIGA-D-20-00265_Original_Submission

giaa149_GIGA-D-20-00265_Revision_1

giaa149_GIGA-D-20-00265_Revision_2

giaa149_Response_to_Reviewer_Comments_Original_Submission

giaa149_Response_to_Reviewer_Comments_Revision_1

giaa149_Reviewer_1_Report_Original_Submission Aniek Bouwman -- 9/20/2020 Reviewed

giaa149_Reviewer_1_Report_Revision_1 Aniek Bouwman -- 11/10/2020 Reviewed

giaa149_Reviewer_2_Report_Original_Submission Hussain Bahbahani -- 9/30/2020 Reviewed

giaa149_Reviewer_3_Report_Original_Submission Julian Taylor -- 10/5/2020 Reviewed

giaa149_Reviewer_4_Report_Original_Submission Daniel Fischer -- 10/5/2020 Reviewed

giaa149_Reviewer_4_Report_Revision_1 Daniel Fischer -- 10/30/2020 Reviewed

giaa149_Supplemental_Files

## References

[1] Ron M, Weller JI. From QTL to QTN identification in livestock - Winning by points rather than knock-out: A review. Anim Genet. 2007;38:429–39. 17697134 10.1111/j.1365-2052.2007.01640.x

[2] Ernst CW, Steibel JP. Molecular advances in QTL discovery and application in pig breeding. Trends Genet. 2013;29:215–24. 23498076 10.1016/j.tig.2013.02.002

[3] Miglior F, Fleming A, Malchiodi F, et al. A 100-Year review: Identification and genetic selection of economically important traits in dairy cattle. J Dairy Sci. 2017;100:10251–71. 29153164 10.3168/jds.2017-12968

[4] Pértille F, Guerrero-Bosagna C, da Silva VH, et al. High-throughput and cost-effective chicken genotyping using next-generation sequencing. Sci Rep. 2016;6, doi:10.1038/srep26929. PMC4879531 27220827

[5] Cánovas A, Reverter A, DeAtley KL, et al. Multi-tissue omics analyses reveal molecular regulatory networks for puberty in composite beef cattle. PLoS One. 2014;9:e102551. 25048735 10.1371/journal.pone.0102551 PMC4105537

[6] Hu ZL, Park CA, Reecy JM. Building a livestock genetic and genomic information knowledgebase through integrative developments of Animal QTLdb and CorrDB. Nucleic Acids Res. 2019;47:D701–10. 30407520 10.1093/nar/gky1084 PMC6323967

[7] Fonseca PAS, Id-Lahoucine S, Reverter A, et al. Combining multi-OMICs information to identify key-regulator genes for pleiotropic effect on fertility and production traits in beef cattle. PLoS One. 2018;13, doi:10.1371/journal.pone.0205295. PMC6193631 30335783

[8] Fonseca PAS, dos Santos FC, Lam S, et al. Genetic mechanisms underlying spermatic and testicular traits within and among cattle breeds: Systematic review and prioritization of GWAS results. J Anim Sci. 2018;96:4978–99. 30304443 10.1093/jas/sky382 PMC6276581

[9] Suárez-Vega A, Gutiérrez-Gil B, Benavides J, et al. Combining GWAS and RNA-Seq approaches for detection of the causal mutation for hereditary junctional epidermolysis bullosa in sheep. PLoS One. 2015;10:e0126416. 25955497 10.1371/journal.pone.0126416 PMC4425408

[10] Durinck S, Moreau Y, Kasprzyk A, et al. BioMart and Bioconductor: A powerful link between biological databases and microarray data analysis. Bioinformatics. 2005;21:3439–40. 16082012 10.1093/bioinformatics/bti525

[11] Quinlan AR, Hall IM. BEDTools: A flexible suite of utilities for comparing genomic features. Bioinformatics. 2010;26:841–2. 20110278 10.1093/bioinformatics/btq033 PMC2832824

[12] R Core Team. R: A language and environment for statistical computing. 2019. https://www.r-project.org. Accessed 1 April 2019.

[13] GALLO: Genomic Annotation in Livestock for Positional Candidate LOci. https://cran.r-project.org/web/packages/GALLO/index.html.Accessed 1 April 2019

[14] Buzanskas ME, Grossi DDA, Ventura RV, et al. Candidate genes for male and female reproductive traits in Canchim beef cattle. J Animal Sci Biotechnol. 2017;8:67. 10.1186/s40104-017-0199-8 PMC5569548 28852499

[15] Feugang JM, Kaya A, Page GP, et al. Two-stage genome-wide association study identifies integrin beta 5 as having potential role in bull fertility. BMC Genomics. 2009;10:176. 19393042 10.1186/1471-2164-10-176 PMC2684547

[16] Download data from the Cattle QTLdb. https://www.animalgenome.org/cgi-bin/QTLdb/BT/download?file=gffUMD_3.1. Accessed 1 April 2019

[17] Download data from the Ensembl cattle reference genome. ftp://ftp.ensembl.org/pub/release-94/gtf/bos_taurus/, . Accessed 1 April 2019.

[18] Genomic Annotation in Livestock for positional candidate LOci: GALLO. https://rpubs.com/pablo_bio/GALLO_vignette. Accessed 1 April 2019

[19] Hackinger S, Zeggini E. Statistical methods to detect pleiotropy in human complex traits. Open Biol. 2017;7:170125. 29093210 10.1098/rsob.170125 PMC5717338

[20] Id-Lahoucine S, Molina A, Cánovas A, et al. Screening for epistatic selection signatures: A simulation study. Sci Rep. 2019;9:1026. 30705409 10.1038/s41598-019-38689-2 PMC6355851

[21] Kühn C, Thaller G, Winter A, et al. Evidence for multiple alleles at the DGAT1 locus better explains a quantitative trait locus with major effect on milk fat content in cattle. Genetics. 2004;167:1873–81. 15342525 10.1534/genetics.103.022749 PMC1470998

[22] Phillips DJ . Activins, inhibins and follistatins in the large domestic species. Domest Anim Endocrinol. 2005;28, doi:10.1016/j.domaniend.2004.05.006. 15620803

[23] Fortes MRS, Reverter A, Kelly M, et al. Genome-wide association study for inhibin, luteinizing hormone, insulin-like growth factor 1, testicular size and semen traits in bovine species. Andrology. 2013;1:644–50. 23785023 10.1111/j.2047-2927.2013.00101.x

[24] Fortes MRS, Reverter A, Hawken RJ, et al. Candidate genes associated with testicular development, sperm quality, and hormone levels of inhibin, luteinizing hormone, and insulin-like growth factor 1 in Brahman bulls. Biol Reprod. 2012;87:58. 22811567 10.1095/biolreprod.112.101089

[25] Bame JH, Dalton JC, Degelos SD, et al. Effect of long-term immunization against inhibin on sperm output in bulls. Biol Reprod. 1999;60:1360–6. 10330093 10.1095/biolreprod60.6.1360

[26] Martin TL, Williams GL, Lunstra DD, et al. Immunoneutralization of inhibin modifies hormone secretion and sperm production in bulls. Biol Reprod. 1991;45:73–77. 1715195 10.1095/biolreprod45.1.73

[27] Sato T, Kudo T, Ikehara Y, et al. Chondroitin sulfate N-acetylgalactosaminyltransferase 1 is necessary for normal endochondral ossification and aggrecan metabolism. J Biol Chem. 2011;286:5803–12. 21148564 10.1074/jbc.M110.159244 PMC3037693

[28] Bolormaa S, Pryce JE, Reverter A, et al. A multi-trait, meta-analysis for detecting pleiotropic polymorphisms for stature, fatness and reproduction in beef cattle. PLoS Genet. 2014;10, doi:10.1371/journal.pgen.1004198. PMC3967938 24675618

[29] Love MI, Huber W, Anders S. Moderated estimation of fold change and dispersion for RNA-seq data with DESeq2. Genome Biol. 2014;15(12):550. 25516281 10.1186/s13059-014-0550-8 PMC4302049

[30] Purcell S, Neale B, Todd-Brown K, et al. PLINK: A tool set for whole-genome association and population-based linkage analyses. Am J Hum Genet. 2007;81:559–75. 17701901 10.1086/519795 PMC1950838

[31] Misztal I, Tsuruta S, Strabel T, et al. BLUPF90 and related programs (BGF90). In: Proc. 7th World Congress on Genetics Applied to Livestock Production, Montpellier, France. 2002:743–4.

[32] Fonseca PAS, Suárez-Vega A, Marras G, et al. GALLO: An R package for genomic annotation and integration of multiple data source in livestock for positional candidate loci. GigaScience Database 2020. 10.5524/100834. PMC7772745 33377911

[33] Github repository for GALLO project. https://github.com/pablobio/GALLO. Accessed 1 April 2019

[34] Genomic Annotation in Livestock for positional candidate LOci: GALLO. https://cran.r-project.org/web/packages/GALLO/vignettes/GALLO.html. Accessed 1 April 2019

