# Supplementary material for: GALLO: An R package for genomic annotation and integration of multiple data sources in livestock for positional candidate loci
Source: Gigascience. 2020 Dec 30;9(12):giaa149. doi: 10.1093/gigascience/giaa149 (PMC7772745; doi:10.1093/gigascience/giaa149)
Supplement: giaa149_GIGA-D-20-00265_Original_Submission [file giaa149_giga-d-20-00265_original_submission.pdf]

GigaScience

GALLO: An R package for Genomic Annotation and integration of multiple data source in livestock for positional candidate LOci

--Manuscript Draft--

**Manuscript Number:**	GIGA-D-20-00265
**Full Title:**	GALLO: An R package for Genomic Annotation and integration of multiple data source in livestock for positional candidate LOci
**Article Type:**	Technical Note
**Funding Information:**	Sustainable Beef and Forage Science Cluster funded by the Canadian Beef Cattle Check-Off, Beef Cattle Research Council, Alberta Beef Producers, Alberta Cattle Feeders' Association, Beef Farmers of Ontario, La Fédération des Productuers de bovins du Québec, and Agriculture and Agri-Food Canada's Canadian Agricultural Partnership (FDE.13.17) — Dr Angela Cánovas
**Abstract:**	The development of high-throughput sequencing and genotyping methodologies and precision livestock farming allowed the identification of thousands of genomic regions associated with several complex traits. The integration of multiple sources of biological information is a crucial step to better understand patterns regulating the development of complex traits. Genomic Annotation in Livestock for positional candidate LOci (GALLO) is an R package, for the accurate annotation of genes and quantitative trati loci (QTLs) located in regions identified in the most common genomic analyses performed in livestock, such as Genome-Wide Association Studies and transcriptomics using RNA-Sequencing. Moreover, GALLO allows the graphical visualization of gene and QTL annotation results, data comparison among different grouping factors (e.g., methods, breeds, tissues, statistical models, studies, etc.), and QTL enrichment in different livestock species including cattle, pigs, sheep, chicken, etc. Consequently, GALLO is a useful package for annotation, identification of hidden patterns across datasets, datamining of previous reported associations, as well as the efficient scrutinization of the genetic architecture of complex traits in livestock.
**Corresponding Author:**	Pablo Augusto de Souza Fonseca<br>University of Guelph<br>Guelph, ON CANADA
**Corresponding Author Secondary Information:**	
**Corresponding Author's Institution:**	University of Guelph
**Corresponding Author's Secondary Institution:**	
**First Author:**	Pablo Augusto de Souza Fonseca
**First Author Secondary Information:**	
**Order of Authors:**	Pablo Augusto de Souza Fonseca
	Aroa Suárez-Vega
	Gabiele Marras
	Angela Cánovas
**Order of Authors Secondary Information:**	
**Additional Information:**	
**Question**	**Response**
Are you submitting this manuscript to a	No

Powered by Editorial Manager® and ProduXion Manager® from Aries Systems Corporation

special series or article collection?	
**Experimental design and statistics**<br><br>Full details of the experimental design and statistical methods used should be given in the Methods section, as detailed in our Minimum Standards Reporting Checklist. Information essential to interpreting the data presented should be made available in the figure legends.<br><br>Have you included all the information requested in your manuscript?	Yes
**Resources**<br><br>A description of all resources used, including antibodies, cell lines, animals and software tools, with enough information to allow them to be uniquely identified, should be included in the Methods section. Authors are strongly encouraged to cite Research Resource Identifiers (RRIDs) for antibodies, model organisms and tools, where possible.<br><br>Have you included the information requested as detailed in our Minimum Standards Reporting Checklist?	Yes
**Availability of data and materials**<br><br>All datasets and code on which the conclusions of the paper rely must be either included in your submission or deposited in publicly available repositories (where available and ethically appropriate), referencing such data using a unique identifier in the references and in the "Availability of Data and Materials" section of your manuscript.<br><br>Have you have met the above requirement as detailed in our Minimum Standards Reporting Checklist?	Yes

Powered by Editorial Manager® and ProduXion Manager® from Aries Systems Corporation

Click here to access/download;Manuscript;manuscript_Gigascience_01_09_2

GALLO: An R package for Genomic Annotation and integration of multiple data source in livestock for positional candidate LOci

Pablo A.S. Fonseca[1*], Aroa Suárez-Vega[1], Gabriele Marras[1,2], and Ángela Cánovas[1*]

[1]University of Guelph, Department of Animal Biosciences, Centre for Genetic Improvement of Livestock, Guelph, N1G 2W1, Ontario, Canada.

[2]The Semex Alliance, Guelph N1G 3Z2, Ontario, Canada

Contact:

PASF: pfonseca@uoguelph.ca

ASV: asuarezv@uoguelph.ca

GM: gmarras@uoguelph.ca

AC: acanovas@uoguelph.ca

[*] Corresponding author

Abstract

The development of high-throughput sequencing and genotyping methodologies and precision livestock farming allowed the identification of thousands of genomic regions associated with several complex traits. The integration of multiple sources of biological information is a crucial step to better understand patterns regulating the development of complex traits. Genomic Annotation in Livestock for positional candidate LOci (GALLO) is an R package, for the accurate annotation of genes and quantitative trati loci (QTLs) located in regions identified in the most common genomic analyses performed in livestock, such as Genome-Wide Association Studies and transcriptomics using RNA-Sequencing. Moreover, GALLO allows the graphical visualization of gene and QTL annotation results, data comparison among different grouping factors (e.g., methods, breeds, tissues, statistical models, studies, etc.), and QTL enrichment in different livestock species including cattle, pigs, sheep, chicken, etc. Consequently, GALLO is a useful package for annotation, identification of hidden patterns across datasets, datamining of previous reported associations, as well as the efficient scrutinization of the genetic architecture of complex traits in livestock.

Keywords: Multi-omics integration; QTL annotation; Gene annotation; Datamining; QTL enrichment analysis; Livestock

Background

The identification of quantitative trait loci (QTLs), genomic regions linked to complex traits through association tests using genetic markers and phenotypic traits, is a crucial step in the improvement of genomic selection and economic profitability in livestock [1–4]. Additionally, in the last decades, the development of precision livestock farming strategies resulted in the possibility to obtain a huge volume and diversity of phenotypic data [5–7]. The development of high-throughput methodologies (e.g., Genome-Wide Association Studies, Transcriptomics, Metabolomics, Proteomics, etc.) for the study of the genetic architecture of complex traits allows the identification of potential candidate genes associated with economically relevant traits in livestock. Taken together, these new technologies can substantially improve the accuracy of detection of candidate regions associated with economical important traits across the genome in livestock species [8]. Consequently, the number of QTLs identified across the genome in livestock species increased substantially in the last years. Currently, in the Animal QTLdb it is possible to retrieve information about QTLs previously identified in cattle (127,191), chicken (11,340), horse (2,260), pig (29,865), rainbow trout (584) and sheep (3,001) [9]. The proper integration of the results obtained from different methodologies and technologies available is a crucial step for the accurate identification of the biological processes regulating the development of complex traits as well as the identification of potential functional candidate genes for each trait or shared among traits [8,10–12]. The integration of both structural and functional data can help to scrutinize the genetic architecture of economically relevant traits, consequently, helping to better understand complex biological patterns regulating the expression of these traits, such as pleiotropic effect, epistasis and genetic hitchhiking, among others.

In spite of the great potential to improve the identification of functional candidate genes and/or QTLs through the integration of multiple data sources, currently this process is not very straightforward due to limitations in the pipelines and algorithms implemented in the tools available for livestock. Currently, there are several tools that implement functions for gene (i.e., Biomart and BEDTools) and QTL annotation (Animal QTLdb) [9,13,14]. However, these tools have limitations regarding the automatization process to analyze results from multiple candidate regions (Biomart web application and the R package and Animal QTLdb) or for the visualization of the results. Moreover, although the automatization is possible, the direct link between the candidate regions and/or markers with the annotated genes and QTLs is missed. Consequently, this gap is forcing the user to back solve the overlap between the input and output files in order to perform the proper association between the candidate region and/or markers and the annotated genes and/or positional co-localized QTLs. In addition, nowadays there is still a gap for customized QTL enrichment analyses in the available software and databases. The Genomic functional Annotation in Livestock for positional candidate LOci (GALLO) is an R package designed to provide an automatized and a straightforward environment for gene and QTL annotation in multiple candidate regions, as well as data integration from multiple data sources. The QTL enrichment analyses can additionally be performed directly by GALLO using the output obtained from the QTL annotation step. In addition, GALLO also provides a set of functions for graphical visualization of the annotation, comparison, integration and QTL enrichment results. In this context, the GALLO package was developed as an alternative tool: 1) to allow the integration and the simultaneous annotation of multiple datasets for genes and QTLs; 2) to provide graphical visualization tools to integrate visually the annotation and the similarity against the datasets; 3) to

perform QTL enrichment analysis for the positional candidate genomic regions and/or markers associated with economically relevant traits in livestock.

Implementation

The GALLO package was wrote in the R language [15]. The stable release is available as an R package on CRAN (https://cran.r-project.org/web/packages/GALLO/index.html). The code was extensively tested with several datasets from different sources and methodologies and reviewed to ensure the package quality standards. Additionally, the vignettes were created to be comprehensive and to present practical examples in order to provide a user-friendly and an easier understanding and usability for the user.

The GALLO package provides a useful set of functions that allows the user a straightforward data integration, comparison, gene and QTL annotation, and visualization of several data sources and methodologies, such as data from genome-wide association study (GWAS), RNA-Sequencing, whole-genome sequencing, etc. (Figure 1 and Table 1). The main advantage to perform an automated analysis from multiple datasets results in the flexibility to handle the output using different subsets (traits, populations, models, etc.) in the same environment, without generating multiple intermediate output files.

Methods

Case study – Candidate regions for scrotal circumference and fertility in cattle

The dataset used to present the basic usage and advantages of GALLO package is composed by the markers significantly associated with scrotal circumference in Canchim breed [16] and uncompensable fertility in Holstein cattle [17]. These two studies were previously analyzed

together in a systematic review regarding male fertility in cattle [11]. Therefore, the data used herein comprises a multi-study and multi-breed analysis. These candidate markers (527 single nucleotide polymorphisms (SNPs)) are available on Supplementary Table 1. In addition to the candidate markers, we present as Supplementary Files 1 and 2, the annotation gff file containing the QTL database information for cattle (obtained from the Animal QTLdb; https://www.animalgenome.org/cgi-bin/QTLdb/BT/download?file=gffUMD_3.1) and the gtf file containing the genes annotated in the cattle genome obtained from Ensembl (ftp://ftp.ensembl.org/pub/release-94/gtf/bos_taurus/). The genomic coordinates of both files were based on the bovine reference genome version UMD 3.1 due to the original coordinates used to report the location of the candidate markers in the original studies. Here, the analysis performed follows the same logical order to the one presented in the GALLO vignette (https://rpubs.com/pablo_bio/GALLO_vignette). However, the dataset used in the user practical tutorial is a subset of the data presented here, aiming to reduce computational demand for the users. The script with all the commands used to perform the analysis present here are available in Supplementary File 3. All the tests were performed using a desktop with a processor Intel Core i5 2.4 GHz with 8 Gb of RAM memory.

Importing datasets and annotating genes and QTLs around candidate markers

The first step in the pipeline consists in importing the databases which will be used for the analyses with the *import_gff_gtf()* function. In our specific example, we imported both, cattle gene annotation (gtf) and QTL (gff) databases. The *import_gff_gtf()* function receives as arguments the database file (db_file) and the file type (file_type= gff or gtf) and creates a dataframe with the respective information from each file. The system time demanded to import the gtf and gff files were 0.045 and 0.311 seconds, respectively, indicating an efficient importing process. The file

containing the candidate markers can be imported using any available function in the R environment such as *read.table()* and *read.csv()*.

The main function of GALLO, *find_genes_qtls_around_markers()*, is responsible to perform the annotation of genes and/or co-localized QTLs within or nearby candidate markers or genomic regions (using a user's defined interval/window). This function uses the information provided in the .gtf file (for gene annotation) or .gff (for QTL annotation) to retrieve the requested information. The output combines the information available in the input file provided by the user with the information available for the genes and QTLs mapped in the candidate genomic regions. Consequently, for example, for an input file composed of three genomic coordinates where 4 genes are annotated in each of the intervals determined by the user, the output file of *find_genes_qtls_around_markers()* will contain 12 rows. The minimum information necessary for the gene and QTL annotation procedures is a data frame with two columns with the chromosome (CHR) and position in base pairs (BP) in the case of candidate SNPs input. In the case of candidate haplotypes, windows, copy number variations (CNVs) or candidate regions; the input file is composed by three columns corresponding to the chromosome (CHR), the start position in base pairs (BP1) and the end position in base pairs (BP2). Data examples for the candidate markers and windows input files can be obtained using the data(QTLmarkers) and data(QTLwindows) commands in R. Additionally, examples of QTL and gene annotation results are accessible through the commands data(gtfGenes) and data(gffQTLs) commands, respectively. These outputs can be easily handled by summary functions in R, such as *table()*, to obtain information such as the total number of genes and QTLs, the number of genes and QTLs annotated per variants, etc. The performance of GALLO package in terms of efficiency is similar to other currently packages and software which allow a similar annotation of candidate genomic loci. In

this sense, the gene annotation process was compared with the *getBM()* function from the biomaRt package. The gene annotation process on GALLO needed 0.424 seconds to completely annotate the genes in a 200 Kb interval (upstream and dowstream) from candidate markers, while the biomaRt function required 0.019 seconds. The QTL annotation on GALLO was compared with the Bedtools -wao -C command, resulting in 0.851 and 0.12 seconds required for each approach, respectively. It is important to highlight that for both gene and QTL annotation using biomaRt and bedtools, respectively, a posterior processing of the output file is required in order to match the candidate markers and the genes and QTLs mapped within the candidate intervals. On the order hand, the output file from *find_genes_qtls_around_markers()* function was designed to allow this match in an intuitive way, combining the rows of both candidate markers file and database files (gff and gtf). Additionally, GALLO allows the user to perform both annotations for genes and QTLs with a single software and programming language. Consequently, GALLO obtains a more elaborate and informative output with compromise the computational demand for the analysis. The output files obtained in the gene and QTL annotation are available on Supplementary Tables 2 and 3, respectively.

Comparing and visualizing the overlapping of genes and QTLs annotated within the candidate regions

The output file generated by the *find_genes_qtls_around_markers()* function can be used as an input file for the other set of GALLO functions. An advantage from the output of *find_genes_qtls_around_markers()* function is any additional information present in the input file will be retained in the output file. Consequently, this information can be used compare the retrieved information between groups of population, methodologies, statistical models, etc. For example, the functions *overlapping_among_groups()* and *plot_overlapping()* can be used to create matrices

with the overlapping values among groups and to visualize this overlapping. The Figure 2 shows the results of gene and QTL overlapping between the positional markers obtained in the two studies selected for the dataset of markers analyzed, Feugang et al. (2009) [17] and Buzanskas et al. (2017) [16]. It is important to highlight that these overlapping matrices are not symmetrical. The percentage of genes from study A shared with the study B, and vice-versa, are calculated in function of the total number of genes in A or B, respectively. In the current example, it is possible to note that only a small percentage of the positional candidate genes were shared between the studies. However, the analysis of QTL (using the trait name as reference ID) overlapping indicated a higher similarity between the studies, 46% of the all the QTLs annotated in the candidate regions from Feugang et al. (2010) [17] were also present in Buzanskas et al. (2017) [16] and 93% of the QTLs annotated in the candidate regions from Buzanskas et al. (2017) were also present in Feugang et al. (2010) [16,17]. These results may suggest that even with a small proportion of shared genes, the candidate regions of both studies are frequently associated with similar processes. Similar roles played by the positional candidate genes in those regions in related biological process would be one of the reasons of the observed result.

Understanding the QTL context of the candidate regions

A more precise investigation of the QTL representativeness and diversity can help to better understand the genomic context of the candidate regions. The recurrent association of particular genomic regions with multiple traits might suggest the presence of complex genetic mechanisms regulating that region, such as pleiotropy, epistasis, hitchhiking effect, among others [18,19]. The *plot_qtl_info()* function from GALLO allows the graphical visualization of the summary of QTL types and traits annotated. The percentage of each QTL type annotated within the candidate regions is presented in a pie plot through the use of the argument qtl_plot=”qtl_type”, while the percentage

of each trait associated with a specific QTL type can be plotted setting the argument qtl_plot=”qtl_name” and informing the additional argument qtl_class (that must receive the name of the QTL class to be plotted). Figure 3 shows that for Feugang et al. (2009) [17] the two most frequent QTL types were Milk (50.42%) and Reproduction (16.97%), while for Buzanskas et al. (2017) [16] the most frequent QTL types were Reproduction (87.06%) and Meat and Carcass (5.03%). An in depth analyses can be performed for each QTL type in order to observe the frequency of each trait associated with a specific QTL type. The most frequent traits related with Reproduction QTLs were calving ease (>3%) and scrotal circumference (>60%) for Feugang et al. (2009) and Buzanskas et al. (2017) [16,17], respectively (Figure 3). The comparison between the frequency of traits related with Reproduction QTLs annotated in Feugang et al. (2009) and Buzanskas et al. (2017) [16,17] indicated that among the top 10 more frequent QTLs, calving ease, inhibin levels, stillbirth, interval to first estrus after calving, and birth index were shared between the studies. The combined analysis (not filtering by study) indicated that the Reproduction and Milk QTL types were the two most frequent classes with 76.99% and 10.62% of all QTL types, respectively. In addition, scrotal circumference, inhibin level and calving easy were the most frequent Reproduction QTL related traits in the combined analysis.

QTL enrichment analysis

In some cases, the biases produced by a greater research into certain areas/traits of higher relevance to animal production (such as milk production related traits in the QTL database for cattle) may result in a larger proportion of records for these traits in the QTL database. Consequently, the simple investigation of the proportion of each QTL type might not be totally useful. The GALLO package allows the user to perform a QTL enrichment analysis to test the significance of the QTL representativeness. The QTL enrichment analysis function on GALLO package is based in a

hypergeometric test approach, where the number of QTLs annotated within the candidate regions, for each QTL type or trait, is compared with the observed number of QTLs in the reference database. The *qtl_enrich()* function allow the user to perform the QTL enrichment analysis for both QTL types and traits (qtl_type= “QTL_type” or “Name”), for the whole genome or chromosome-wise (enrich_type= “genome” or “chromosome”) and for all the annotated chromosomes or a subset (chr.subset= NULL or the object with the subset of chromosomes). The use of chromosome-wise enrichment analysis might help to detect specific regions across the genome with high number of QTLs for some specific trait, i.e. BTA14 in cattle for milk production [20]. A total of 161 unique pairs of traits and chromosomes were tested for the enrichment using the annotated QTLs for both studies. The system time required to perform the enrichment analysis was 5.32 seconds, suggesting efficient processing. The top 10 enriched QTLs (False Discovery Rate (FDR) < 0.05) for the combined analysis are shown in Table 2 and the enrichment results for all the annotated QTLs are shown in Supplementary Table 4. Additionally, GALLO also allows the user to obtain a graphical visualization, in a bubble plot, of the enrichment results using the *QTLenrich_plot()* function. This function received as arguments the enriched table obtained from *qtl_enrich()*, the name of a column with the traits names to be plotted and the name of a column with the p-values to be plotted. A total of 28 pairs of traits and chromosomes were found enriched in the combined analysis, with scrotal circumference (BTA 5, 18, 9, and 21), milk glycosylated kappa-casein percentage (BTA 6 and 16), inhibin level (BTA 5), triglyceride level (BTA 5), milk kappa-casein percentage (BTA 6) and milk iron content (BTA 23) in the list of top 10 most enriched traits (Figure 4).

Relationship between studies and enriched QTLs

An interesting functionality of GALLO is the graphical visualization of the relationship between groups using a chord plot. The *relationship_plot()* function receives as argument a dataframe (it can used the gene or QTL annotation results, the QTL enrichment, or any other table with two groups of information to be compared), the two groups to be compared (arguments x and y) and the set graphical arguments or set the size, color and gap between the sector in the chord plot. The Figure 5 shows the chord plot obtained using a subset of the QTL annotation dataframe composed only by the top 10 enriched traits and the studies which these traits were annotated. This plot indicated that only inhibin levels and scrotal circumference on BTA5 are shared between Feugang et al. (2009) and Buzanskas et al. (2017) [16,17]. Additionally, milk glycosylated kappa-casein percentage (BTA 6 and 16), milk kappa-casein percentage (BTA 6) and milk iron content (BTA 23) were annotated only in Feugang et al. (2009) [17] and scrotal circumference (BTA 9, 18, 21) and triglyceride level (BTA 5) were annotated only in Buzanskas et al. (2017) [16]. Inhibin is produced by the Sertoli cells and can be used as a biomarker for sexual development [21]. In addition, the inhibin levels were already associated with both scrotal circumference and sperm quality traits in several studies, suggesting an important role in male fertility [22–26]. The results obtained here through the integration of the GWAS results from two independent studies followed by QTL annotation reinforce this association. Additionally, QTLs not associated with Reproduction phenotypes were identified in the enrichment analysis, suggesting the presence of complex such as pleiotropic effect, epistasis and genetic hitchhiking effect. Previous studies already highlight the possible role of genomic regions with this kind of processes in the cattle genome [27,28]. An additional integration of the QTL annotation and enrichment analysis performed here with the gene annotation and prospection for functional candidate genes can be a

powerful tool to better understand the genetic architecture and the relationship among complex traits.

Discussion

The GALLO package is composed of a group of functions designed to perform an efficient and direct downstream analysis for the gene and QTL annotation for candidate markers/SNPs, haplotypes, genomic windows, runs of homozygosity, CNVs, etc. The functions implemented in GALLO were designed in order to allow the integration of multiple datasets simultaneously. A brief summary of these functions is shown in Table 1. For example, GWAS results from multiple traits and/or populations or breeds can be analyzed together and compared or individually analyzed in the downstream analysis. This can be easily performed by adding an extra column in the input file with the grouping factors to classify each dataset. These input files can be easily adapted from the output of the most common used software to analyze high-throughput genomic data, such as PLINK, BLUPF90, DESeq2, etc. [29–31]. In addition, GALLO provides a set of functions designed for the visualization of the annotation results, overlapping among groups, relationship between groups (i.e., markers and candidate genes, datasets and QTLs, models and positional candidate genes, etc.), and QTL enrichment results. This set of functions provides the user the capability to integrate several results from multiple sources including different methodologies (GWAS, RNA-sequencing, proteomics, etc.), populations (breeds, time-points, etc.), traits or the different combination of these groups or others.

A summary of usage examples and output descriptions, for all the functions available on GALLO can be find in the reference manual (Supplementary File 4). It is important to highlight that the two studies used as an example here are also part of the bovine QTL database present in the Animal

QTLdb. Consequently, the results obtained here for annotation and enrichment would be expected, once the candidate regions are necessarily present in the annotation database. This approach was used as proof of concept of the methodology and indicates a precise annotation of the candidate regions.

Conclusion

The integration of multiple datasets for gene and QTL annotation is one of the major bottlenecks for the automatization of functional analysis of the results obtained using high-throughput methodologies. The GALLO package provides a user-friendly and straightforward environment to perform gene and QTL annotation, visualization, data comparison and QTL enrichment for functional studies in livestock species. Consequently, the use of GALLO in the analysis of data generated from high-throughput methodologies may improve the identification of hidden patterns across datasets, datamining of previous reported associations, as well as the efficiency in the scrutinization of the genetic architecture of complex traits in livestock.

Availability and requirements

Project name: Genomic Annotation in Livestock for positional candidate LOci (GALLO)

Project home page: https://github.com/pablobio/GALLO

Operating system(s): Platform independent

Programming language: R

Other requirements: Depends: R (>= 3.5.0)

License: GPL-3

Availability of supporting data

All the data analyzed in the present study can be accessed in the public repository hosting the R package (https://github.com/pablobio/GALLO). The input files and results used as examples in the manuscript preparation are available in the supplementary Tables 1-4. A manual including usage examples and output descriptions, for all the functions available on GALLO can be find in the reference manual (Supplementary File 4).

Declarations

List of abbreviations

BP: position in base pairs; BP1: start position in base pairs; BP2: end position in base pairs; CHR: Chromosome; CNV: Copy Number Variation; GALLO: Genomic Annotation in Livestock for positional candidate Loci; GWAS: Genome-Wide Association Study; QTL: Quantitative trait loci; SNP: Single Nucleotide Polymorphism.

Ethics approval and consent to participate

Not applicable.

Consent for publication

Not applicable.

Competing interests

The authors declare that they have no competing interests.

Funding

This study (FDE.13.17) was supported by the Sustainable Beef and Forage Science Cluster funded by the Canadian Beef Cattle Check-Off, Beef Cattle Research Council, Alberta Beef Producers, Alberta Cattle Feeders' Association, Beef Farmers of Ontario, La Fédération des Productuers de bovins du Québec, and Agriculture and Agri-Food Canada's Canadian Agricultural Partnership. The funders had no role in study design, data collection and analysis, decision to publish, or preparation of the manuscript.

Authors' contributions

PASF, ASV and AC were responsible for the conceptualization, data processing and review of the codes. PASF and ASV were responsible for data curation. PASF and GM were responsible to implement the bioinformatic pipeline, integrate datasets, and the coding. AC was responsible for funding acquisition.

Acknowledgements

Not applicable.

References

1. Ron M, Weller JI. From QTL to QTN identification in livestock - Winning by points rather than knock-out: A review. Anim. Genet. 2007.

2. Ernst CW, Steibel JP. Molecular advances in QTL discovery and application in pig breeding. Trends Genet. 2013.

3. Miglior F, Fleming A, Malchiodi F, Brito LF, Martin P, Baes CF. A 100-Year Review: Identification and genetic selection of economically important traits in dairy cattle. J Dairy Sci. 2017;

4. Pértille F, Guerrero-Bosagna C, Silva VH Da, Boschiero C, Nunes JDRDS, Ledur MC, et al. High-throughput and Cost-effective Chicken Genotyping Using Next-Generation Sequencing. Sci Rep. 2016;

5. Berckmans D. General introduction to precision livestock farming. Anim Front. 2017;

6. Halachmi I, Guarino M. Editorial: Precision livestock farming: A “per animal” approach using advanced monitoring technologies. Animal. 2016;

7. Banhazi TM, Lehr H, Black JL, Crabtree H, Schofield P, Tscharke M, et al. Precision Livestock Farming: An international review of scientific and commercial aspects. Int J Agric Biol Eng. 2012;5:1–9.

8. Cánovas A, Reverter A, DeAtley KL, Ashley RL, Colgrave ML, Fortes MRS, et al. Multi-tissue omics analyses reveal molecular regulatory networks for puberty in composite beef cattle. PLoS One. 2014;

9. Hu ZL, Park CA, Reecy JM. Building a livestock genetic and genomic information knowledgebase through integrative developments of Animal QTLdb and CorrDB. Nucleic Acids Res. 2019;

10. De Souza Fonseca PA, Id-Lahoucine S, Reverter A, Medrano JF, Fortes MS, Casellas J, et al. Combining multi-OMICs information to identify key-regulator genes for pleiotropic effect on fertility and production traits in beef cattle. PLoS One. 2018;13:1–22.

11. Fonseca PA de S, dos Santos FC, Lam S, Suárez-Vega A, Miglior F, Schenkel FS, et al. Genetic mechanisms underlying spermatic and testicular traits within and among cattle breeds: Systematic review and prioritization of GWAS results. J Anim Sci. 2018;

12. Suárez-Vega A, Gutiérrez-Gil B, Benavides J, Perez V, Tosser-Klopp G, Klopp C, et al. Combining GWAS and RNA-Seq approaches for detection of the causal mutation for hereditary

junctional epidermolysis bullosa in sheep. PLoS One. 2015;

13. Durinck S, Moreau Y, Kasprzyk A, Davis S, De Moor B, Brazma A, et al. BioMart and Bioconductor: A powerful link between biological databases and microarray data analysis. Bioinformatics. 2005;

14. Quinlan AR, Hall IM. BEDTools: A flexible suite of utilities for comparing genomic features. Bioinformatics. 2010;

15. R Core Team (2019). R: A language and environment for statistical computing. Accessed 1st April 2019. 2019;

16. Buzanskas ME, Grossi D do A, Ventura RV, Schenkel FS, Chud TCS, Stafuzza NB, et al. Candidate genes for male and female reproductive traits in Canchim beef cattle. J Anim Sci Biotechnol. 2017;

17. Feugang JM, Kaya A, Page GP, Chen L, Mehta T, Hirani K, et al. Two-stage genome-wide association study identifies integrin beta 5 as having potential role in bull fertility. BMC Genomics. 2009;

18. Hackinger S, Zeggini E. Statistical methods to detect pleiotropy in human complex traits. Open Biol. 2017.

19. Id-Lahoucine S, Molina A, Cánovas A, Casellas J. Screening for epistatic selection signatures: A simulation study. Sci Rep. 2019;

20. Kühn C, Thaller G, Winter A, Bininda-Emonds ORP, Kaupe B, Erhardt G, et al. Evidence for multiple alleles at the DGAT1 locus better explains a quantitative trait locus with major effect on milk fat content in cattle. Genetics. 2004;

21. Phillips DJ. Activins, inhibins and follistatins in the large domestic species. Domest. Anim. Endocrinol. 2005.

22. Fortes MRS, Reverter A, Kelly M, Mcculloch R, Lehnert SA. Genome-wide association study for inhibin, luteinizing hormone, insulin-like growth factor 1, testicular size and semen

traits in bovine species. Andrology. 2013;

23. Fortes MRS, Reverter A, Hawken RJ, Bolormaa S, Lehnert S a. Candidate genes associated with testicular development, sperm quality, and hormone levels of inhibin, luteinizing hormone, and insulin-like growth factor 1 in Brahman bulls. Biol Reprod [Internet]. 2012 [cited 2013 Sep 6];87:58. Available from: http://www.ncbi.nlm.nih.gov/pubmed/22811567

24. Bame JH, Dalton JC, Degelos SD, Good TEM, Ireland JLH, Jimenez-Krassel F, et al. Effect of Long-Term Immunization against Inhibin on Sperm Output in Bulls1. Biol Reprod. 1999;

25. Martin TL, Williams GL, Lunstra DD, Ireland JJ. Immunoneutralization of Inhibin Modifies Hormone Secretion and Sperm Production in Bulls1. Biol Reprod. 1991;

26. Sato T, Kudo T, Ikehara Y, Ogawa H, Hirano T, Kiyohara K, et al. Chondroitin sulfate N-acetylgalactosaminyltransferase 1 is necessary for normal endochondral ossification and aggrecan metabolism. J Biol Chem. 2011;

27. De Souza Fonseca PA, Id-Lahoucine S, Reverter A, Medrano JF, Fortes MS, Casellas J, et al. Combining multi-OMICs information to identify key-regulator genes for pleiotropic effect on fertility and production traits in beef cattle. PLoS One. 2018;

28. Bolormaa S, Pryce JE, Reverter A, Zhang Y, Barendse W, Kemper K, et al. A Multi-Trait, Meta-analysis for Detecting Pleiotropic Polymorphisms for Stature, Fatness and Reproduction in Beef Cattle. PLoS Genet. 2014;10.

29. Love MI, Huber W, Anders S. Moderated estimation of fold change and dispersion for RNA-seq data with DESeq2. Genome Biol. 2014;15:1–21.

30. Purcell S, Neale B, Todd-Brown K, Thomas L, Ferreira MAR, Bender D, et al. PLINK: A tool set for whole-genome association and population-based linkage analyses. Am J Hum Genet. 2007;

31. Misztal I, Tsuruta S, Strabel T, Auvray B, Druet T, H LD. BLUPF90 and related programs (BGF90). Proc 7th world Congr Genet Appl to Livest Prod. 2002. p. 743–4.

Tables

Table 1: Description of the functions implemented in the GALLO package.

Function	Description	Output
**Gene and QTL annotation**		
import_gff_gtf	Import the gff and gtf files used for QTL and gene annotation, respectively	A dataframe composed by the information present in the gtf and gff files
find_genes_qtls_around_markers	Annotation of genes and QTLs around candidate regions	A data frame composed of the columns present in the input file and the genes or QTLs mapped within or around (if interval provided) the candidate regions
**Data visualization**		
overlapping_among_groups	Overlapping between grouping factors (such as different traits, statistical models, populations, studies, stc.)	A list with three matrices: 1) A matrix with the number of overlapping data; 2) A matrix with the percentage of overlapping; 3) A matrix with the combination of the two previous ones
plot_overlapping	Plot overlapping between data and grouping factors	A heatmap with the overlapping between groups
plot_qtl_info	Plot QTLs information from the gene or QTL annotation output	A pie plot (if QTL class is chosen) or a bar plot (if trait name is chosen) for the annotated QTLs
relationship_plot	Plot the relationship among	A chord plot linking a grouping factor (genomic regions, traits, populations,

	the candidate regions or grouping factors with the annotated genes and QTLs	etc.) with the annotated genes or QTLs
**QTL enrichment**		
qtl_enrich	Performs a QTL enrichment analysis based on a Bootstrap simulation for each QTL class or trait	A data frame composed of the enrichment results for QTL classes or traits present in the input file. 1) QTL: The QTL class or trait used for the enrichment; 2) CHR: The chromosome for that specific QTL or trait (if the option "chromosome" is informed to the argument enrich_type); 3) N_QTLs: Number of observed QTLs or traits in the dataset; 4) N_QTLs_db: Number of each annotatted QTL in the qTL database; 5) Total_annotated_QTLs: Total number of annotatted QTLs; 6) Total_QTLs_db: Total number of QTLs in the QTL database; 7) pvalue: P-value for the enrichment analysis; 8) adj.pval: The adjusted p-value based on the multiple test correction selected by the user; 9) QTL_type= The QTL type for each annotatted trait.
QTLenrich_plot	Creates a bubble plot with the QTL enrichment results	A plot with the QTL enrichment results

Table 2: Top 10 enriched QTLs for the combined analysis performed with the candidate regions from the two studies, Feugang et al. (2009) and Buzanskas et al. (2017), used in the example dataset.

QTL	CHR	# QTLs	# QTLs db	Total # QTLs	Total # QTLs db	p-value	FDR	QTL type
Scrotal circumference	5	132	134	347	5942	1.56E-171	4.98E-169	Reproduction
Scrotal circumference	18	11	13	41	2147	2.20E-18	3.52E-16	Reproduction
Scrotal circumference	9	11	14	30	1395	2.04E-17	2.18E-15	Reproduction
Milk glycosylated kappa-casein percentage	6	71	1607	204	12158	1.86E-15	1.49E-13	Milk
Inhibin level	5	47	285	347	5942	3.38E-11	2.16E-09	Reproduction
Scrotal circumference	21	4	5	12	3606	3.51E-10	1.87E-08	Reproduction
Milk kappa-casein percentage	6	76	2637	204	12158	2.39E-07	1.01E-05	Milk
Triglyceride level	5	6	7	347	5942	2.53E-07	1.01E-05	Health
Milk glycosylated kappa-casein percentage	16	7	44	21	1440	1.29E-06	4.58E-05	Milk
Milk iron content	23	4	8	19	1159	3.48E-06	0.000111329	Milk

Figure legends:

Figure 1: Workflow explaining the main functions implemented on GALLO. The grey rectangles represent the functions, while the rounded and sharp rectangles represent the main goal of that respective function and its input, respectively.

Figure 2: Overlapping between genes (A) and QTLs (B) annotated within the candidate regions (100 Kb downstream and upstream from the significant markers) from Feugang et al. (2009) and Buzanskas et al. (2017). The darker the color within the squares, the higher is the percentage of shared genes or QTLs.

Figure 3: Percentage of QTL type (pie plot) and trait related to Reproduction QTLs (barplots) for the QTL annotation results obtained for Feugang et al. (2009) (A), Buzanskas et al. (2017) (B) and the combined analysis (using both studies) (C).

Figure 4: Bubble plot displaying the enrichment results for the top 10 enrich QTLs identified using the QTLs annotated within the candidate regions from Feugang et al. (2009) and Buzanskas et al. (2017). The darker the red shade in the circles, stronger is the enrichment. The area of the circles is proportional to the number of QTLs. The x-axis shows a richness factor obtained by the ratio of number of QTLs annotated in the candidate regions and the total number of each QTL (and chromosome in the case of this plot) in the reference database.

Figure 5: Chord plot showing the relationship between the top 10 enriched QTLs (Scrotal circumference – SCRCIR, Inhibin level – INHIB, Triglyceride level – TRIGLY, Milk glycosylated kappa-casein percentage – MGKCASP, Milk iron content – MFE, Milk kappa-casein percentage - MKCASP) and the studies (Feugang et al. (2009) in purple and Buzanskas et al. (2017) in pink).

Figure 1

Click here to access/download;Figure;Figure1.png

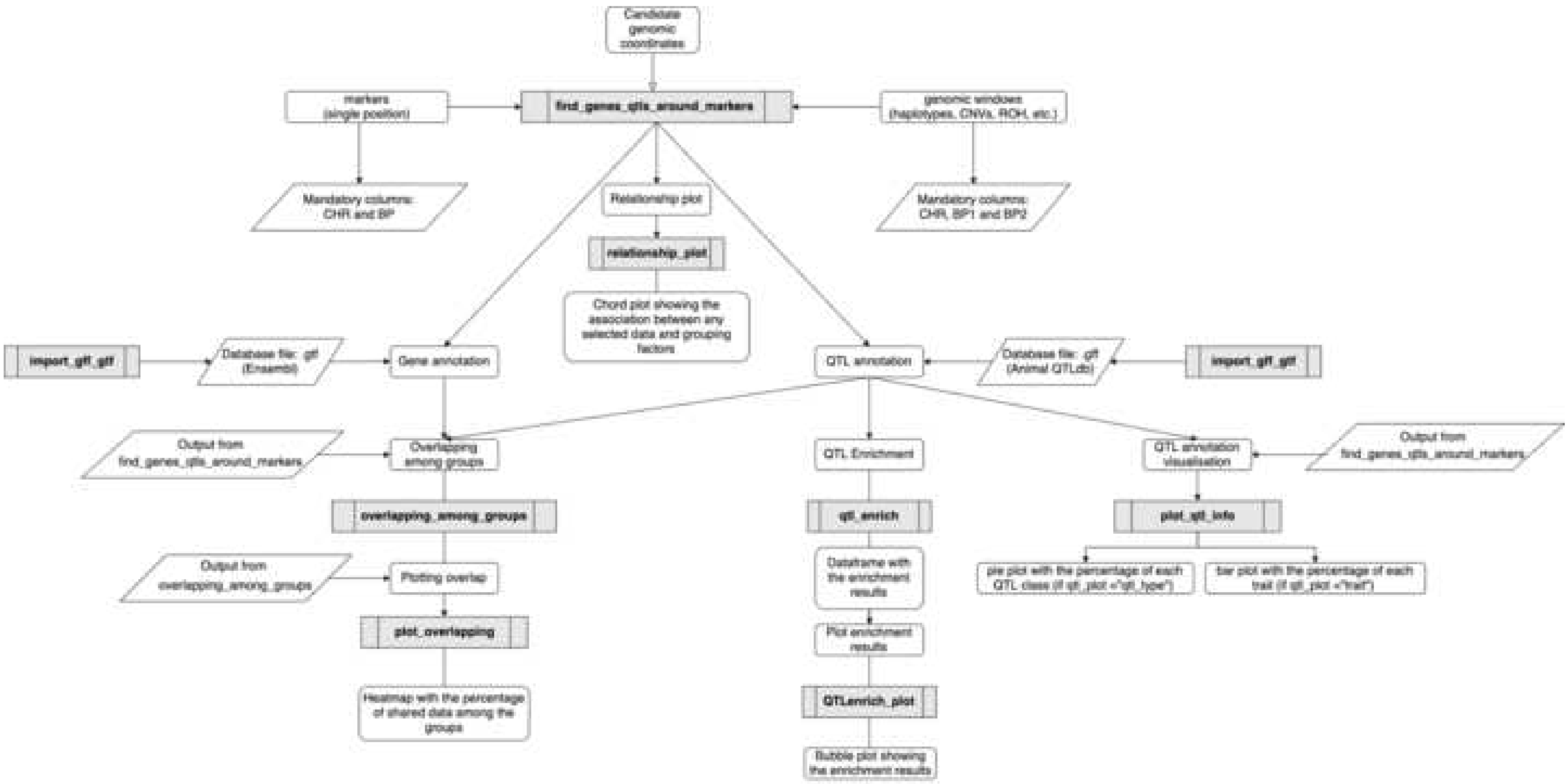

Figure 2

Click here to access/download;Figure;Figure2.png

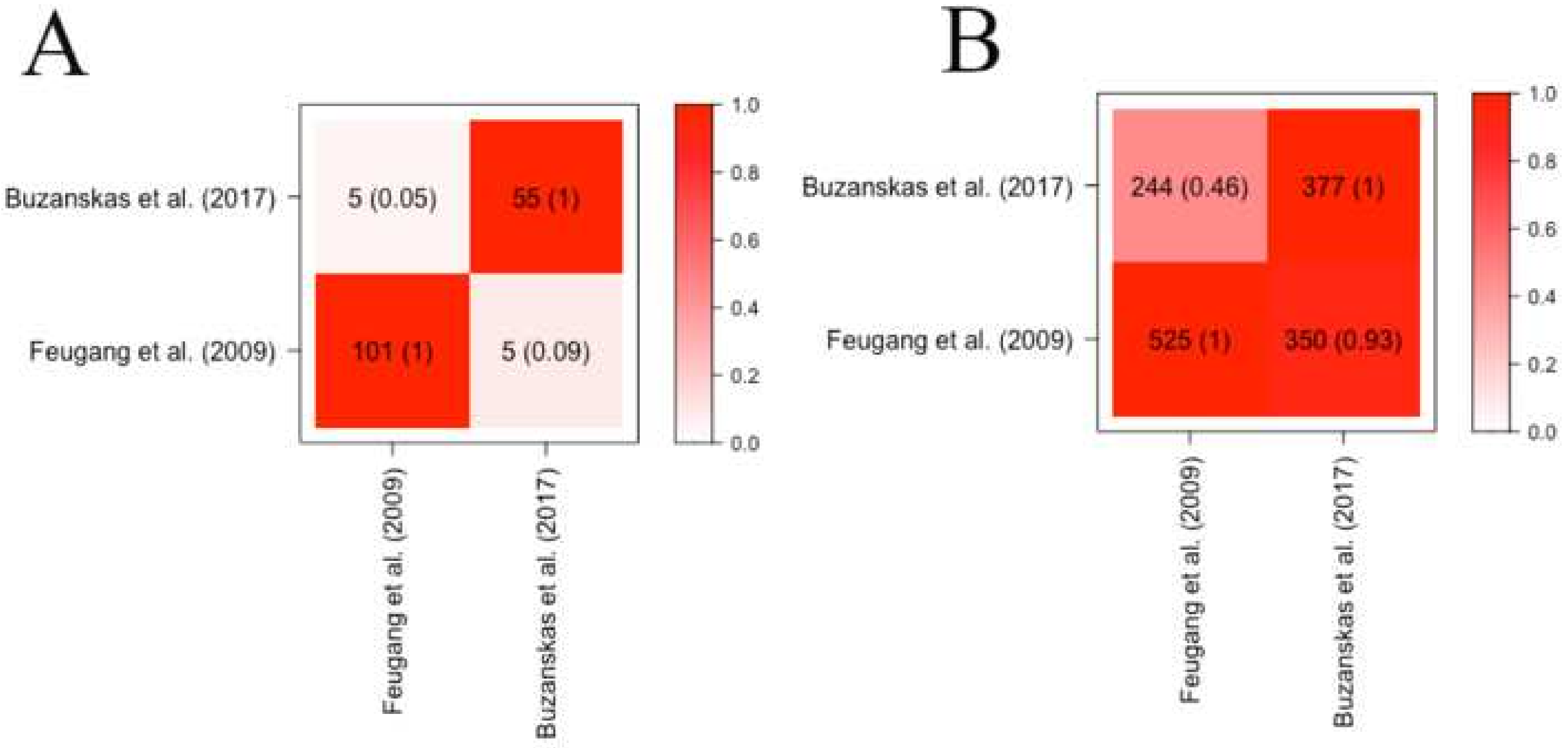

Figure 3

Click here to access/download;Figure;Figure3.png

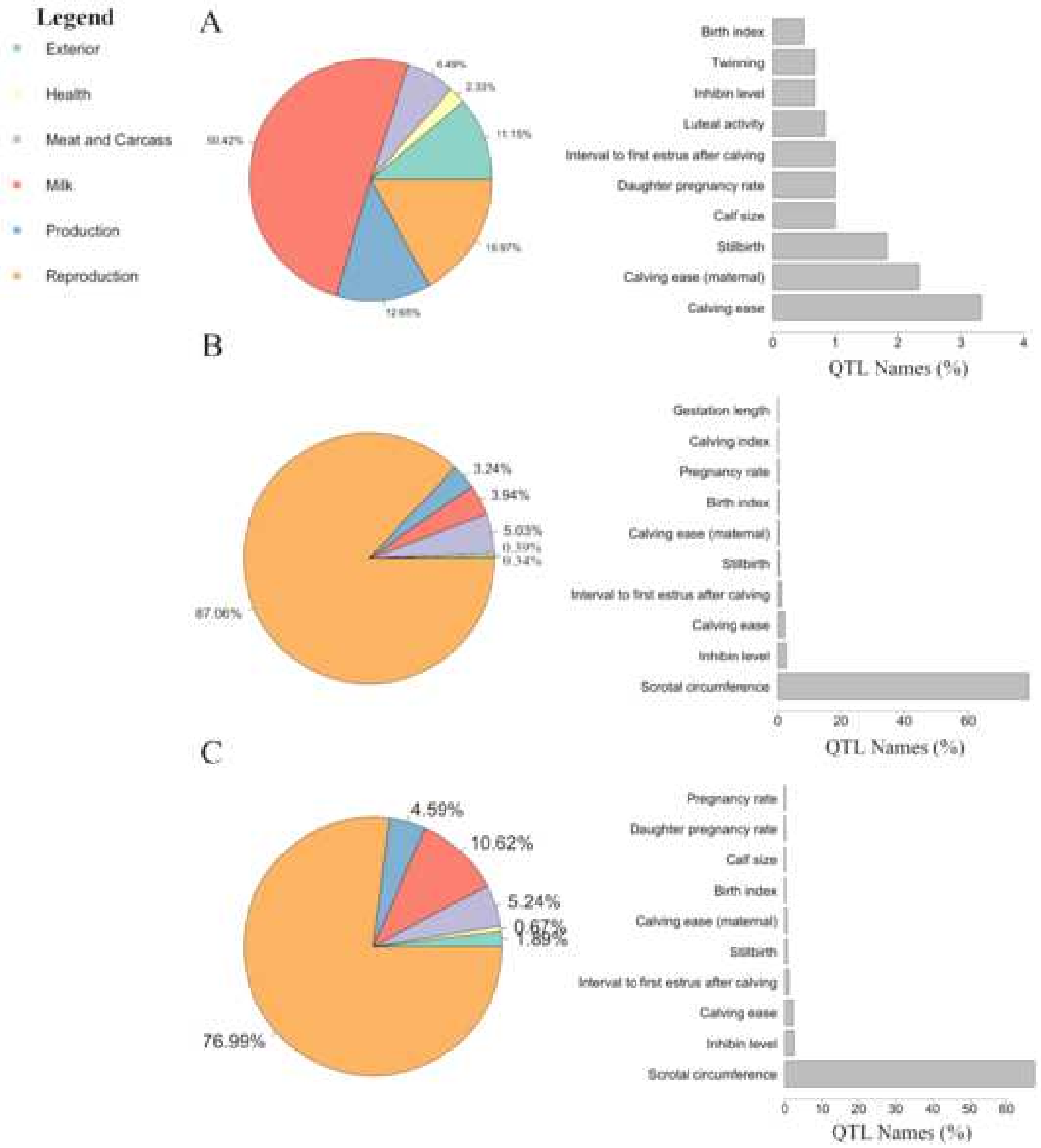

Figure 4

Click here to access/download;Figure;Figure4.png

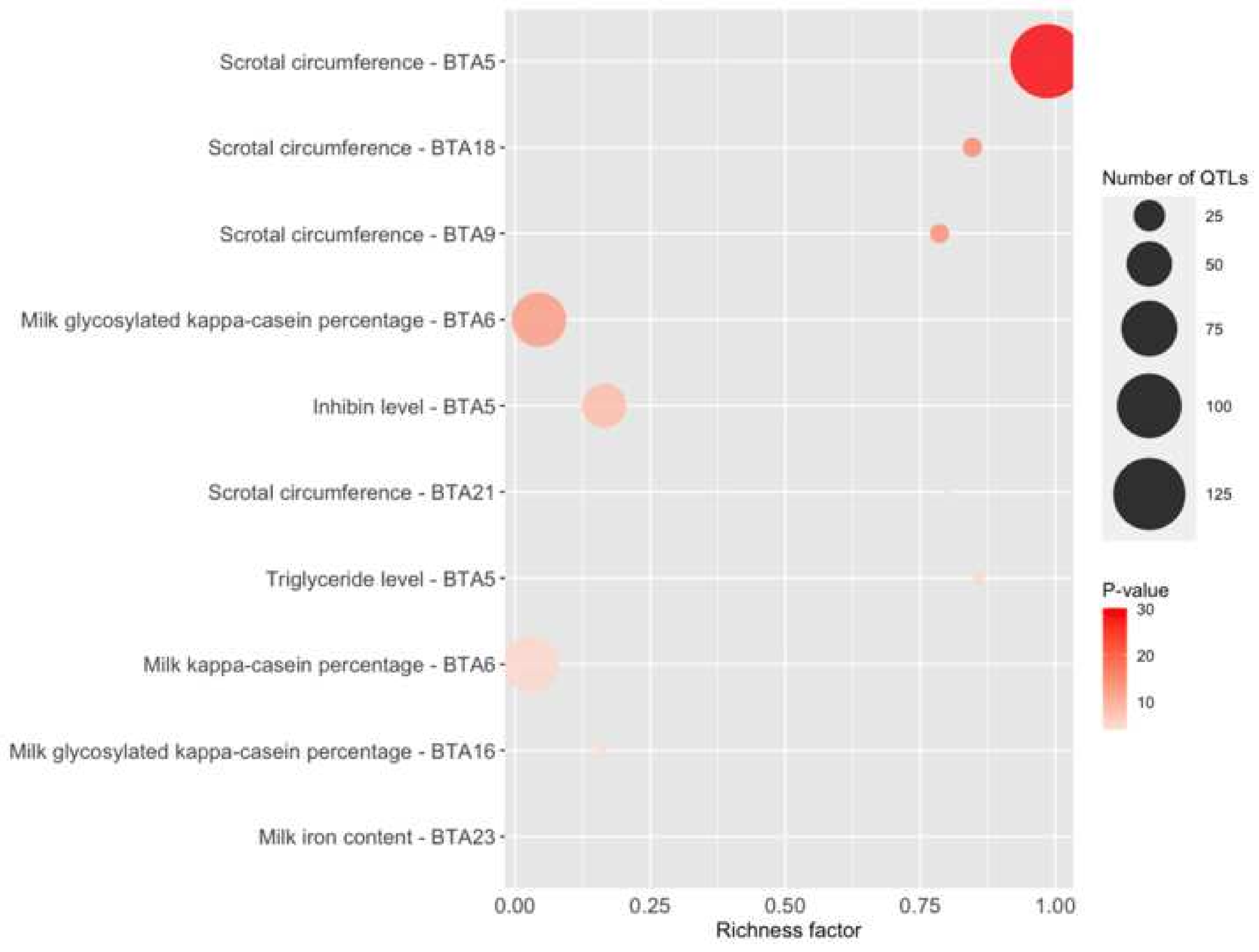

Figure 5

Click here to access/download;Figure;Figure5.png

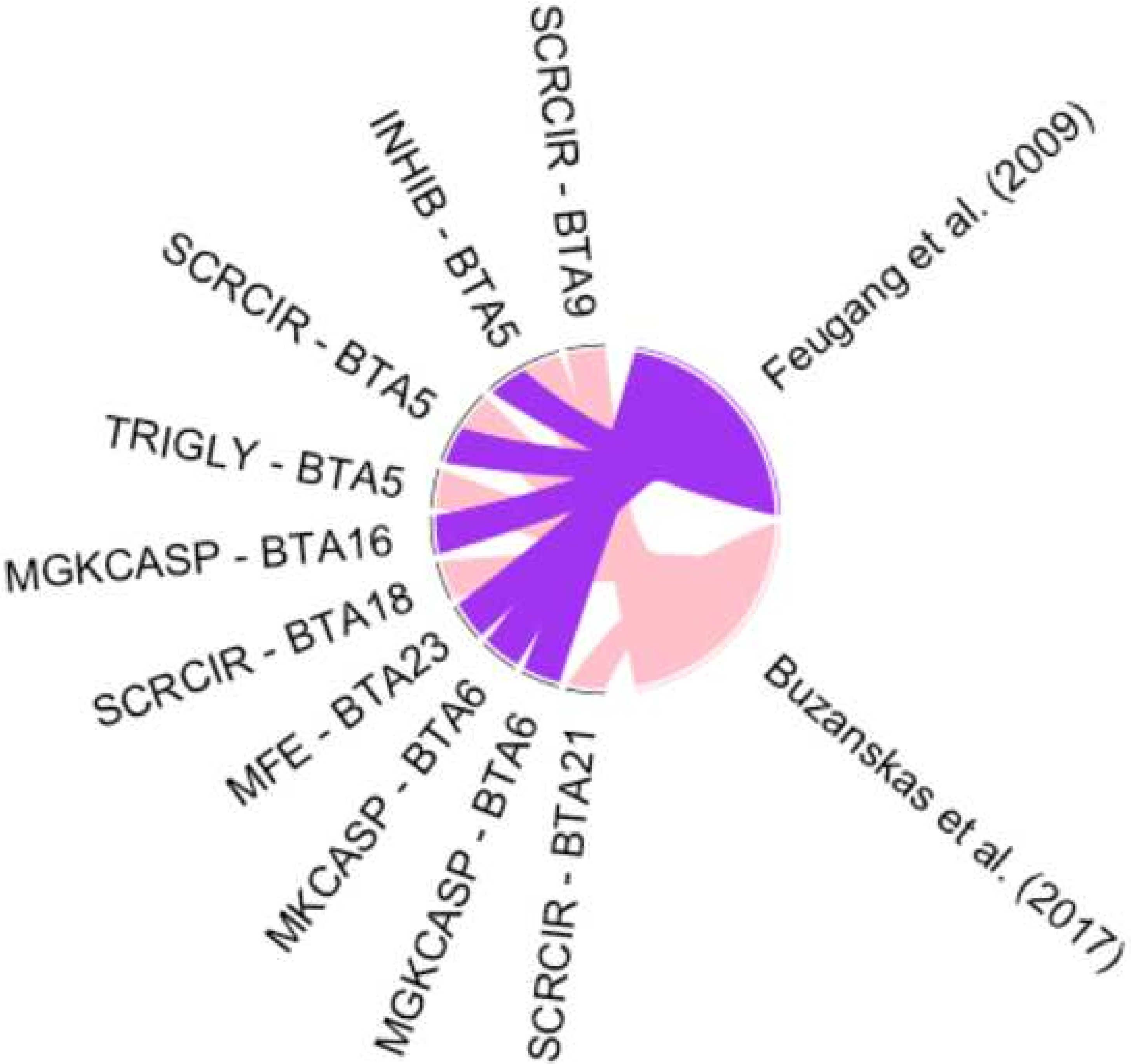

Click here to access/download
Supplementary Material
Supplementary_File1.gtf

Click here to access/download
Supplementary Material
Supplementary_File2.gff.txt

Click here to access/download
Supplementary Material
Supplementary_file3.R

Click here to access/download
Supplementary Material
supplementary_File4.pdf

Click here to access/download
Supplementary Material
Supplementary_Table1.txt

Click here to access/download
Supplementary Material
Supplementary_Table2.txt

Click here to access/download
Supplementary Material
Supplementary_Table3.txt

Click here to access/download
Supplementary Material
Supplementary_Table4.txt

Personal Cover Click here to access/download;Personal Cover;Cover_letter_GALLO_GigaScience_01_09_20_final.doc

Guelph, September 1st, 2020

Dear Editorial Office,

We are pleased to re-submit the manuscript entitled "GALLO: An R package for Genomic Annotation and integration of multiple data source in livestock for positional candidate LOci" for consideration to publish it in the GigaScience. This is a resubmission of this manuscript after the inclusion of all the suggestion and considerations raised by the editor and the prior publication of the package in an official repository, in this case, the CRAN.

The present study introduces the applicability and the functionalities of GALLO package, developed in the R environment.

The identification of quantitative trait loci (QTLs) is a crucial step in the improvement of genomic selection and economic profitability in livestock. The development of high-throughput sequencing and genotyping methodologies and precision livestock farming allowed the identification of thousands of genomic regions associated with several complex traits. Consequently, the number of QTLs identified across the genome in livestock species increased substantially in the last years. Currently, in the Animal QTLdb it is possible to retrieve information about QTLs previously identified in cattle (127,191), chicken (11,340), horse (2,260), pig (29,865), rainbow trout (584) and sheep (3,001). The proper integration of the results obtained from different methodologies and technologies available is a crucial step for the accurate identification of the biological processes regulating the development of complex traits as well as the identification of potential functional candidate genes. However, currently, the integration of multiple data sources is not very straightforward due to limitations in the pipelines and algorithms implemented in the tools available for livestock. Moreover, although the automatization is possible, the direct link between the candidate regions and/or markers with the annotated genes and QTLs is missed. Consequently, this gap is forcing the user to back solve the overlap between the input and output files in order to perform the proper association between the candidate region and/or markers and the annotated genes and/or positional co-localized QTLs. In addition, nowadays there is still a lack of for customized QTL enrichment analyses in the available software and databases. Genomic Annotation in Livestock for positional candidate LOci (GALLO) is an R package, for the accurate annotation of genes and QTLs located in regions identified using the most common genomic analyses performed in livestock, such as Genome-Wide Association Studies and transcriptomics using RNA-Sequencing. Moreover, GALLO allows the graphical visualization of gene and QTL annotation results, data comparison among different grouping factors (e.g., methods, breeds, tissues, statistical models, studies, etc.), and QTL enrichment in different livestock species including cattle, pigs, sheep, chicken, etc. Consequently, GALLO is a useful package for annotation, identification of hidden patterns across datasets, datamining of previous reported associations, as well as the efficient scrutinization of the genetic architecture of complex traits in livestock.

We affirm that this manuscript has not been published elsewhere and is not under consideration by any other journal. All authors have approved the manuscript and agree with its submission to GigaScience.

The authors declare that they have no competing interests. With my best regards,

Angela Cánovas, PhD

Associate Professor Beef Genomics and Small Ruminants
University of Guelph
Department of Animal Biosciences
Centre for Genetics and Improvement of Livestock
Telephone: (519) 824-4129 ext. 56295
email: acanovas@uoguelph.ca
