# Supplementary material for: GALLO: An R package for genomic annotation and integration of multiple data sources in livestock for positional candidate loci
Source: Gigascience. 2020 Dec 30;9(12):giaa149. doi: 10.1093/gigascience/giaa149 (PMC7772745; doi:10.1093/gigascience/giaa149)
Supplement: giaa149_GIGA-D-20-00265_Revision_1 [file giaa149_giga-d-20-00265_revision_1.pdf]

GigaScience

GALLO: An R package for Genomic Annotation and integration of multiple data source in livestock for positional candidate LOci

--Manuscript Draft--

**Manuscript Number:**	GIGA-D-20-00265R1
**Full Title:**	GALLO: An R package for Genomic Annotation and integration of multiple data source in livestock for positional candidate LOci
**Article Type:**	Technical Note
**Funding Information:**	Sustainable Beef and Forage Science Cluster funded by the Canadian Beef Cattle Check-Off, Beef Cattle Research Council, Alberta Beef Producers, Alberta Cattle Feeders' Association, Beef Farmers of Ontario, La Fédération des Productuers de bovins du Québec, and Agriculture and Agri-Food Canada's Canadian Agricultural Partnership (FDE.13.17) — Dr Angela Cánovas
**Abstract:**	The development of high-throughput sequencing and genotyping methodologies and precision livestock farming allowed the identification of thousands of genomic regions associated with several complex traits. The integration of multiple sources of biological information is a crucial step to better understand patterns regulating the development of complex traits. Genomic Annotation in Livestock for positional candidate LOci (GALLO) is an R package, for the accurate annotation of genes and quantitative trati loci (QTLs) located in regions identified in the most common genomic analyses performed in livestock, such as Genome-Wide Association Studies and transcriptomics using RNA-Sequencing. Moreover, GALLO allows the graphical visualization of gene and QTL annotation results, data comparison among different grouping factors (e.g., methods, breeds, tissues, statistical models, studies, etc.), and QTL enrichment in different livestock species including cattle, pigs, sheep, chicken, etc. Consequently, GALLO is a useful package for annotation, identification of hidden patterns across datasets, datamining of previous reported associations, as well as the efficient scrutinization of the genetic architecture of complex traits in livestock.
**Corresponding Author:**	Pablo Augusto de Souza Fonseca<br>University of Guelph<br>Guelph, ON CANADA
**Corresponding Author Secondary Information:**	
**Corresponding Author's Institution:**	University of Guelph
**Corresponding Author's Secondary Institution:**	
**First Author:**	Pablo Augusto de Souza Fonseca
**First Author Secondary Information:**	
**Order of Authors:**	Pablo Augusto de Souza Fonseca
	Aroa Suárez-Vega
	Gabiele Marras
	Angela Cánovas
**Order of Authors Secondary Information:**	
**Response to Reviewers:**	Reviewer reports:<br><br>Editor comments<br><br>Dear Dr. Fonseca,

Powered by Editorial Manager® and ProduXion Manager® from Aries Systems Corporation

Your manuscript "GALLO: An R package for Genomic Annotation and integration of multiple data source in livestock for positional candidate LOci" (GIGA-D-20-00265) has been assessed by four reviewers. Based on these reports, I am pleased to inform you that it is potentially acceptable for publication in GigaScience, once you have carried out some essential revisions suggested by our reviewers.

While the overall impression is positive, there are a couple of issues mentioned in the reviewers' reports that require attention during the revision.

I'd like to highlight a few of the points that seem particularly important to me:

- Reviewer 1 points out that a more detailed explanation is required to justify the need for this tool, compared to the available competitors. The reviewer also run into problems when trying to test the tool, this should be fixed.

Answer: In this current version of the manuscript a more detailed explanation regarding the advantages to use GALLO compared with the available tools was provided. Additionally, supplementary file 4 was removed in this version of the manuscript and the examples are available in the package vignette, which was properly cited in the manuscript. In this new version of the vignette the errors were fixed. All the changes in the manuscript are highlighted in this new version.

- Reviewer 3 is a Biometrician and QTL expert, but not working in the livestock field. In principle I agree with his comments that the tool would be of wider interest if it could be applied outside the livestock field. I am aware this may not be your intention, but as the code is open source, you may discuss this point and maybe give some pointers or examples in the manuscript as to how others could build upon this work, to apply the code to problems in other fields.

Answer: Thank you for the comment. We included a discussion about the use of GALLO for other species than livestock. We agree that the package could have a wider interest if the data obtained from other species could be used. This information is available on Lines 298-302.

I agree with reviewer 3 that there should be an easy way to test the code with the data from the paper (e.g. by including a working example with data in github, or you may also consider to provide a computational capsule with code and data in CodeOcean https://codeocean.com/)

Answer: In this current version we provided the proper citation for the package vignette, which contains a series of examples using data that is internally available after the package installation (line 321).
Regarding the reviewer's recommendation to use Bioconductor instead of CRAN: From the journal's perspective, this is your decision - R tools presented in GigaScience should be submitted to either CRAN and/or Bioconductor, which platform is selected is the author's choice.

Answer: Thank you for the comment. We addressed the comment from reviewer 3 highlighting the fact that CRAN is the main R repository and all the packages available on CRAN can be used along Bioconductor packages without any problem.

- Reviewer 4 found some technical issues with the R package that I hope you can fix.

Answer: We performed the code edits and fixed the errors listed by the reviewer 4. The package was already updated to CRAN with a new version containing these edits.

Reviewer #1: Fonseca et al. - GALLO: An R package for Genomic Annotation and integration of multiple data source in livestock for potential candidate LOci

Description of useful R package for livestock studies to find overlap between important genomic regions from own results with other studies/public databases and capture it in a visual way, with example based on datasets from 2 GWAS studies on cattle fertility.

Although the paper reads well, some improvement of the English is needed. It is mainly the use of the right tense and plural form, see line-by-line comments below, so please pay attention to that. The sections do not follow a traditional paper setup, which is understandable for the publication of an R package. However the section named Methods also includes Results. Not sure what the journal policy of GigaScience is for paper like this.

Answer: Thank you for the comment. The current version of the manuscript was reviewed by an English native speaker. The sections were restructured in this current version in order to be more clear. All the changes are highlighted in yellow.

The authors indicated that the R package is similar to BiomaRt, and gave performance differences in term of execution time of comparable commands. BiomaRt is a renowned package and was faster. It would be nice if the authors can indicate what benefits GALLO has over BiomaRt. Why was this package needed (e.g. what did you miss in biomaRt)?
Also it may be worthwhile to explicitly indicate why R is the appropriate language for this package. There are thing mentioned scattered over the paper, e.g. like visuals and no need for intermediate output files, please summarize them somewhere.

Answer: Thank you for the comment. The comparison between GALLO and other available tools is better discussed on lines 241-253 and 468-476 of the revised version of the manuscript.

The authors indicated that the matrices showing QTL overlaps were not symmetrical. An explanation for that should be given. Also why many QTLs were overlapping, but only 5 genes. Explaining this will help a user understand what the package does in the background.

Answer: The explanation about the not symmetrical nature of the percentage matrix obtained in GALLO is better explained on lines 167-172. Briefly, this matrix is not symmetrical because GALLO calculates the percentage of records shared as a function of the total number of records for each group. For example, groups A and B shared 5 records, where group
A has 10 records in total and group B has 5 records. Consequently, the percentage of shared records in A is 50% while the percentage of shared genes in B is 100%.
Additionally, we provided a better explanation about the QTL annotation. The number of QTLs annotated in a genomic window tend to be substantially larger than the number of genes. This is due to the number of records present. While there are ~20K genes annotated in the bovine genome, the Animal QTLdb has ~160K QTL records spread across the genome. Additionally, a QTL for the same trait, example milk yield, could be annotated in the same loci, but with slightly different windows for different breeds of the same species. This means that although the underlying QTL could be the same, there are different mutations acting in a similar way in the same gene, therefore the record will be different in the QTL database.

I tried to run the code in Supplementary file 4, but was not successful. I struggled loading the gtf and gff files correctly. Below you can find the error I ran into. I guess the file was not loaded as a gtf/gff file, but just as a table. I later tried the published vignette, and there it worked fine following the code provided to load gtf/gff files.

After downloading the gtf file from ensemble following the link and unzipping it, the following command did not work.

```
> out.genes<-find_genes_qtls_around_markers(db_file="Bos_taurus.UMD3.1.94.gtf",
+ marker_file=QTLmarkers, method = "gene",
+ marker = "snp", interval = 500000, nThreads = NULL)
You are using the method: gene with snp
Error in { : task 1 failed - "$ operator is invalid for atomic vectors"
```

The downloaded file looked like this:

```
head -n6 Bos_taurus.UMD3.1.94.gtf
#!genome-build UMD3.1
#!genome-version UMD3.1
```

#!genome-date 2009-11
#!genome-build-accession NCBI:GCA_000003055.3
#!genebuild-last-updated 2011-09
1 ensembl gene 19774 19899 . - . gene_id "ENSBTAG00000046619"; gene_version "1"; gene_name "RF00001"; gene_source "ensembl"; gene_biotype "rRNA";

Answer: Thank you very much for your comment. This issue was caused due to an outdated version of supplementary file 4. The submission of the package to CRAN required some changes in the code structure. Mainly regarding the gff and gtf importing process. The code was updated in the revised version of the manuscript. In order to avoid future problems, the supplementary file 4 was removed from the current version of the manuscript and the link for the updated version of GALLO vignette was provided.

Line-by-line comments:
Title Change 'source' to 'sources', and write 'livestock' with capital for the acronym GALLO

Answer: Done.

L15-16 Why precision livestock farming? I associate that with phenotyping using sensors. Remove?

Answer: Thank you for your comment. We decided to remove the term precision livestock farming from the current version of the manuscript.

L38-40 Although the statement about PLF is fine, I find it not so relevant for this manuscript and even a bit distracting

Answer: The sentence was removed in this current version of the manuscript.
L44 Remove 'new' (its relative)
Answer: Done.

L51 Remove 'the development of'

Answer: Done.

L82 Change 'wrote' into 'written'

Answer: Done.

L86-87 Please rephrase the ending of this sentence. Not proper English.

Answer: Done.

L90-91 Is it really the RNA-sequence data & whole genome sequence data (i.e. reads) that can be integrated or is it the called (structural)variants? As I understand from figure one, it is not reads that are supplied, but rather variants. So make sure to be explicit about this.

Answer: Done.

L113 Change 'present' into 'presented'

Answer: Done.

L153 Change 'order' into 'other

Answer: Done.

L166 Change 'can be used compare' into 'can be used to compare'

Answer: Done.

Powered by Editorial Manager® and ProduXion Manager® from Aries Systems Corporation

L169 Change second 'overlapping' into 'overlap'

Answer: Done.

L170 Change 'gene' into 'genes'

Answer: Done.

L172 How come the matrices are not symmetrical with respect to number over overlapping QTL? Are there multiple regions from one study overlapping with only one region in the other? I assume the matrix is always symmetrical for overlapping genes?

Answer: Briefly, this matrix is not symmetrical because GALLO calculates the percentage of records shared as a function of the total number of records for each group. For example, groups A and B shared 5 records, where group A has 10 records in total and group B has 5 records. Consequently, the percentage of shared records in A is 50% while the percentage of shared genes in B is 100%. Therefore, in both the gene and QTL data, the percentage matrix can be not symmetrical. A more detailed explanation was presented in the previous comment.

L180-183 Were the genes identified based on the QTL positions? If that is the case, it seems that 5 genes overlapping is rather low with so many QTL overlaps. It would be good to explain what is the reason. I can imagine that QTL in intergenic regions are present, or that QTL regions have only short overlaps not including the genes.

Answer: The genes were identified based on the genomic coordinates of the candidate markers associated with the phenotypes evaluated by Buzanskas et al. (2017) and Feugang et al. (2009). Regarding the number of QTLs and genes annotated in the same genomic regions, the number of QTLs annotated in a genomic window tend to be substantially larger than the number of genes. This is due to the number of records present. While there are ~20K genes annotated in the bovine genome, the Animal QTLdb has ~160K QTL records spread across the genome.

L182-183 I don't understand what you mean here. There are no overlapping genes so why would there be related biological processes?

Answer: Thank you for the comment. This sentence was removed in the current version of the manuscript.

L190 Please define what is meant with QTL types

Answer: The QTL types available for cattle were defined in this current version of the manuscript.

L239 Change 'can used the gene' into 'can be used for the gene'

Answer: Done.

L241 Change 'or' into 'to'

Answer: Done.

L255 Complex what?

Answer: Thank you for the comment. In this current version of the manuscript we included the sentence “complex biological mechanisms”.

L279 Change 'find' into 'found'

Answer: Done.

L281-282 Please rephrase this sentence, not proper English

Powered by Editorial Manager® and ProduXion Manager® from Aries Systems Corporation

Answer: Done.

L307 Change 'find' into 'found'

Answer: Done.

L405-407 Reference 27 is a duplicate of reference 10, please correct

Answer: Done.

L435 Change 'overlapping' into 'overlap'

Answer: Done.

L444 The darker red the more significant, not?

Answer: Done.

Figure 4 P-value scale looks like -log10(p-value)

Answer: Thank you for the comment. Indeed, it is -log10(p-value) scale. This was corrected in the current version of the manuscript.

Reviewer #2: I think the GALLO package is useful to scientists specialized in overall genome analyses. Although some of the functions in GALLO can be found in other softwares such as bedtools, the idea of QTL enrichment analysis is highly useful. It is also good to combine all of these tools into one package to further help researchers in conducting the required tasks.
Two issues I would like to raise to further improve the package:

1- I do recommend to include a function that allow for gene enrichment analysis that complement the qtl enrichment analysis.

Answer: Thank you for your suggestion. We are open to the inclusion of new useful functions for GALLO. In this specific case, the development of a gene enrichment analysis is not a simple task as there are fundamental limitations regarding the number of observations for the gene. Using a hypergeometric test as an example (which is the test used for QTL enrichment analysis in GALLO), the number of traits annotated within the candidate regions is compared with the total number of the trait of interest in the QTL database (genome-wide or chromosome-wide, depending of the user choice). In the case of genes, the total number of a gene in the database (the gtf file) will not always be one. On the other hand, the use of functions for the enrichment of gene families, gene ontology terms, and metabolic pathways associated with the positional candidate genes is very useful. However, there are several tools currently available which provide a very accurate and complete toolset of functions for this kind of enrichment. Therefore, we strongly recommend the users to integrate the results obtained on GALLO with other packages in R which can perform this kind of enrichment.

2- Further explanation is required for the hypergeometric test approach to further understand how the QTL enrichment analysis is performed.

Answer: Thank you for your comment. We provided more information regarding the hypergeometric test in this current version of the manuscript (Lines 213-217).

Reviewer #3: This article discusses a newly developed R package GALLO that allows users to quickly annotate quantitative trait loci (QTLs) or genes obtained from genome wide association studies of livestock traits. The package focusses on providing a simple method for linking QTLs/genes to candidate regions in downloadable livestock genomic databases. The package also provides functionality for post-processing of the

Powered by Editorial Manager® and ProduXion Manager® from Aries Systems Corporation

results through graphical representations and QTL enrichment analyses.

Its clear this package does fill a need for users working specifically in genome wide association research of livestock traits. However, I have outlined some issues associated with the article/package and its possible alignment with the journal aims and scope.

This is quite a simple package. Its main task is matching and returning overlapping content between two data frames in R where one of the data frame has a potentially large number of rows associated with it. In my opinion, this innate package simplicity reduces the strength of the article/package and its alignment with publication in GigaScience.

Answer: The functions available on GALLO comprise a much more diverse group of tasks than just the simple matching and overlapping between data frames. As stated in the manuscript:

“Currently, there are several tools that implement functions for gene (i.e., Biomart and BEDTools) and QTL annotation (Animal QTLdb). However, these tools have limitations regarding the automatization process to analyze results from multiple candidate regions (Biomart web application and the R package and Animal QTLdb) or for the visualization of the results. Moreover, although the automatization is possible, the direct link between the candidate regions and/or markers with the annotated genes and QTLs is missed. Consequently, this gap is forcing the user to back solve the overlap between the input and output files in order to perform the proper association between the candidate region and/or markers and the annotated genes and/or positional co-localized QTLs.”

In addition to the advantages provided by the annotation function of GALLO mentioned above, GALLO provides the user a set of functions for graphical visualization and comparison of the results obtained by multiple studies, statistical models, populations, etc. It is important to highlight that currently there is no software, package, or function available for QTL enrichment using the information available in the Animal QTLdb, the most complete and reliable database for QTLs identified in livestock species. GALLO is the first package to provide this function and allow the user to perform the enrichment using a genome-wide and chromosome-wide approach, in addition to a QTL type or trait selection. This kind of function is extremely useful due to the bias of investigation of several traits in livestock species, such as milk production traits. Additionally, the option for chromosome-wide analysis helps to adjust for the effects of specialized regions in the genome, such as chromosome 29 for meat quality traits in cattle and chromosome 14 for lipid content in milk (and milk production in general).

Taken together, these functionalities of GALLO are a unique set of tools for data integration, annotation and comparison in association studies with a strong emphasis on livestock species.

The functionality has been written specifically for livestock genetics. Why can't this be more general and provide functionality for a other related biological organisms such as heavily researched crops like wheat, maize or barley? I understand this may not be the authors intent but the narrow scope of the package lessens its potential for publication in a quality journal such as GigaScience.

Answer: The functions available on GALLO can be used for any other species. The main reason we reinforce the livestock application is the use of the Animal QTLdb information for QTL annotation. Once the user uses a similar format for QTL annotation for any other species, the functions of GALLO will behave exactly the same as the livestock species available on Animal QTLdb. We acknowledge this comment in the revised version of the manuscript and have included a sentence highlighting the applicability to other species (Lines 298-302).

From a visibility perspective it feels like it would be more natural for this package to be in the Bioconductor repository so it could potentially link with overarching gene annotation packages such as AnnotationData.

Powered by Editorial Manager® and ProduXion Manager® from Aries Systems Corporation

Answer: The package is currently accepted and available on CRAN, which is the main repository for R packages. Despite the specialization of Bioconductor for packages related with "biological analysis" CRAN also has a high visibility and deposited packages can be easily linked with packages available on other repositories, such as Bioconductor.

The software package is a very recent submission to CRAN. From past experience, the publication of packaged code that has been recently created can be problematic. Immature code has the potential to require many more dramatic amendments, additions and bug fixes.

Answer: The package is already accepted and published on CRAN. All edits to the code suggested by automatic and manual checking were already provided and accepted by the CRAN team. As any package, GALLO is under constant code evaluation and updating. For the moment, any major bug has been reported. However, as soon as these problems are identified they will be fixed, and the package will be updated on CRAN. The package was already used for several research groups which resulted in several manuscripts currently published, accepted or under development. Some examples are shown below:

Lam, S., et al. Development and comparison of RNA-Sequencing pipelines for more accurate SNP identification: Practical example of functional SNP detection associated with feed efficiency in Nellore beef cattle. BMC Genomics 21: 703 (2020). https://doi.org/10.1186/s12864-020-07107-7

Lam. S., et al. Identification of functional candidate variants (SNPs and INDELs) and genes for feed efficiency in Holstein and Jersey cattle breeds using RNA-Sequencing. Journal of Dairy Science. 2020. In press.

Sweett, H., et al. Genome-wide association study to identify genomic regions and positional candidate genes associated with male fertility in beef cattle. Accepted for publication in Scientific Reports.

I would have liked the ability to immediately test the code with the data sets that are mentioned in the Method section of the paper. However, the submitted R script does not contain code that matches the code mentioned in the manuscript. In fact, the script contains path names from the authors local computer.

Answer: The R script submitted as supplementary material was edited in order to provide a more detailed step by step analysis of the code and data provided. It is important to highlight that the package also has a vignette which comprises a different dataset with a complete explanation of each function and output.

The title of the paper has been expanded from the title of the R package. Im not sure there is good justification for this and I am immediately concerned about the spelling error in the title for the article. It should be the plural ``sources''.

Answer: The manuscript is an introduction to the R package. Therefore, we choose to include the complete name of the package in order to provide an easier way for the users to identify the manuscript associated with the package. Regarding the typo on the title, the error was fixed in the revised version of the manuscript.

Following from this previous point, although the paper is quite well written, it needs a pre-submission editor with english as their first language to proofread the main document text. This would create a more succinct manuscript through removal of repeated content and more general punctuation issues.

Answer: Thank you for the comment. The current version of the manuscript was reviewed by an English native speaker.

Reviewer #4: Overall the manuscript is well written, easy and logical to follow and also presents an interesting addition to the toolbox of genomic data analysis with R. Despite the fact, that the manuscript makes an overall good impression to me, I have a few comments that I would like the authors to address. In detail these are

Powered by Editorial Manager® and ProduXion Manager® from Aries Systems Corporation

Specific R-package comments

1. Please check the styling of the code chunks in the manual (e.g. spacing, linebreaks, etc.)

Answer: Thank you for the comment. We reviewed all the code styles for both manual and vignette present on GALLO. The package is currently accepted and updated on CRAN as well.

2. import_gff_gtf(): I think the function could estimate the filetype from the filename (strsplit -> ifelse) so that this parameter could be optional.

Answer: Thank you for your suggestion. We decided to let the user inform the file extension due to potential problems with the names of the gtf and gff files when downloaded from the respective databases. For example, the gff files from Animal QTLdb are constantly renamed as ".gff.txt" after the decompress process.

3. find_genes_qtls_around_markers(): Please add also a match.arg for the `marker` input

Answer: Thank you for the suggestion. The match.arg was included in the find_genes_qtls_around_markers() function.

4. Instead of referring to the table() command in line 142 (actually, I am not sure how to get the number of genes with it), I would recommend to create S3 classes for important return objects and then create own summary(), print() and possibly even plot() functions for it.

Answer: Thank you for your comment. Assuming the gene or qtl annotation results were saved in a data.frame called out.results, the number of genes and QTLs can be easily retrieved with the following commands, table(out.results$gene_name) and table(out.results$traits), respectively. New functions for plots and summary statistics are currently under development for GALLO and will be available in the next update.

5. QTLenrich_plot(): In the vignette, the scale for the p-value goes up to 100. If you use the label 'P-value', please keep it between 0 and 1, or change the label name. Also, I am not sure about the colors, in the example of the vignette, the 'P-value' with 100 is red, whereas smaller p-values are white (in contrast to what is written in the Figure3 caption). So, currently the description and the labels do not match. Further, although white coloured bubbles are less informative and maybe this is a problem with my screen, but from the figure I hardly could see any bubbles (besides the red ones...), maybe you could slightly adjust the colours or the background?

Answer: Thank you for your comment. The label was changed in the revised version of the manuscript and vignette. The correct scale is -log10(p-value). The description of the figure was corrected as well. Regarding the background of the plot, thank you very much for the suggestion. The plot will be updated in order to provide a light grey background, which will make the small white dots easier to see.

How do you handle the situation, when a large dark bubble is covering a smaller (dark) bubble, would the user see that or would that be hidden? Maybe using a frame and then plotting from large to small could solve this?

Answer: Thank you for your comment. The QTLenrich_plot() function allows the user to freely decide the order of plots for the enrichment results. Therefore, if an overlap is observed between two or more records, the user can rearrange the order of the plots and avoid this problem.

6. Something is odd with your parallel code. When I run the code below, the runtime is getting longer with more cores I use:

```
> system.time(out.genes<-find_genes_qtls_around_markers(db_file=gtfGenes,
+                                        marker_file=QTLmarkers[rep(1:141,500),], method =
"gene",
```

```
+                                        marker = "snp", interval = 500000, nThreads = 2))
You are using the method: gene with snp
   user  system elapsed
   0.81    0.28    5.45

> system.time(out.genes<-find_genes_qtls_around_markers(db_file=gtfGenes,
+                                        marker_file=QTLmarkers[rep(1:141,500),], method =
"gene",
+                                        marker = "snp", interval = 500000, nThreads = 4))
You are using the method: gene with snp
   user  system elapsed
   0.87    0.32    6.30

> system.time(out.genes<-find_genes_qtls_around_markers(db_file=gtfGenes,
+                                        marker_file=QTLmarkers[rep(1:141,500),], method =
"gene",
+                                        marker = "snp", interval = 500000, nThreads = NULL))
You are using the method: gene with snp
   user  system elapsed
   0.87    0.24    1.77
```

The same is true for all other functions I tried that have a nThread option. Whenever I choose NULL, it is faster than 2 or 4...

Further, I would prefer that the parallel functions accept nThreads=1 as valid input.

Answer: Thank you for your comment. The issue regarding the parallel code seems to be solved in the current version of the package, which is accepted and updated on CRAN. Additionally, we edited the code to allow nThreads=1 as a valid input. In Figure 1 of this review, we show a boxplot representing the distribution of the elapsed time for the qtl annotation using 3 options of nThreads: 2, 4, and NULL after 100 iterations. It is important to highlight that the NULL option result in the use of all available cores in the machine.

Figure 1: Violin plot showing the distribution of elapsed time (seconds) for three options of nThreads argument of find_genes_qtls_around_markers() function after 100 iterations. In red, green and blue, two, four and all available cores were chosen, respectively.

7. plot_qtl_info() really easily creates an error that the figure margins are too large. Please catch this better. Also, I think you require many graphical parameters from the user to enter, what makes the use of the plotting functions kind of cumbersome. I think you could add functions that estimate the best fitting values for the user as default. Especially that the user needs to change the par() settings shouldn't happen often.

Answer: Thank you for your comment. The issue with the margins seems to be caused by the position of the legend in the pie plot. We introduced a new argument allowing the user to define the legend position (horizontal or vertical). Regarding the number of arguments, the majority of the graphical arguments can work with the default options, as well as any other plot. However, due to the complexity of the plot schemes and the number of available records, additional arguments were necessary in order to provide a better visualization scheme for the user.

8. In the vignette 0.3.3.2 it should say dev.off() instead of dev.off

Answer: Done.

9. In QTLenrich_plot() there are smaller bubbles than mentioned in the legend. Please add also the small ones to the legend

Answer: Done.

10. There are still few notes and warnings in the cran check, that probably easily can

	be resolved. I think that should be done.<br><br>Answer: All notes and warnings were related to minor issues such as the size of the data folder and the new submission email and ID of the maintainer. These issues are fixed.<br><br><br>Minor comments:<br><br>l.1: I suppose 'livestock' should be capitalized also in the title to get the abbreviation GALLO?<br><br>Answer: Done.<br><br>l.47: Please add an date when you checked those numbers from animal QTLdb, when I checked they appear larger<br><br>Answer: Thank you for the comment. It is fixed in the current version of the manuscript<br><br>l.70: The 'functional' you do not have in other descriptions of the name, maybe it would be nice to be consistent<br><br>Answer: Done.<br><br>l.139: (and others): Please format code snippets consistent (data(...)) e.g. with monospace or italic, as you did. Further, I would prefer to use quotions rather than variable names in the data calls (like data("QTLwindow"))<br><br>Answer: Thank you for the comment. We applied the same format for all the code snippets across the manuscript.<br><br>l.145: Though hardly noticable by the user, I wouldn't say that the performances are similar between the compared tools. Biomart seems to be faster by factor 22 and BEDtools by factor 7. Maybe you could rephrase it?<br><br>Answer: Thank you for your comment. We removed the sentence where the similarity between the efficiency of the software was compared. Additionally, on lines 151-152 we included the following sentence: “Consequently, GALLO obtains a more elaborate and informative output without substantially compromising the computational demand of the analysis”.<br><br>General comments:<br>1. Maybe it is a matter of taste or formatting guidelines, but I would prefer seeing code snippets written in a monospace rather then using italics.<br><br>Answer: Thank you for your comment. We think that the italic is a good way to highlight the codes in the manuscript. Additionally, we are following the writing style of previous R packages publications available on GigaScience.<br><br>2. Please check that code snippets are consistent formatted throughout the manuscript<br><br>Answer: Done.
**Additional Information:**	
**Question**	**Response**
Are you submitting this manuscript to a special series or article collection?	No
**Experimental design and statistics**<br><br><br>Full details of the experimental design and	Yes

Powered by Editorial Manager® and ProduXion Manager® from Aries Systems Corporation

statistical methods used should be given in the Methods section, as detailed in our Minimum Standards Reporting Checklist. Information essential to interpreting the data presented should be made available in the figure legends.<br><br>Have you included all the information requested in your manuscript?	
**Resources**<br><br>A description of all resources used, including antibodies, cell lines, animals and software tools, with enough information to allow them to be uniquely identified, should be included in the Methods section. Authors are strongly encouraged to cite Research Resource Identifiers (RRIDs) for antibodies, model organisms and tools, where possible.<br><br>Have you included the information requested as detailed in our Minimum Standards Reporting Checklist?	Yes
**Availability of data and materials**<br><br>All datasets and code on which the conclusions of the paper rely must be either included in your submission or deposited in publicly available repositories (where available and ethically appropriate), referencing such data using a unique identifier in the references and in the "Availability of Data and Materials" section of your manuscript.<br><br>Have you have met the above requirement as detailed in our Minimum Standards Reporting Checklist?	Yes

Manuscript

Click here to access/download;Manuscript;manuscript_Gigascience_26_10_2

GALLO: An R package for Genomic Annotation and integration of multiple data sources in Livestock for positional candidate LOci

Pablo A.S. Fonseca[1*], Aroa Suárez-Vega[1], Gabriele Marras[1,2], and Ángela Cánovas[1*]

[1]University of Guelph, Department of Animal Biosciences, Centre for Genetic Improvement of Livestock, Guelph, N1G 2W1, Ontario, Canada.

[2]The Semex Alliance, Guelph N1G 3Z2, Ontario, Canada

Contact:

PASF: pfonseca@uoguelph.ca

ASV: asuarezv@uoguelph.ca

GM: gmarras@uoguelph.ca

AC: acanovas@uoguelph.ca

[*] Corresponding author

Abstract

The development of high-throughput sequencing and genotyping methodologies allowed the identification of thousands of genomic regions associated with several complex traits. The integration of multiple sources of biological information is a crucial step required to better understand patterns regulating the development of these traits. Genomic Annotation in Livestock for positional candidate LOci (GALLO) is an R package developed for the accurate annotation of genes and quantitative trait loci (QTLs) located in regions identified in common genomic analyses performed in livestock, such as Genome-Wide Association Studies and transcriptomics using RNA-Sequencing. Moreover, GALLO allows the graphical visualization of gene and QTL annotation results, data comparison among different grouping factors (e.g., methods, breeds, tissues, statistical models, studies, etc.), and QTL enrichment in different livestock species including cattle, pigs, sheep, and chickens, etc. Consequently, GALLO is a useful package for the annotation, identification of hidden patterns across datasets, datamining previously reported associations, as well as the efficient scrutinization of the genetic architecture of complex traits in livestock.

Keywords: Multi-omics integration; QTL annotation; Gene annotation; Datamining; QTL enrichment analysis; Livestock

Background

The identification of quantitative trait loci (QTLs), genomic regions linked to complex traits through association tests using genetic markers and phenotypic traits, is a crucial step in the improvement of genomic selection and economic profitability in livestock [1–4]. The development of high-throughput methodologies (e.g., Genome-Wide Association Studies, Transcriptomics, Metabolomics, Proteomics, etc.) for the study of the genetic architecture of complex traits allows for the identification of potential candidate genes associated with economically relevant traits in livestock. Taken together, these technologies can substantially improve the accuracy of detection of candidate regions associated with economically important traits across the genome in livestock species [5]. Consequently, the number of QTLs identified across the genome in livestock species increased substantially in the last few years. As of October 2020, the Animal QTLdb can retrieve information about QTLs previously identified in cattle (159,844), chickens (12,508), horses (2,451), pigs (30,871), rainbow trout (584) and sheep (3,411) [6]. The proper integration of results obtained from different methodologies and technologies is a crucial step for the accurate identification of the biological processes regulating complex traits as well as, the identification of potential functional candidate genes for each trait or those shared among traits [5,7–9]. The integration of both structural and functional data can help scrutinize the genetic architecture of economically relevant traits, and consequently, help to better understand complex biological patterns regulating the expression of these traits, such as pleiotropic effects, epistasis, and genetic hitchhiking, among others.

Despite the potential to improve the identification of functional candidate genes and/or QTLs through the integration of multiple data sources, the current process poses limitations in the pipelines and algorithms implemented in the tools available for livestock. Currently, there are

several tools that implement functions for gene (i.e., Biomart and BEDTools) and QTL annotation (Animal QTLdb) [6,10,11]. However, these tools have limitations regarding the automatization process to analyze results from multiple candidate regions (Biomart web application and the R package and Animal QTLdb) or for the visualization of the results. Moreover, although automatization is possible, there is no direct link between the candidate regions and/or markers with the annotated genes and QTLs. Consequently, this gap forces the user to back solve the overlap between the input and output files in order to perform the proper association between the candidate region and/or markers and the annotated genes and/or positional co-localized QTLs. In addition, there is still a need for customized QTL enrichment analyses in the available software and databases. The Genomic Annotation in Livestock for positional candidate LOci (GALLO) is an R package designed to provide an automatized and a straightforward environment for gene and QTL annotation in multiple candidate regions, as well as the integration of data from multiple sources. Additionally, the QTL enrichment analysis can be performed directly by GALLO using the output obtained from the QTL annotation step. GALLO also provides a set of functions for graphical visualization of the annotation, comparison, integration and QTL enrichment results. In this context, the GALLO package was developed as an alternative tool: 1) to allow the integration and simultaneous annotation of multiple datasets for genes and QTLs; 2) to provide graphical visualization tools to visually integrate the annotation and similarity against datasets; 3) to perform QTL enrichment analysis for the positional candidate genomic regions and/or markers associated with economically relevant traits in livestock.

Implementation

The GALLO package was written in the R language [12]. The stable release is available as an R package on CRAN (https://cran.r-project.org/web/packages/GALLO/index.html). The code was

extensively tested with several datasets from different sources and methodologies and reviewed to ensure it meets the packages high quality standards. Additionally, the vignettes were created to be comprehensive and to present practical examples in order to provide a user-friendly tutorial.

The GALLO package provides a useful set of functions that gives a straightforward approach to data integration, comparison, gene and QTL annotation, and visualization of several data sources and methodologies, such as variants from genome-wide association study (GWAS), RNA-Sequencing, whole-genome sequencing, etc. (Figure 1 and Table 1). The main advantage to perform an automated analysis from multiple datasets is the ability to handle the output using different subsets (traits, populations, models, etc.) in the same environment without generating multiple intermediate output files.

Case study – Candidate regions for scrotal circumference and fertility in cattle

The dataset used to present the basic usage and advantages of the GALLO package is composed by the markers significantly associated with scrotal circumference in the Canchim breed [13] and noncompensatory fertility in Holstein cattle [14]. These two studies were previously analyzed together in a systematic review regarding male fertility in cattle [8]. Therefore, the data used herein comprises a multi-study and multi-breed analysis. These candidate markers (527 single nucleotide polymorphisms (SNPs)) are available in Supplementary Table 1. In addition to the candidate markers, we presented as Supplementary Files 1 and 2, the annotation gff file containing the QTL database information for cattle (obtained from the Animal QTLdb; https://www.animalgenome.org/cgi-bin/QTLdb/BT/download?file=gffUMD_3.1) and the gtf file containing the genes annotated in the cattle genome obtained from Ensembl (ftp://ftp.ensembl.org/pub/release-94/gtf/bos_taurus/). The genomic coordinates of both files were

based on the bovine reference genome version UMD 3.1 due to the original coordinates used to report the location of the candidate markers in the original studies. Here, the analysis performed follows the same logical order to the one presented in the GALLO vignette (https://rpubs.com/pablo_bio/GALLO_vignette). However, the dataset used in the user practical tutorial is a subset of the data presented here, aiming to reduce the computational demand for the user. The script with all the commands used to perform the analysis presented here are available in Supplementary File 3. All the tests were performed using a desktop with a processor Intel Core i5 2.4 GHz with 8 Gb of RAM memory.

Importing datasets and annotating genes and QTLs around candidate markers

The first step in the pipeline consists of importing the databases which will be used for the analysis with the *import_gff_gtf()* function. In our specific example, we imported both cattle gene annotation (gtf) and QTL (gff) databases. The *import_gff_gtf()* function receives the database file (db_file) and the file type (*file_type= "gff" or "gtf"*) as arguments and creates a dataframe with the respective information from each file. The system time taken to import the gtf and gff files were 0.045 and 0.311 seconds, respectively, indicating an efficient importing process. The file containing the candidate markers can be imported using any available function in the R environment such as *read.table()* and *read.csv()*.

The main function of GALLO, *find_genes_qtls_around_markers()*, performs the annotation of genes and/or co-localized QTLs within or nearby candidate markers or genomic regions (using the user's defined interval/window). This function uses the information provided in the .gtf file (for gene annotation) or .gff (for QTL annotation) to retrieve the requested information. The output combines the information available in the input file provided by the user with the information

available for the genes and QTLs mapped in the candidate genomic regions. For example, for an input file composed of three genomic coordinates where four genes are annotated in each of the intervals determined by the user, the output file of *find_genes_qtls_around_markers()* will contain 12 rows. The minimum information necessary for the gene and QTL annotation procedures is a data frame with two columns containing the chromosome (CHR) and position in base pairs (BP) in the case of the candidate SNPs input file. In the case of the candidate haplotypes, windows, copy number variations (CNVs) or candidate regions; the input file is composed by three columns corresponding to the chromosome (CHR), the start position in base pairs (BP1) and the end position in base pairs (BP2). Data examples for the candidate markers and windows input files can be obtained using the *data("QTLmarkers")* and *data("QTLwindows")* commands in R. Additionally, examples of QTL and gene annotation results are accessible through the *data("gtfGenes")* and *data("gffQTLs")* commands, respectively. These outputs can be easily handled by summary functions in R, such as *table()*, to obtain information such as the total number of genes and QTLs, the number of genes and QTLs annotated per variants, etc. The gene annotation process was compared with the *getBM()* function from the biomaRt package. The gene annotation process on GALLO needed 0.424 seconds to completely annotate the genes in a 200 Kb interval (upstream and downstream) from candidate markers, while the biomaRt function required 0.019 seconds. The QTL annotation on GALLO was compared with the Bedtools -wao -C command, resulting in 0.851 and 0.12 seconds required for each approach, respectively. It is important to highlight that for both gene and QTL annotation using biomaRt and Bedtools, respectively, a posterior processing of the output file is required in order to match the candidate markers and the genes and QTLs mapped within the candidate intervals. On the other hand, the output file from *find_genes_qtls_around_markers()* function was designed to allow this match in an intuitive way,

combining the rows of both candidate markers file and database files (gff and gtf). Additionally, GALLO allows the user to perform both annotations for genes and QTLs with a single software and programming language. Consequently, GALLO obtains a more elaborate and informative output without substantially compromising the computational demand required for the analysis. The output files obtained in the gene and QTL annotation are available on Supplementary Tables 2 and 3, respectively.

Comparing and visualizing the overlapping of genes and QTLs annotated within the candidate regions

The output file generated by the *find_genes_qtls_around_markers()* function can be used as an input file for the other set of GALLO functions. An advantage from the output of *find_genes_qtls_around_markers()* function is that any additional information present in the input file will be retained in the output file. Consequently, this information can be used to compare the retrieved information between groups of population, methodologies, statistical models, etc. For example, the functions *overlapping_among_groups()* and *plot_overlapping()* can be used to create matrices with the overlapping values among groups and to visualize this overlap. Figure 2 shows the genes and QTLs overlapping between the positional markers obtained in the two selected studies from the dataset of markers analyzed, Feugang et al. (2009) [14] and Buzanskas et al. (2017) [13]. It is important to highlight that the overlapping matrix informing the percentage of shared records is not symmetrical. The percentage of genes from study A shared with the study B, and vice-versa, are calculated as a function of the total number of genes in A or B, respectively. Briefly, this matrix is not symmetrical because GALLO calculates the percentage of records shared as a function of the total number of records for each group. For example, groups A and B shared 5 records, where group A has 10 records in total and group B has 5 records. Consequently, the

percentage of shared records in A is 50% while the percentage of shared genes in B is 100%. In the current example, it is possible to note that only a small percentage of the positional candidate genes were shared between the studies. However, the analyses of overlapping QTLs (using the trait name as reference ID) indicated a higher similarity between the studies, 46% of the QTLs annotated in the candidate regions from Feugang et al. (2010) [14] were also present in Buzanskas et al. (2017) [13] and 93% of the QTLs annotated in the candidate regions from Buzanskas et al. (2017) were also present in Feugang et al. (2010) [13,14].

Understanding the QTL context of the candidate regions

A more precise investigation of the QTL representativeness and diversity can help to better understand the genomic context of the candidate regions. The recurrent association of particular genomic regions with multiple traits might suggest the presence of complex genetic mechanisms regulating that region, such as pleiotropy, epistasis, hitchhiking effect, among others [15,16]. The *plot_qtl_info()* function from GALLO allows for the graphical visualization of the summary of QTL types and traits annotated. The percentage of each QTL type for cattle (i.e., milk, meat and carcass, health, production, reproduction and exterior) annotated within the candidate regions is presented in a pie plot through the use of the argument *qtl_plot=”qtl_type”*, while the percentage of each trait associated with a specific QTL type can be plotted using the argument *qtl_plot=”qtl_name”* and informing the additional argument *qtl_class* (that must receive the name of the QTL class to be plotted). Figure 3 shows that for Feugang et al. (2009) [14] the two most frequent QTL types were Milk (50.42%) and Reproduction (16.97%), while for Buzanskas et al. (2017) [13] the most frequent QTL types were Reproduction (87.06%) and Meat and Carcass (5.03%). An in-depth analyses can be performed for each QTL type in order to observe the frequency of each trait associated with a specific QTL type. The most frequent traits related with

Reproduction QTLs were calving ease (>3%) and scrotal circumference (>60%) for Feugang et al. (2009) and Buzanskas et al. (2017) [13,14], respectively (Figure 3). The comparison between the frequency of traits related with Reproduction QTLs annotated in Feugang et al. (2009) and Buzanskas et al. (2017) [13,14] indicated that among the top 10 most frequent QTLs, calving ease, inhibin levels, stillbirth, interval to first estrus after calving, and birth index were shared between the studies. The combined analysis (not filtering by study) indicated that the Reproduction and Milk QTL types were the two most frequent classes with 76.99% and 10.62% of all QTL types, respectively. In addition, scrotal circumference, inhibin level and calving ease were the most frequent Reproduction QTL related traits in the combined analysis.

QTL enrichment analysis

In some cases, the biases produced with more research in certain areas/traits of higher relevance to animal production (such as milk production related traits in the QTL database for cattle) may result in a larger proportion of records for these traits in the QTL database. Consequently, the simple investigation of the proportion of each QTL type might not be totally useful. The GALLO package allows the user to perform a QTL enrichment analysis to test the significance of the QTL representativeness. The QTL enrichment analysis function in the GALLO package is based on a hypergeometric test approach, where the number of QTLs annotated within the candidate regions for each QTL type or trait, is compared with the observed number of QTLs in the reference database. Briefly, using an enrichment for individual traits in a chromosome-wide approach as an example, the number of traits per chromosome annotated within the candidate regions and the total number of each individual trait in the QTL database are computed. Subsequently, this information is integrated into a hypergeometric test in order to estimate if the number of observed records, for a specific trait, in a chromosome is larger than expected by chance. The *qtl_enrich()* function

allows the user to perform the QTL enrichment analysis for both QTL types and traits (*qtl_type= "QTL_type"* or *"Name"*), for the whole genome or chromosome-wide (*enrich_type= "genome"* or *"chromosome"*) and for all the annotated chromosomes or a subset (*chr.subset= NULL* or the object with the subset of chromosomes). The use of a chromosome-wide enrichment analysis might help to detect specific regions across the genome with a high number of QTLs for a specific trait, i.e. BTA14 in cattle for milk production [17]. A total of 161 unique pairs of traits and chromosomes were tested for the enrichment using the annotated QTLs from both studies. The system time required to perform the enrichment analysis was 5.32 seconds, suggesting efficient processing. The top 10 enriched QTLs (False Discovery Rate (FDR) < 0.05) for the combined analysis is shown in Table 2 and the enrichment results for all the annotated QTLs is shown in Supplementary Table 4. Additionally, GALLO also allows the user to obtain a graphical visualization, in a bubble plot, of the enrichment results using the *QTLenrich_plot()* function. This function receives the enriched table obtained from *qtl_enrich()*, the name of the column with the trait names to be plotted and the name of the column with the p-values to be plotted as arguments. A total of 28 pairs of traits and chromosomes were found to be enriched in the combined analysis, with scrotal circumference (BTA 5, 18, 9, and 21), milk glycosylated kappa-casein percentage (BTA 6 and 16), inhibin level (BTA 5), triglyceride level (BTA 5), milk kappa-casein percentage (BTA 6) and milk iron content (BTA 23) in the list of top 10 most enriched traits. Figure 4 shows the top 5 enriched QTLs identified in this analysis.

Relationship between studies and enriched QTLs

An interesting functionality of GALLO is the graphical visualization of the relationship between groups using a chord plot. The *relationship_plot()* function receives as arguments a dataframe (it can use the gene or QTL annotation results, the QTL enrichment, or any other table with two

groups of information to be compared), the two groups to be compared (arguments x and y) and the graphical arguments to set the size, color and gap between the sector in the chord plot. Figure 5 shows the chord plot obtained using a subset of the QTL annotation dataframe composed only by the top 10 enriched traits and the studies which these traits were annotated. This plot indicates that only inhibin levels and scrotal circumference on BTA5 are shared between Feugang et al. (2009) and Buzanskas et al. (2017) [13,14]. Additionally, milk glycosylated kappa-casein percentage (BTA 6 and 16), milk kappa-casein percentage (BTA 6) and milk iron content (BTA 23) were annotated only in Feugang et al. (2009) [14] and scrotal circumference (BTA 9, 18, 21) and triglyceride level (BTA 5) were annotated only in Buzanskas et al. (2017) [13]. Inhibin is produced by the Sertoli cells and can be used as a biomarker for sexual development [18]. In addition, the inhibin levels were already associated with both scrotal circumference and sperm quality traits in several studies, suggesting an important role in male fertility [19–23]. The results obtained here through the integration of the GWAS results from two independent studies followed by QTL annotation reinforces this association. Additionally, QTLs not associated with reproductive phenotypes were identified in the enrichment analysis, suggesting the presence of complex biological mechanisms such as a pleiotropic effect, epistasis and genetic hitchhiking effect. Previous studies have highlighted the possible role of genomic regions with these kinds of processes in the cattle genome [24,25]. An additional integration of the QTL annotation and enrichment analysis performed here with the gene annotation and prospection for functional candidate genes can be a powerful tool to better understand the genetic architecture and the relationship among complex traits.

Discussion

The GALLO package is composed of a group of functions designed to perform an efficient and direct downstream analysis for the gene and QTL annotation for candidate markers/SNPs, haplotypes, genomic windows, runs of homozygosity, CNVs, etc. The functions implemented in GALLO were designed to allow the integration of multiple datasets simultaneously. A brief summary of these functions is shown in Table 1. For example, GWAS results from multiple traits and/or populations or breeds can be analyzed together and compared or, individually analyzed in the downstream analysis. This can be easily performed by adding an extra column in the input file with the grouping factors to classify each dataset. These input files can be easily adapted from the output of commonly used softwares to analyze high-throughput genomic data, such as PLINK, BLUPF90, DESeq2, etc. [26–28]. In addition, GALLO provides a set of functions designed for the visualization of the annotation results, overlap among groups, relationship between groups (i.e., markers and candidate genes, datasets and QTLs, models and positional candidate genes, etc.), and QTL enrichment results. This set of functions provides the capability of integrating several results from multiple sources including different methodologies (GWAS, RNA-sequencing, proteomics, etc.), populations (breeds, time-points, etc.), traits or the different combination of these groups or others. Taken together, this set of functions provide to the the possibility to perform all the steps of gene/QTL annotation, comparison and summary in the sama environment. Additionally, the output obtained using GALLO was designed to allow a direct connection between the candidate genomic regions and the genes/QTLs which overlap those regions. Therefore, compared with outputs provided by other tools, such as biomaRt and Bedtools, the interpretation of the output provided by GALLO is straightforward and easy to be handle. Finally, the QTL enrichment analysis available on GALLO is a useful and new approach that have

the potential to better understand the relationship between candidate genomic regions and the target phenotype.

A summary of usage examples and output descriptions for all the functions available on GALLO can be found in the reference manual (Supplementary File 4). It is important to highlight that the two studies used as an example here are also part of the bovine QTL database. Consequently, the results obtained here for annotation and enrichment would be expected, once the candidate regions from the example file are present in the database used for the annotation. This approach was used as a proof of concept of the methodology and indicates a precise annotation of the candidate regions.

Conclusion

The integration of multiple datasets for gene and QTL annotation is one of the major bottlenecks for the automatization of functional analysis of the results obtained using high-throughput methodologies. The GALLO package provides a user-friendly and straightforward environment to perform gene and QTL annotation, visualization, data comparison and QTL enrichment for functional studies in livestock species. It is important to highlight that despite the fact that GALLO was primarily designed for livestock species, the package can perform gene annotation and data comparison for any other species without any additional alterations to the input files. Regarding the QTL annotation and the respective graphical visualization, the user should provide the gff file from the QTL database in a format matching the gff files available on Animal QTLdb. Consequently, the use of GALLO in the analyses of data generated from high-throughput methodologies may improve the identification of hidden patterns across datasets, datamining of

previously reported associations, as well as efficiency in the scrutinization of the genetic architecture of complex traits in livestock.

Availability and requirements

Project name: Genomic Annotation in Livestock for positional candidate LOci (GALLO)

Project home page: https://github.com/pablobio/GALLO

Operating system(s): Platform independent

Programming language: R

Other requirements: Depends: R (>= 3.5.0)

License: GPL-3

Availability of supporting data

All of the data analyzed in the present study can be accessed in the public repository hosting the R package (https://github.com/pablobio/GALLO). The input files and results used as examples in the manuscript preparation are available in the supplementary Tables 1-4. A manual including usage examples and output descriptions for all the functions available on GALLO can be found in the package vignette (https://cran.r-project.org/web/packages/GALLO/vignettes/GALLO.html).

Declarations

List of abbreviations

BP: position in base pairs; BP1: start position in base pairs; BP2: end position in base pairs; CHR: Chromosome; CNV: Copy Number Variation; GALLO: Genomic Annotation in Livestock for positional candidate Loci; GWAS: Genome-Wide Association Study; QTL: Quantitative trait loci; SNP: Single Nucleotide Polymorphism.

Ethics approval and consent to participate

Not applicable.

Consent for publication

Not applicable.

Competing interests

The authors declare that they have no competing interests.

Funding

This study was supported by the Sustainable Beef and Forage Science Cluster (FDE.13.17) funded by the Canadian Beef Cattle Check-Off, Beef Cattle Research Council, Alberta Beef Producers, Alberta Cattle Feeders' Association, Beef Farmers of Ontario, La Fédération des Productuers de bovins du Québec, and Agriculture and Agri-Food Canada's Canadian Agricultural Partnership. The funders had no role in study design, data collection and analysis, decision to publish, or preparation of the manuscript.

Authors' contributions

PASF and AC were responsible for the conceptualization. PASF, ASV and AC were responsible for the data processing and review of the codes. PASF and ASV were responsible for data curation. PASF and GM were responsible for the implementation of the bioinformatic pipeline, integration of datasets, and the coding. AC was responsible for funding acquisition.

Acknowledgements

Not applicable.

References

1. Ron M, Weller JI. From QTL to QTN identification in livestock - Winning by points rather than knock-out: A review. Anim. Genet. 2007.

2. Ernst CW, Steibel JP. Molecular advances in QTL discovery and application in pig breeding. Trends Genet. 2013.

3. Miglior F, Fleming A, Malchiodi F, Brito LF, Martin P, Baes CF. A 100-Year Review: Identification and genetic selection of economically important traits in dairy cattle. J Dairy Sci. 2017;

4. Pértille F, Guerrero-Bosagna C, Silva VH Da, Boschiero C, Nunes JDRDS, Ledur MC, et al. High-throughput and Cost-effective Chicken Genotyping Using Next-Generation Sequencing. Sci Rep. 2016;

5. Cánovas A, Reverter A, DeAtley KL, Ashley RL, Colgrave ML, Fortes MRS, et al. Multi-tissue omics analyses reveal molecular regulatory networks for puberty in composite beef cattle. PLoS One. 2014;

6. Hu ZL, Park CA, Reecy JM. Building a livestock genetic and genomic information knowledgebase through integrative developments of Animal QTLdb and CorrDB. Nucleic Acids

Res. 2019;

7. De Souza Fonseca PA, Id-Lahoucine S, Reverter A, Medrano JF, Fortes MS, Casellas J, et al. Combining multi-OMICs information to identify key-regulator genes for pleiotropic effect on fertility and production traits in beef cattle. PLoS One. 2018;13:1–22.

8. Fonseca PA de S, dos Santos FC, Lam S, Suárez-Vega A, Miglior F, Schenkel FS, et al. Genetic mechanisms underlying spermatic and testicular traits within and among cattle breeds: Systematic review and prioritization of GWAS results. J Anim Sci. 2018;

9. Suárez-Vega A, Gutiérrez-Gil B, Benavides J, Perez V, Tosser-Klopp G, Klopp C, et al. Combining GWAS and RNA-Seq approaches for detection of the causal mutation for hereditary junctional epidermolysis bullosa in sheep. PLoS One. 2015;

10. Durinck S, Moreau Y, Kasprzyk A, Davis S, De Moor B, Brazma A, et al. BioMart and Bioconductor: A powerful link between biological databases and microarray data analysis. Bioinformatics. 2005;

11. Quinlan AR, Hall IM. BEDTools: A flexible suite of utilities for comparing genomic features. Bioinformatics. 2010;

12. R Core Team (2019). R: A language and environment for statistical computing. Accessed 1st April 2019. 2019;

13. Buzanskas ME, Grossi D do A, Ventura RV, Schenkel FS, Chud TCS, Stafuzza NB, et al. Candidate genes for male and female reproductive traits in Canchim beef cattle. J Anim Sci Biotechnol. 2017;

14. Feugang JM, Kaya A, Page GP, Chen L, Mehta T, Hirani K, et al. Two-stage genome-wide association study identifies integrin beta 5 as having potential role in bull fertility. BMC Genomics. 2009;

15. Hackinger S, Zeggini E. Statistical methods to detect pleiotropy in human complex traits. Open Biol. 2017.

16. Id-Lahoucine S, Molina A, Cánovas A, Casellas J. Screening for epistatic selection signatures: A simulation study. Sci Rep. 2019;

17. Kühn C, Thaller G, Winter A, Bininda-Emonds ORP, Kaupe B, Erhardt G, et al. Evidence for multiple alleles at the DGAT1 locus better explains a quantitative trait locus with major effect on milk fat content in cattle. Genetics. 2004;

18. Phillips DJ. Activins, inhibins and follistatins in the large domestic species. Domest. Anim. Endocrinol. 2005.

19. Fortes MRS, Reverter A, Kelly M, Mcculloch R, Lehnert SA. Genome-wide association study for inhibin, luteinizing hormone, insulin-like growth factor 1, testicular size and semen traits in bovine species. Andrology. 2013;

20. Fortes MRS, Reverter A, Hawken RJ, Bolormaa S, Lehnert S a. Candidate genes associated with testicular development, sperm quality, and hormone levels of inhibin, luteinizing hormone, and insulin-like growth factor 1 in Brahman bulls. Biol Reprod [Internet]. 2012 [cited 2013 Sep 6];87:58. Available from: http://www.ncbi.nlm.nih.gov/pubmed/22811567

21. Bame JH, Dalton JC, Degelos SD, Good TEM, Ireland JLH, Jimenez-Krassel F, et al. Effect of Long-Term Immunization against Inhibin on Sperm Output in Bulls1. Biol Reprod. 1999;

22. Martin TL, Williams GL, Lunstra DD, Ireland JJ. Immunoneutralization of Inhibin Modifies Hormone Secretion and Sperm Production in Bulls1. Biol Reprod. 1991;

23. Sato T, Kudo T, Ikehara Y, Ogawa H, Hirano T, Kiyohara K, et al. Chondroitin sulfate N-acetylgalactosaminyltransferase 1 is necessary for normal endochondral ossification and aggrecan metabolism. J Biol Chem. 2011;

24. Bolormaa S, Pryce JE, Reverter A, Zhang Y, Barendse W, Kemper K, et al. A Multi-Trait, Meta-analysis for Detecting Pleiotropic Polymorphisms for Stature, Fatness and Reproduction in Beef Cattle. PLoS Genet. 2014;10.

25. Love MI, Huber W, Anders S. Moderated estimation of fold change and dispersion for RNA-seq data with DESeq2. Genome Biol. 2014;15:1–21.

26. Purcell S, Neale B, Todd-Brown K, Thomas L, Ferreira MAR, Bender D, et al. PLINK: A tool set for whole-genome association and population-based linkage analyses. Am J Hum Genet. 2007;

27. Misztal I, Tsuruta S, Strabel T, Auvray B, Druet T, H LD. BLUPF90 and related programs (BGF90). Proc 7th world Congr Genet Appl to Livest Prod. 2002. p. 743–4.

Tables

Table 1: Description of the functions implemented in the GALLO package.

Function	Description	Output
**Gene and QTL annotation**		
import_gff_gtf	Import the gff and gtf files used for QTL and gene annotation, respectively	A dataframe composed by the information present in the gtf and gff files
find_genes_qtls_around_markers	Annotation of genes and QTLs around candidate regions	A data frame composed of the columns present in the input file and the genes or QTLs mapped within or around (if interval provided) the candidate regions
**Data visualization**		
overlapping_among_groups	Overlap between grouping factors (such as different traits, statistical models, populations, studies, stc.)	A list with three matrices: 1) A matrix with the number of overlapping data; 2) A matrix with the percentage of overlap; 3) A matrix with the combination of the two previous ones
plot_overlapping	Plot overlap between data and grouping factors	A heatmap with the overlap between groups
plot_qtl_info	Plot QTL information from the gene or QTL annotation output	A pie plot (if QTL class is chosen) or a bar plot (if trait name is chosen) for the annotated QTLs
relationship_plot	Plot the relationship among the candidate regions or	A chord plot linking a grouping factor (genomic regions, traits, populations, etc.) with the annotated genes or QTLs

	grouping factors with the annotated genes and QTLs	
**QTL enrichment**		
qtl_enrich	Performs a QTL enrichment analysis based on a Bootstrap simulation for each QTL class or trait	A data frame composed of the enrichment results for QTL classes or traits present in the input file. 1) QTL: The QTL class or trait used for the enrichment; 2) CHR: The chromosome for that specific QTL or trait (if the option "chromosome" is informed to the argument enrich_type); 3) N_QTLs: Number of observed QTLs or traits in the dataset; 4) N_QTLs_db: Number of each annotated QTL in the qTL database; 5) Total_annotated_QTLs: Total number of annotated QTLs; 6) Total_QTLs_db: Total number of QTLs in the QTL database; 7) pvalue: P-value for the enrichment analysis; 8) adj.pval: The adjusted p-value based on the multiple test correction selected by the user; 9) QTL_type= The QTL type for each annotated trait.
QTLenrich_plot	Creates a bubble plot with the QTL enrichment results	A plot with the QTL enrichment results

Table 2: Top 10 enriched QTLs for the combined analysis performed with the candidate regions from the two studies, Feugang et al. (2009) and Buzanskas et al. (2017), used in the example dataset.

QTL	CHR	# QTLs	# QTLs db	Total # QTLs	Total # QTLs db	p-value	FDR	QTL type
Scrotal circumference	5	132	134	347	5942	1.56E-171	4.98E-169	Reproduction
Scrotal circumference	18	11	13	41	2147	2.20E-18	3.52E-16	Reproduction
Scrotal circumference	9	11	14	30	1395	2.04E-17	2.18E-15	Reproduction
Milk glycosylated kappa-casein percentage	6	71	1607	204	12158	1.86E-15	1.49E-13	Milk
Inhibin level	5	47	285	347	5942	3.38E-11	2.16E-09	Reproduction
Scrotal circumference	21	4	5	12	3606	3.51E-10	1.87E-08	Reproduction
Milk kappa-casein percentage	6	76	2637	204	12158	2.39E-07	1.01E-05	Milk
Triglyceride level	5	6	7	347	5942	2.53E-07	1.01E-05	Health
Milk glycosylated kappa-casein percentage	16	7	44	21	1440	1.29E-06	4.58E-05	Milk
Milk iron content	23	4	8	19	1159	3.48E-06	0.000111329	Milk

Figure legends:

Figure 1: Workflow explaining the main functions implemented on GALLO. The grey rectangles represent the functions, while the rounded and sharp rectangles represent the main goal of that respective function and its input, respectively.

Figure 2: Overlapping between genes (A) and QTLs (B) annotated within the candidate regions (100 Kb downstream and upstream from the significant markers) from Feugang et al. (2009) and Buzanskas et al. (2017). The darker the color within the squares the higher the percentage of shared genes or QTLs.

Figure 3: Percentage of QTL type (pie plot) and trait related to Reproduction QTLs (barplots) for the QTL annotation results obtained for Feugang et al. (2009) (A), Buzanskas et al. (2017) (B) and the combined analysis (using both studies; C).

Figure 4: Bubble plot displaying the enrichment results for the top 5 enriched QTLs identified using the QTLs annotated within the candidate regions from Feugang et al. (2009) and Buzanskas et al. (2017). The darker the red shade in the circles, the more significant the enrichment. The area of the circles is proportional to the number of QTLs. The x-axis shows a richness factor obtained by the ratio of the number of QTLs annotated in the candidate regions and the total number of each QTL (and chromosome in the case of this plot) in the reference database.

Figure 5: Chord plot showing the relationship between the top 10 enriched QTLs (Scrotal circumference – SCRCIR, Inhibin level – INHIB, Triglyceride level – TRIGLY, Milk glycosylated kappa-casein percentage – MGKCASP, Milk iron content – MFE, Milk kappa-casein percentage - MKCASP) and the studies (Feugang et al. (2009) in purple and Buzanskas et al. (2017) in pink).

Figure 1

Click here to access/download;Figure;Figure1.png

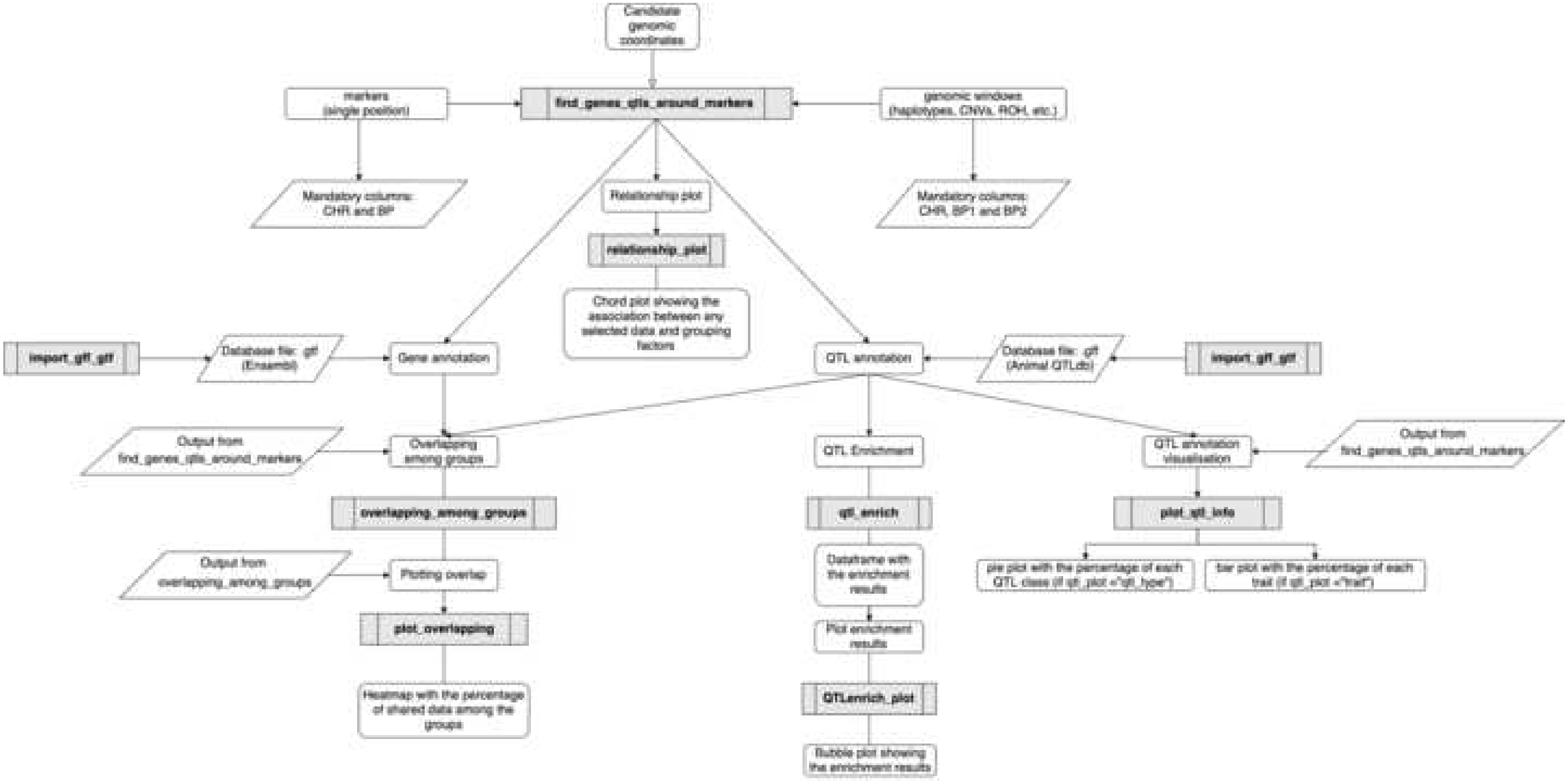

Figure 2

Click here to access/download;Figure;Figure2.png

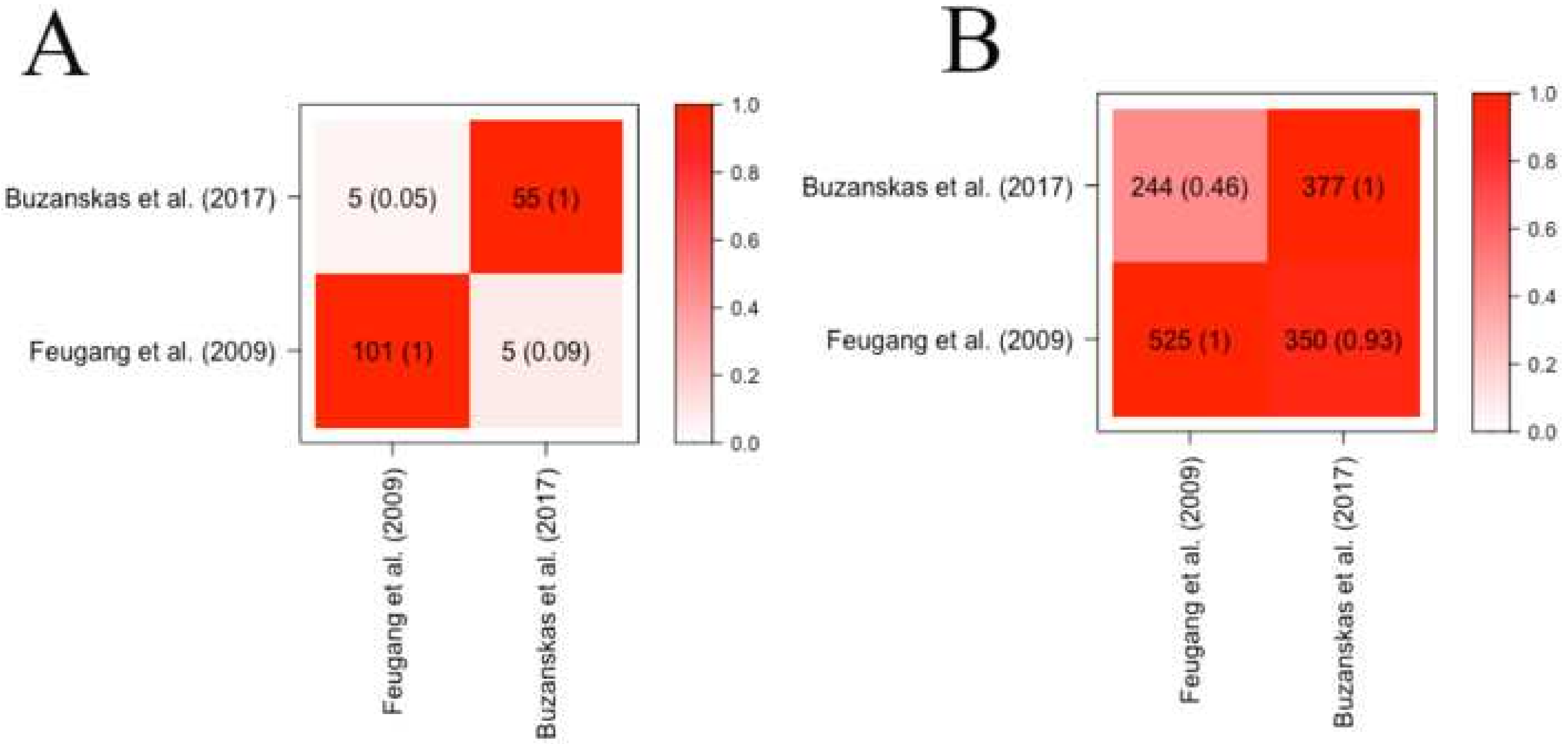

Figure 3

Click here to access/download;Figure;Figure3.png

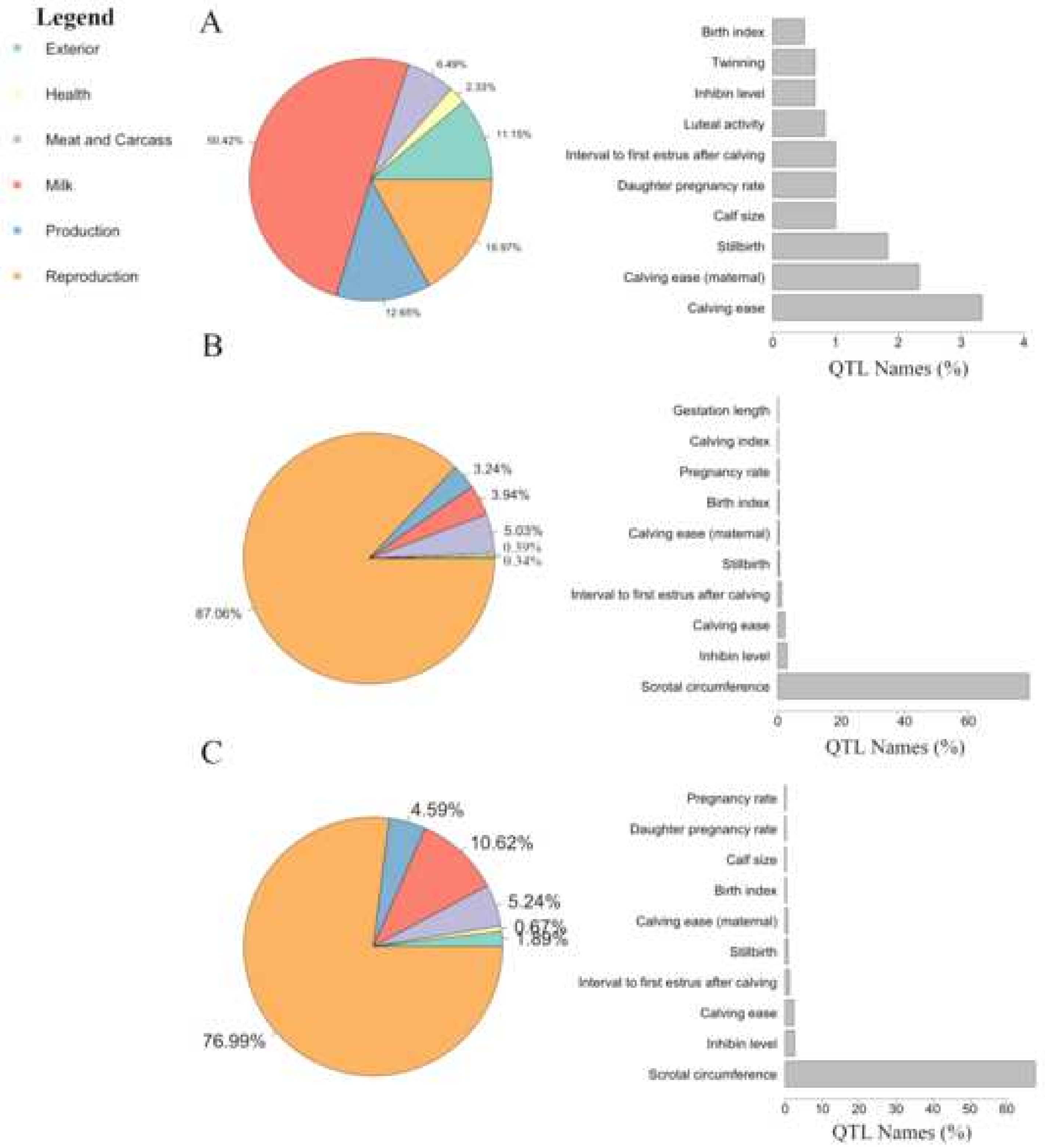

Figure 5

Click here to access/download;Figure;Figure5.png

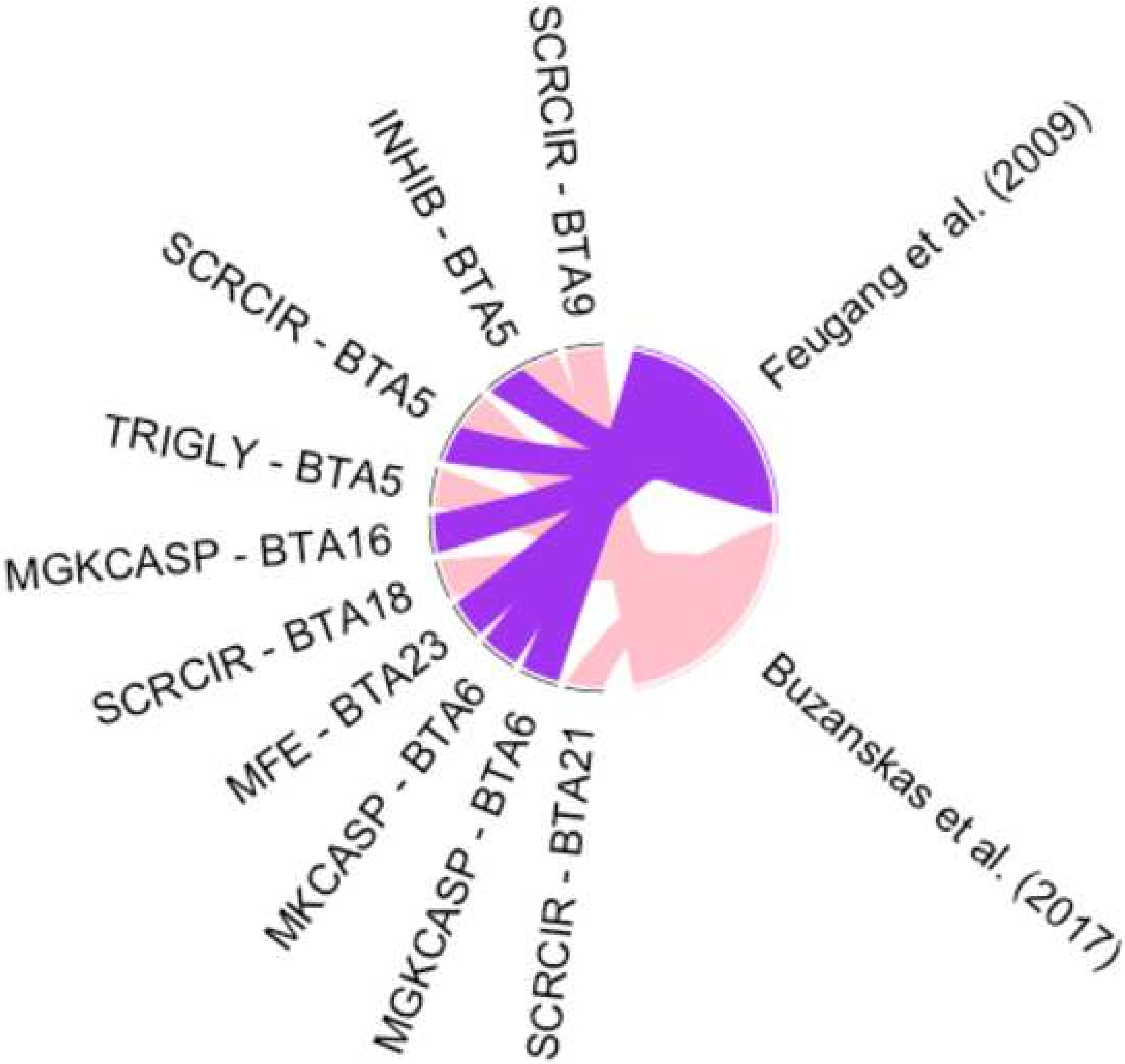

Figure 4

Click here to access/download;Figure;Figure4.png

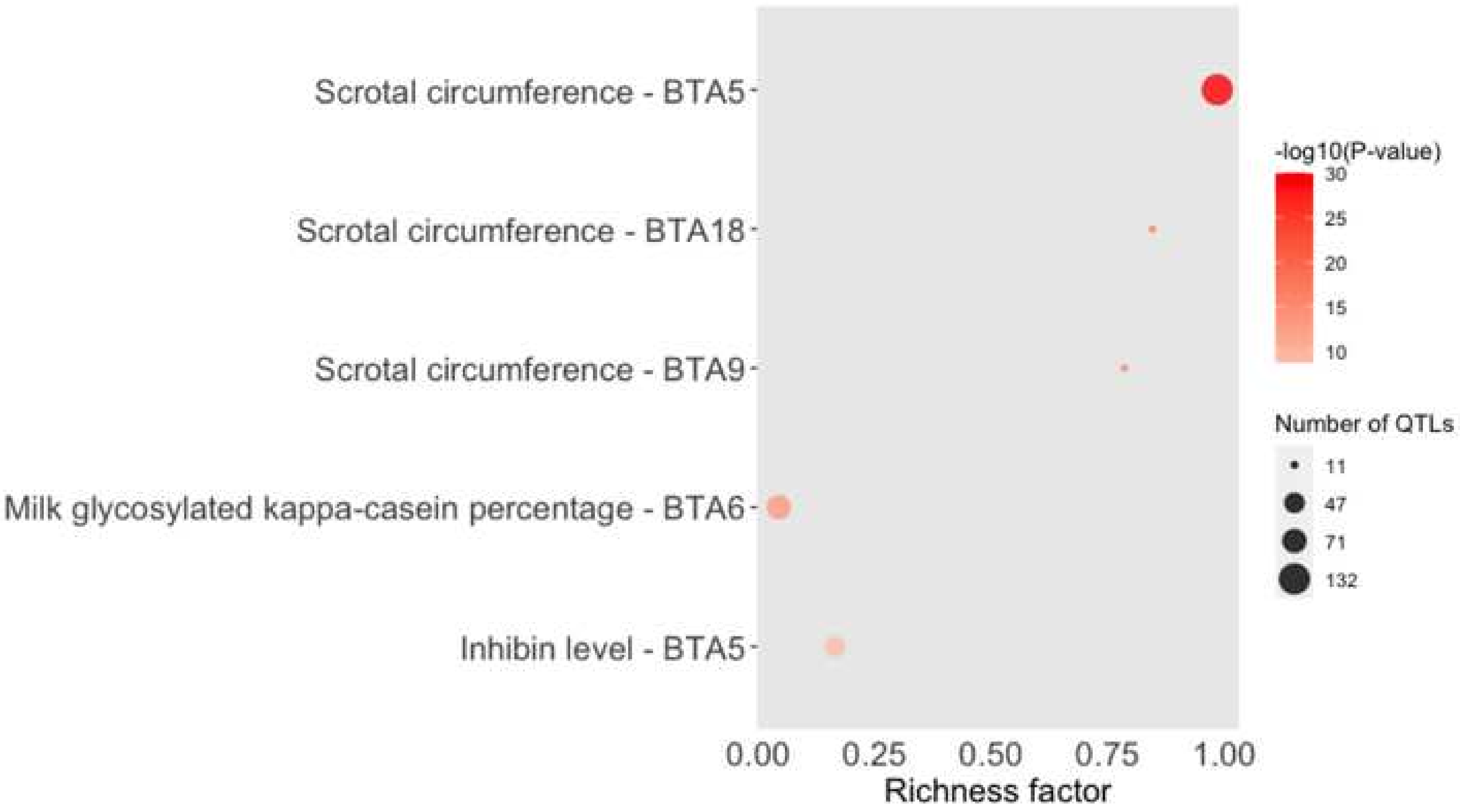

Click here to access/download
Supplementary Material
Supplementary_File1.gtf

Click here to access/download
Supplementary Material
Supplementary_File2.gff.txt

Click here to access/download
Supplementary Material
Supplementary_file3.R

Click here to access/download
Supplementary Material
Supplementary_Table1.txt

Click here to access/download
Supplementary Material
Supplementary_Table2.txt

Click here to access/download
Supplementary Material
Supplementary_Table3.txt

Click here to access/download
Supplementary Material
Supplementary_Table4.txt

Personal Cover
Click here to access/download;Personal Cover;Cover_letter_GALLO_GigaScience_01_09_20_final.doc

Guelph, September 1st, 2020

Dear Editorial Office,

We are pleased to re-submit the manuscript entitled “GALLO: An R package for Genomic Annotation and integration of multiple data source in livestock for positional candidate LOci” for consideration to publish it in the GigaScience. This is a resubmission of this manuscript after the inclusion of all the suggestion and considerations raised by the editor and the prior publication of the package in an official repository, in this case, the CRAN.

The present study introduces the applicability and the functionalities of GALLO package, developed in the R environment.

The identification of quantitative trait loci (QTLs) is a crucial step in the improvement of genomic selection and economic profitability in livestock. The development of high-throughput sequencing and genotyping methodologies and precision livestock farming allowed the identification of thousands of genomic regions associated with several complex traits. Consequently, the number of QTLs identified across the genome in livestock species increased substantially in the last years. Currently, in the Animal QTLdb it is possible to retrieve information about QTLs previously identified in cattle (127,191), chicken (11,340), horse (2,260), pig (29,865), rainbow trout (584) and sheep (3,001). The proper integration of the results obtained from different methodologies and technologies available is a crucial step for the accurate identification of the biological processes regulating the development of complex traits as well as the identification of potential functional candidate genes. However, currently, the integration of multiple data sources is not very straightforward due to limitations in the pipelines and algorithms implemented in the tools available for livestock. Moreover, although the automatization is possible, the direct link between the candidate regions and/or markers with the annotated genes and QTLs is missed. Consequently, this gap is forcing the user to back solve the overlap between the input and output files in order to perform the proper association between the candidate region and/or markers and the annotated genes and/or positional co-localized QTLs. In addition, nowadays there is still a lack of for customized QTL enrichment analyses in the available software and databases. Genomic Annotation in Livestock for positional candidate LOci (GALLO) is an R package, for the accurate annotation of genes and QTLs located in regions identified using the most common genomic analyses performed in livestock, such as Genome-Wide Association Studies and transcriptomics using RNA-Sequencing. Moreover, GALLO allows the graphical visualization of gene and QTL annotation results, data comparison among different grouping factors (e.g., methods, breeds, tissues, statistical models, studies, etc.), and QTL enrichment in different livestock species including cattle, pigs, sheep, chicken, etc. Consequently, GALLO is a useful package for annotation, identification of hidden patterns across datasets, datamining of previous reported associations, as well as the efficient scrutinization of the genetic architecture of complex traits in livestock.

We affirm that this manuscript has not been published elsewhere and is not under consideration by any other journal. All authors have approved the manuscript and agree with its submission to GigaScience.

The authors declare that they have no competing interests. With my best regards,

Angela Cánovas, PhD

Associate Professor Beef Genomics and Small Ruminants
University of Guelph
Department of Animal Biosciences
Centre for Genetics and Improvement of Livestock
Telephone: (519) 824-4129 ext. 56295
email: acanovas@uoguelph.ca
