# Supplementary material for: GALLO: An R package for genomic annotation and integration of multiple data sources in livestock for positional candidate loci
Source: Gigascience. 2020 Dec 30;9(12):giaa149. doi: 10.1093/gigascience/giaa149 (PMC7772745; doi:10.1093/gigascience/giaa149)
Supplement: giaa149_Response_to_Reviewer_Comments_Original_Submission [file giaa149_response_to_reviewer_comments_original_submission.pdf]

Author's Response To Reviewer Comments

Close

Reviewer reports:

Editor comments

Dear Dr. Fonseca,

Your manuscript "GALLO: An R package for Genomic Annotation and integration of multiple data source in livestock for positional candidate LOci" (GIGA-D-20-00265) has been assessed by four reviewers. Based on these reports, I am pleased to inform you that it is potentially acceptable for publication in GigaScience, once you have carried out some essential revisions suggested by our reviewers.

While the overall impression is positive, there are a couple of issues mentioned in the reviewers' reports that require attention during the revision.

I'd like to highlight a few of the points that seem particularly important to me:

- Reviewer 1 points out that a more detailed explanation is required to justify the need for this tool, compared to the available competitors. The reviewer also run into problems when trying to test the tool, this should be fixed.

Answer: In this current version of the manuscript a more detailed explanation regarding the advantages to use GALLO compared with the available tools was provided. Additionally, supplementary file 4 was removed in this version of the manuscript and the examples are available in the package vignette, which was properly cited in the manuscript. In this new version of the vignette the errors were fixed. All the changes in the manuscript are highlighted in this new version.

- Reviewer 3 is a Biometrician and QTL expert, but not working in the livestock field. In principle I agree with his comments that the tool would be of wider interest if it could be applied outside the livestock field. I am aware this may not be your intention, but as the code is open source, you may discuss this point and maybe give some pointers or examples in the manuscript as to how others could build upon this work, to apply the code to problems in other fields.

Answer: Thank you for the comment. We included a discussion about the use of GALLO for other species than livestock. We agree that the package could have a wider interest if the data obtained from other species could be used. This information is available on Lines 298-302.

I agree with reviewer 3 that there should be an easy way to test the code with the data from the paper (e.g. by including a working example with data in github, or you may also consider to provide a computational capsule with code and data in CodeOcean https://codeocean.com/)

Answer: In this current version we provided the proper citation for the package vignette, which contains a series of examples using data that is internally available after the package installation (line 321). Regarding the reviewer's recommendation to use Bioconductor instead of CRAN: From the journal's perspective, this is your decision - R tools presented in GigaScience should be submitted to either CRAN and/or Bioconductor, which platform is selected is the author's choice.

Answer: Thank you for the comment. We addressed the comment from reviewer 3 highlighting the fact that CRAN is the main R repository and all the packages available on CRAN can be used along Bioconductor packages without any problem.

- Reviewer 4 found some technical issues with the R package that I hope you can fix.

Answer: We performed the code edits and fixed the errors listed by the reviewer 4. The package was already updated to CRAN with a new version containing these edits.

Reviewer #1: Fonseca et al. - GALLO: An R package for Genomic Annotation and integration of multiple data source in livestock for potential candidate LOci

Description of useful R package for livestock studies to find overlap between important genomic regions from own results with other studies/public databases and capture it in a visual way, with example based on datasets from 2 GWAS studies on cattle fertility.

Although the paper reads well, some improvement of the English is needed. It is mainly the use of the right tense and plural form, see line-by-line comments below, so please pay attention to that. The sections do not follow a traditional paper setup, which is understandable for the publication of an R package. However the section named Methods also includes Results. Not sure what the journal policy of GigaScience is for paper like this.

Answer: Thank you for the comment. The current version of the manuscript was reviewed by an English native speaker. The sections were restructured in this current version in order to be more clear. All the changes are highlighted in yellow.

The authors indicated that the R package is similar to BiomaRt, and gave performance differences in term of execution time of comparable commands. BiomaRt is a renowned package and was faster. It would be nice if the authors can indicate what benefits GALLO has over BiomaRt. Why was this package needed (e.g. what did you miss in biomaRt)?
Also it may be worthwhile to explicitly indicate why R is the appropriate language for this package. There are thing mentioned scattered over the paper, e.g. like visuals and no need for intermediate output files, please summarize them somewhere.

Answer: Thank you for the comment. The comparison between GALLO and other available tools is better discussed on lines 241-253 and 468-476 of the revised version of the manuscript.

The authors indicated that the matrices showing QTL overlaps were not symmetrical. An explanation for that should be given. Also why many QTLs were overlapping, but only 5 genes. Explaining this will help a user understand what the package does in the background.

Answer: The explanation about the not symmetrical nature of the percentage matrix obtained in GALLO is better explained on lines 167-172. Briefly, this matrix is not symmetrical because GALLO calculates the percentage of records shared as a function of the total number of records for each group. For example, groups A and B shared 5 records, where group
A has 10 records in total and group B has 5 records. Consequently, the percentage of shared records in A is 50% while the percentage of shared genes in B is 100%. Additionally, we provided a better explanation about the QTL annotation. The number of QTLs annotated in a genomic window tend to be substantially larger than the number of genes. This is due to the number of records present. While there are ~20K genes annotated in the bovine genome, the Animal QTLdb has ~160K QTL records spread across the genome. Additionally, a QTL for the same trait, example milk yield, could be annotated in the same loci, but with slightly different windows for different breeds of the same species. This means that although the underlying QTL could be the same, there are different mutations acting in a similar way in the same gene, therefore the record will be different in the QTL database.

I tried to run the code in Supplementary file 4, but was not successful. I struggled loading the gtf and gff files correctly. Below you can find the error I ran into. I guess the file was not loaded as a gtf/gff file, but just as a table. I later tried the published vignette, and there it worked fine following the code provided to load gtf/gff files.

After downloading the gtf file from ensemble following the link and unzipping it, the following command did not work.

```
> out.genes<-find_genes_qtls_around_markers(db_file="Bos_taurus.UMD3.1.94.gtf",
+ marker_file=QTLmarkers, method = "gene",
+ marker = "snp", interval = 500000, nThreads = NULL)
You are using the method: gene with snp
Error in { : task 1 failed - "$ operator is invalid for atomic vectors"
```

The downloaded file looked like this:
head -n6 Bos_taurus.UMD3.1.94.gtf
#!genome-build UMD3.1
#!genome-version UMD3.1
#!genome-date 2009-11
#!genome-build-accession NCBI:GCA_000003055.3
#!genebuild-last-updated 2011-09
1 ensembl gene 19774 19899 . - . gene_id "ENSBTAG00000046619"; gene_version "1"; gene_name "RF00001"; gene_source "ensembl"; gene_biotype "rRNA";

Answer: Thank you very much for your comment. This issue was caused due to an outdated version of supplementary file 4. The submission of the package to CRAN required some changes in the code structure. Mainly regarding the gff and gtf importing process. The code was updated in the revised version of the manuscript. In order to avoid future problems, the supplementary file 4 was removed from the current version of the manuscript and the link for the updated version of GALLO vignette was provided.

Line-by-line comments:
Title Change 'source' to 'sources', and write 'livestock' with capital for the acronym GALLO

Answer: Done.

L15-16 Why precision livestock farming? I associate that with phenotyping using sensors. Remove?

Answer: Thank you for your comment. We decided to remove the term precision livestock farming from the current version of the manuscript.

L38-40 Although the statement about PLF is fine, I find it not so relevant for this manuscript and even a bit distracting

Answer: The sentence was removed in this current version of the manuscript.
L44 Remove 'new' (its relative)
Answer: Done.

L51 Remove 'the development of'

Answer: Done.

L82 Change 'wrote' into 'written'

Answer: Done.

L86-87 Please rephrase the ending of this sentence. Not proper English.

Answer: Done.

L90-91 Is it really the RNA-sequence data & whole genome sequence data (i.e. reads) that can be integrated or is it the called (structural)variants? As I understand from figure one, it is not reads that are supplied, but rather variants. So make sure to be explicit about this.

Answer: Done.

L113 Change 'present' into 'presented'

Answer: Done.

L153 Change 'order' into 'other

Answer: Done.

L166 Change 'can be used compare' into 'can be used to compare'

Answer: Done.

L169 Change second 'overlapping' into 'overlap'

Answer: Done.

L170 Change 'gene' into 'genes'

Answer: Done.

L172 How come the matrices are not symmetrical with respect to number over overlapping QTL? Are there multiple regions from one study overlapping with only one region in the other? I assume the matrix is always symmetrical for overlapping genes?

Answer: Briefly, this matrix is not symmetrical because GALLO calculates the percentage of records shared as a function of the total number of records for each group. For example, groups A and B shared 5 records, where group A has 10 records in total and group B has 5 records. Consequently, the percentage of shared records in A is 50% while the percentage of shared genes in B is 100%. Therefore, in both the gene and QTL data, the percentage matrix can be not symmetrical. A more detailed explanation was presented in the previous comment.

L180-183 Were the genes identified based on the QTL positions? If that is the case, it seems that 5 genes overlapping is rather low with so many QTL overlaps. It would be good to explain what is the reason. I can imagine that QTL in intergenic regions are present, or that QTL regions have only short overlaps not including the genes.

Answer: The genes were identified based on the genomic coordinates of the candidate markers associated with the phenotypes evaluated by Buzanskas et al. (2017) and Feugang et al. (2009). Regarding the number of QTLs and genes annotated in the same genomic regions, the number of QTLs annotated in a genomic window tend to be substantially larger than the number of genes. This is due to the number of records present. While there are ~20K genes annotated in the bovine genome, the Animal QTLdb has ~160K QTL records spread across the genome.

L182-183 I don't understand what you mean here. There are no overlapping genes so why would there be related biological processes?

Answer: Thank you for the comment. This sentence was removed in the current version of the manuscript.

L190 Please define what is meant with QTL types

Answer: The QTL types available for cattle were defined in this current version of the manuscript.

L239 Change 'can used the gene' into 'can be used for the gene'

Answer: Done.

L241 Change 'or' into 'to'

Answer: Done.

L255 Complex what?

Answer: Thank you for the comment. In this current version of the manuscript we included the sentence “complex biological mechanisms”.

L279 Change 'find' into 'found'

Answer: Done.

L281-282 Please rephrase this sentence, not proper English

Answer: Done.

L307 Change 'find' into 'found'

Answer: Done.

L405-407 Reference 27 is a duplicate of reference 10, please correct

Answer: Done.

L435 Change 'overlapping' into 'overlap'

Answer: Done.

L444 The darker red the more significant, not?

Answer: Done.

Figure 4 P-value scale looks like -log10(p-value)

Answer: Thank you for the comment. Indeed, it is -log10(p-value) scale. This was corrected in the current version of the manuscript.

Reviewer #2: I think the GALLO package is useful to scientists specialized in overall genome analyses. Although some of the functions in GALLO can be found in other softwares such as bedtools, the idea of QTL enrichment analysis is highly useful. It is also good to combine all of these tools into one package to further help researchers in conducting the required tasks.
Two issues I would like to raise to further improve the package:

1- I do recommend to include a function that allow for gene enrichment analysis that complement the qtl enrichment analysis.

Answer: Thank you for your suggestion. We are open to the inclusion of new useful functions for GALLO. In this specific case, the development of a gene enrichment analysis is not a simple task as there are fundamental limitations regarding the number of observations for the gene. Using a hypergeometric test as an example (which is the test used for QTL enrichment analysis in GALLO), the number of traits annotated within the candidate regions is compared with the total number of the trait of interest in the QTL database (genome-wide or chromosome-wide, depending of the user choice). In the case of genes, the total number of a gene in the database (the gtf file) will not always be one. On the other hand, the use of functions for the enrichment of gene families, gene ontology terms, and metabolic pathways associated with the positional candidate genes is very useful. However, there are several tools currently available which provide a very accurate and complete toolset of functions for this kind of enrichment. Therefore, we strongly recommend the users to integrate the results obtained on GALLO with other packages in R which can perform this kind of enrichment.

2- Further explanation is required for the hypergeometric test approach to further understand how the QTL enrichment analysis is performed.

Answer: Thank you for your comment. We provided more information regarding the hypergeometric test in this current version of the manuscript (Lines 213-217).

Reviewer #3: This article discusses a newly developed R package GALLO that allows users to quickly annotate quantitative trait loci (QTLs) or genes obtained from genome wide association studies of livestock traits. The package focusses on providing a simple method for linking QTLs/genes to candidate regions in downloadable livestock genomic databases. The package also provides functionality for post-processing of the results through graphical representations and QTL enrichment analyses.

Its clear this package does fill a need for users working specifically in genome wide association research

of livestock traits. However, I have outlined some issues associated with the article/package and its possible alignment with the journal aims and scope.

This is quite a simple package. Its main task is matching and returning overlapping content between two data frames in R where one of the data frame has a potentially large number of rows associated with it. In my opinion, this innate package simplicity reduces the strength of the article/package and its alignment with publication in GigaScience.

Answer: The functions available on GALLO comprise a much more diverse group of tasks than just the simple matching and overlapping between data frames. As stated in the manuscript:

“Currently, there are several tools that implement functions for gene (i.e., Biomart and BEDTools) and QTL annotation (Animal QTLdb). However, these tools have limitations regarding the automatization process to analyze results from multiple candidate regions (Biomart web application and the R package and Animal QTLdb) or for the visualization of the results. Moreover, although the automatization is possible, the direct link between the candidate regions and/or markers with the annotated genes and QTLs is missed. Consequently, this gap is forcing the user to back solve the overlap between the input and output files in order to perform the proper association between the candidate region and/or markers and the annotated genes and/or positional co-localized QTLs.”

In addition to the advantages provided by the annotation function of GALLO mentioned above, GALLO provides the user a set of functions for graphical visualization and comparison of the results obtained by multiple studies, statistical models, populations, etc. It is important to highlight that currently there is no software, package, or function available for QTL enrichment using the information available in the Animal QTLdb, the most complete and reliable database for QTLs identified in livestock species. GALLO is the first package to provide this function and allow the user to perform the enrichment using a genome-wide and chromosome-wide approach, in addition to a QTL type or trait selection. This kind of function is extremely useful due to the bias of investigation of several traits in livestock species, such as milk production traits. Additionally, the option for chromosome-wide analysis helps to adjust for the effects of specialized regions in the genome, such as chromosome 29 for meat quality traits in cattle and chromosome 14 for lipid content in milk (and milk production in general).

Taken together, these functionalities of GALLO are a unique set of tools for data integration, annotation and comparison in association studies with a strong emphasis on livestock species.

The functionality has been written specifically for livestock genetics. Why can't this be more general and provide functionality for a other related biological organisms such as heavily researched crops like wheat, maize or barley? I understand this may not be the authors intent but the narrow scope of the package lessens its potential for publication in a quality journal such as GigaScience.

Answer: The functions available on GALLO can be used for any other species. The main reason we reinforce the livestock application is the use of the Animal QTLdb information for QTL annotation. Once the user uses a similar format for QTL annotation for any other species, the functions of GALLO will behave exactly the same as the livestock species available on Animal QTLdb. We acknowledge this comment in the revised version of the manuscript and have included a sentence highlighting the applicability to other species (Lines 298-302).

From a visibility perspective it feels like it would be more natural for this package to be in the Bioconductor repository so it could potentially link with overarching gene annotation packages such as AnnotationData.

Answer: The package is currently accepted and available on CRAN, which is the main repository for R packages. Despite the specialization of Bioconductor for packages related with “biological analysis” CRAN also has a high visibility and deposited packages can be easily linked with packages available on other repositories, such as Bioconductor.

The software package is a very recent submission to CRAN. From past experience, the publication of packaged code that has been recently created can be problematic. Immature code has the potential to require many more dramatic amendments, additions and bug fixes.

Answer: The package is already accepted and published on CRAN. All edits to the code suggested by automatic and manual checking were already provided and accepted by the CRAN team. As any

package, GALLO is under constant code evaluation and updating. For the moment, any major bug has been reported. However, as soon as these problems are identified they will be fixed, and the package will be updated on CRAN. The package was already used for several research groups which resulted in several manuscripts currently published, accepted or under development. Some examples are shown below:

Lam, S., et al. Development and comparison of RNA-Sequencing pipelines for more accurate SNP identification: Practical example of functional SNP detection associated with feed efficiency in Nellore beef cattle. BMC Genomics 21: 703 (2020). https://doi.org/10.1186/s12864-020-07107-7

Lam. S., et al. Identification of functional candidate variants (SNPs and INDELs) and genes for feed efficiency in Holstein and Jersey cattle breeds using RNA-Sequencing. Journal of Dairy Science. 2020. In press.

Sweett, H., et al. Genome-wide association study to identify genomic regions and positional candidate genes associated with male fertility in beef cattle. Accepted for publication in Scientific Reports.

I would have liked the ability to immediately test the code with the data sets that are mentioned in the Method section of the paper. However, the submitted R script does not contain code that matches the code mentioned in the manuscript. In fact, the script contains path names from the authors local computer.

Answer: The R script submitted as supplementary material was edited in order to provide a more detailed step by step analysis of the code and data provided. It is important to highlight that the package also has a vignette which comprises a different dataset with a complete explanation of each function and output.

The title of the paper has been expanded from the title of the R package. Im not sure there is good justification for this and I am immediately concerned about the spelling error in the title for the article. It should be the plural ``sources''.

Answer: The manuscript is an introduction to the R package. Therefore, we choose to include the complete name of the package in order to provide an easier way for the users to identify the manuscript associated with the package. Regarding the typo on the title, the error was fixed in the revised version of the manuscript.

Following from this previous point, although the paper is quite well written, it needs a pre-submission editor with english as their first language to proofread the main document text. This would create a more succinct manuscript through removal of repeated content and more general punctuation issues.

Answer: Thank you for the comment. The current version of the manuscript was reviewed by an English native speaker.

Reviewer #4: Overall the manuscript is well written, easy and logical to follow and also presents an interesting addition to the toolbox of genomic data analysis with R. Despite the fact, that the manuscript makes an overall good impression to me, I have a few comments that I would like the authors to address. In detail these are
Specific R-package comments

1. Please check the styling of the code chunks in the manual (e.g. spacing, linebreaks, etc.)

Answer: Thank you for the comment. We reviewed all the code styles for both manual and vignette present on GALLO. The package is currently accepted and updated on CRAN as well.

2. import_gff_gtf(): I think the function could estimate the filetype from the filename (strsplit -> ifelse) so that this parameter could be optional.

Answer: Thank you for your suggestion. We decided to let the user inform the file extension due to potential problems with the names of the gtf and gff files when downloaded from the respective databases. For example, the gff files from Animal QTLdb are constantly renamed as ".gff.txt" after the decompress process.

3. find_genes_qtls_around_markers(): Please add also a match.arg for the `marker` input

Answer: Thank you for the suggestion. The match.arg was included in the find_genes_qtls_around_markers() function.

4. Instead of referring to the table() command in line 142 (actually, I am not sure how to get the number of genes with it), I would recommend to create S3 classes for important return objects and then create own summary(), print() and possibly even plot() functions for it.

Answer: Thank you for your comment. Assuming the gene or qtl annotation results were saved in a data.frame called out.results, the number of genes and QTLs can be easily retrieved with the following commands, table(out.results$gene_name) and table(out.results$traits), respectively. New functions for plots and summary statistics are currently under development for GALLO and will be available in the next update.

5. QTLenrich_plot(): In the vignette, the scale for the p-value goes up to 100. If you use the label 'P-value', please keep it between 0 and 1, or change the label name. Also, I am not sure about the colors, in the example of the vignette, the 'P-value' with 100 is red, whereas smaller p-values are white (in contrast to what is written in the Figure3 caption). So, currently the description and the labels do not match. Further, although white coloured bubbles are less informative and maybe this is a problem with my screen, but from the figure I hardly could see any bubbles (besides the red ones...), maybe you could slightly adjust the colours or the background?

Answer: Thank you for your comment. The label was changed in the revised version of the manuscript and vignette. The correct scale is -log10(p-value). The description of the figure was corrected as well. Regarding the background of the plot, thank you very much for the suggestion. The plot will be updated in order to provide a light grey background, which will make the small white dots easier to see.

How do you handle the situation, when a large dark bubble is covering a smaller (dark) bubble, would the user see that or would that be hidden? Maybe using a frame and then plotting from large to small could solve this?

Answer: Thank you for your comment. The QTLenrich_plot() function allows the user to freely decide the order of plots for the enrichment results. Therefore, if an overlap is observed between two or more records, the user can rearrange the order of the plots and avoid this problem.

6. Something is odd with your parallel code. When I run the code below, the runtime is getting longer with more cores I use:

```
> system.time(out.genes<-find_genes_qtls_around_markers(db_file=gtfGenes,
+ marker_file=QTLmarkers[rep(1:141,500),], method = "gene",
+ marker = "snp", interval = 500000, nThreads = 2))
You are using the method: gene with snp
user system elapsed
0.81 0.28 5.45

> system.time(out.genes<-find_genes_qtls_around_markers(db_file=gtfGenes,
+ marker_file=QTLmarkers[rep(1:141,500),], method = "gene",
+ marker = "snp", interval = 500000, nThreads = 4))
You are using the method: gene with snp
user system elapsed
0.87 0.32 6.30

> system.time(out.genes<-find_genes_qtls_around_markers(db_file=gtfGenes,
+ marker_file=QTLmarkers[rep(1:141,500),], method = "gene",
+ marker = "snp", interval = 500000, nThreads = NULL))
You are using the method: gene with snp
user system elapsed
0.87 0.24 1.77
```

The same is true for all other functions I tried that have a nThread option. Whenever I choose NULL, it is faster than 2 or 4...

Further, I would prefer that the parallel functions accept nThreads=1 as valid input.

Answer: Thank you for your comment. The issue regarding the parallel code seems to be solved in the current version of the package, which is accepted and updated on CRAN. Additionally, we edited the code to allow nThreads=1 as a valid input. In Figure 1 of this review, we show a boxplot representing the distribution of the elapsed time for the qtl annotation using 3 options of nThreads: 2, 4, and NULL after 100 iterations. It is important to highlight that the NULL option result in the use of all available cores in the machine.

Figure 1: Violin plot showing the distribution of elapsed time (seconds) for three options of nThreads argument of find_genes_qtls_around_markers() function after 100 iterations. In red, green and blue, two, four and all available cores were chosen, respectively.

7. plot_qtl_info() really easily creates an error that the figure margins are too large. Please catch this better. Also, I think you require many graphical parameters from the user to enter, what makes the use of the plotting functions kind of cumbersome. I think you could add functions that estimate the best fitting values for the user as default. Especially that the user needs to change the par() settings shouldn't happen often.

Answer: Thank you for your comment. The issue with the margins seems to be caused by the position of the legend in the pie plot. We introduced a new argument allowing the user to define the legend position (horizontal or vertical). Regarding the number of arguments, the majority of the graphical arguments can work with the default options, as well as any other plot. However, due to the complexity of the plot schemes and the number of available records, additional arguments were necessary in order to provide a better visualization scheme for the user.

8. In the vignette 0.3.3.2 it should say dev.off() instead of dev.off

Answer: Done.

9. In QTLenrich_plot() there are smaller bubbles than mentioned in the legend. Please add also the small ones to the legend

Answer: Done.

10. There are still few notes and warnings in the cran check, that probably easily can be resolved. I think that should be done.

Answer: All notes and warnings were related to minor issues such as the size of the data folder and the new submission email and ID of the maintainer. These issues are fixed.

Minor comments:

l.1: I suppose 'livestock' should be capitalized also in the title to get the abbreviation GALLO?

Answer: Done.

l.47: Please add an date when you checked those numbers from animal QTLdb, when I checked they appear larger

Answer: Thank you for the comment. It is fixed in the current version of the manuscript

l.70: The 'functional' you do not have in other descriptions of the name, maybe it would be nice to be consistent

Answer: Done.

l.139: (and others): Please format code snippets consistent (data(...)) e.g. with monospace or italic, as you did. Further, I would prefer to use quotions rather than variable names in the data calls (like

data("QTLwindow"))

Answer: Thank you for the comment. We applied the same format for all the code snippets across the manuscript.

l.145: Though hardly noticable by the user, I wouldn't say that the performances are similar between the compared tools. Biomart seems to be faster by factor 22 and BEDtools by factor 7. Maybe you could rephrase it?

Answer: Thank you for your comment. We removed the sentence where the similarity between the efficiency of the software was compared. Additionally, on lines 151-152 we included the following sentence: "Consequently, GALLO obtains a more elaborate and informative output without substantially compromising the computational demand of the analysis".

General comments:
1. Maybe it is a matter of taste or formatting guidelines, but I would prefer seeing code snippets written in a monospace rather then using italics.

Answer: Thank you for your comment. We think that the italic is a good way to highlight the codes in the manuscript. Additionally, we are following the writing style of previous R packages publications available on GigaScience.

2. Please check that code snippets are consistent formatted throughout the manuscript

Answer: Done.

Close
