# Supplementary material for: GALLO: An R package for genomic annotation and integration of multiple data sources in livestock for positional candidate loci
Source: Gigascience. 2020 Dec 30;9(12):giaa149. doi: 10.1093/gigascience/giaa149 (PMC7772745; doi:10.1093/gigascience/giaa149)
Supplement: giaa149_Response_to_Reviewer_Comments_Revision_1 [file giaa149_response_to_reviewer_comments_revision_1.pdf]

Author's Response To Reviewer Comments

Close

Reviewer reports:
Reviewer #1: The authors have clarified the questions I had and included the clarification appropriately in the manuscript.

L298-302: this should be in the discussion not in the conclusion. One reviewer suggested to include more species than just livestock and the authors responded that it is possible as long as the species has a resource list like the AnimalQTL database. "We acknowledge this comment in the revised version of the manuscript and have included a sentence highlighting the applicability to other species (Lines 298-302)." This sentence is added to the conclusion, however, I urge discussing it in the discussion. It is a point of discussion and not a conclusion of the core manuscript. (If properly discussed it may be included in the conclusion.)

Answer: Thank you for your suggestion. We edited the current version of the manuscript and the information about applicability to other species was moved to the discussion section.

Some more textual errors arose in the newly written sections:
L277: remove one 'the'

Answer: Done.

L278: change 'sama' into 'same'

Answer: Done.

L282: change 'easy to be handle' into easy to handle of easy to be handled

Answer: Done.

L283: change 'have' into 'has'

Answer: Done.

Just a note for future revisions: For this comment & answer below (Reviewer 1) I didn't find the sections on the lines indicated, but elsewhere (141-153 & 277-285). Please make sure you refer to the correct line-numbers in the future to accommodate the reviewers.

The authors indicated that the R package is similar to BiomaRt, and gave performance differences in term of execution time of comparable commands. BiomaRt is a renowned package and was faster. It would be nice if the authors can indicate what benefits GALLO has over BiomaRt. Why was this package needed (e.g. what did you miss in biomaRt)?
Also it may be worthwhile to explicitly indicate why R is the appropriate language for this package. There are thing mentioned scattered over the paper, e.g. like visuals and no need for intermediate output files, please summarize them somewhere.

Answer: Thank you for the comment. The comparison between GALLO and other available tools is better discussed on lines 241-253 and 468-476 of the revised version of the manuscript.

Answer: Thank you very much for the comment. In the next submissions the authors will be awarded

about this issue.

Close
