# Supplementary material for: GALLO: An R package for genomic annotation and integration of multiple data sources in livestock for positional candidate loci
Source: Gigascience. 2020 Dec 30;9(12):giaa149. doi: 10.1093/gigascience/giaa149 (PMC7772745; doi:10.1093/gigascience/giaa149)
Supplement: giaa149_Reviewer_1_Report_Original_Submission — Aniek Bouwman -- 9/20/2020 Reviewed [file giaa149_reviewer_1_report_original_submission.pdf]

Reviewer Report

Title: GALLO: An R package for Genomic Annotation and integration of multiple data source in livestock for positional candidate LOci

Version: Original Submission **Date:** 9/20/2020

Reviewer name: Aniek Bouwman

Reviewer Comments to Author:

Fonseca et al. - GALLO: An R package for Genomic Annotation and integration of multiple data source in livestock for potential candidate LOci

Description of useful R package for livestock studies to find overlap between important genomic regions from own results with other studies/public databases and capture it in a visual way, with example based on datasets from 2 GWAS studies on cattle fertility.

Although the paper reads well, some improvement of the English is needed. It is mainly the use of the right tense and plural form, see line-by-line comments below, so please pay attention to that. The sections do not follow a traditional paper setup, which is understandable for the publication of an R package. However the section named Methods also includes Results. Not sure what the journal policy of GigaScience is for paper like this.

The authors indicated that the R package is similar to BiomaRt, and gave performance differences in term of execution time of comparable commands. BiomaRt is a renowned package and was faster. It would be nice if the authors can indicate what benefits GALLO has over BiomaRt. Why was this package needed (e.g. what did you miss in biomaRt)?

Also it may be worthwhile to explicitly indicate why R is the appropriate language for this package. There are thing mentioned scattered over the paper, e.g. like visuals and no need for intermediate output files, please summarize them somewhere.

The authors indicated that the matrices showing QTL overlaps were not symmetrical. An explanation for that should be given. Also why many QTLs were overlapping, but only 5 genes. Explaining this will help a user understand what the package does in the background.

I tried to run the code in Supplementary file 4, but was not successful. I struggled loading the gtf and gff files correctly. Below you can find the error I ran into. I guess the file was not loaded as a gtf/gff file, but just as a table. I later tried the published vignette, and there it worked fine following the code provided to load gtf/gff files.

After downloading the gtf file from ensemble following the link and unzipping it, the following command did not work.

```
> out.genes<-find_genes_qtls_around_markers(db_file="Bos_taurus.UMD3.1.94.gtf",
+ marker_file=QTLmarkers, method = "gene",
+ marker = "snp", interval = 500000, nThreads = NULL)
You are using the method: gene with snp
Error in { : task 1 failed - "$ operator is invalid for atomic vectors"
```

The downloaded file looked like this:

head -n6 Bos_taurus.UMD3.1.94.gtf
#!genome-build UMD3.1
#!genome-version UMD3.1
#!genome-date 2009-11
#!genome-build-accession NCBI:GCA_000003055.3
#!genebuild-last-updated 2011-09
1 ensembl gene 19774 19899 . - . gene_id "ENSBTAG00000046619"; gene_version "1"; gene_name "RF00001"; gene_source "ensembl"; gene_biotype "rRNA";

Line-by-line comments:

Title Change 'source' to 'sources', and write 'livestock' with capital for the acronym GALLO

L15-16 Why precision livestock farming? I associate that with phenotyping using sensors. Remove?

L38-40 Although the statement about PLF is fine, I find it not so relevant for this manuscript and even a bit distracting

L44 Remove 'new' (its relative)

L51 Remove 'the development of'

L82 Change 'wrote' into 'written'

L86-87 Please rephrase the ending of this sentence. Not proper English.

L90-91 Is it really the RNA-sequence data & whole genome sequence data (i.e. reads) that can be integrated or is it the called (structural)variants? As I understand from figure one, it is not reads that are supplied, but rather variants. So make sure to be explicit about this.

L113 Change 'present' into 'presented'

L153 Change 'order' into 'other

L166 Change 'can be used compare' into 'can be used to compare'

L169 Change second 'overlapping' into 'overlap'

L170 Change 'gene' into 'genes'

L172 How come the matrices are not symmetrical with respect to number over overlapping QTL? Are there multiple regions from one study overlapping with only one region in the other? I assume the matrix is always symmetrical for overlapping genes?

L180-183 Were the genes identified based on the QTL positions? If that is the case, it seems that 5 genes overlapping is rather low with so many QTL overlaps. It would be good to explain what is the reason. I can imagine that QTL in intergenic regions are present, or that QTL regions have only short overlaps not including the genes.

L182-183 I don't understand what you mean here. There are no overlapping genes so why would there be related biological processes?

L190 Please define what is meant with QTL types

L239 Change 'can used the gene' into 'can be used for the gene'

L241 Change 'or' into 'to'

L255 Complex what?

L279 Change 'find' into 'found'

L281-282 Please rephrase this sentence, not proper English

L307 Change 'find' into 'found'

L405-407 Reference 27 is a duplicate of reference 10, please correct

L435 Change 'overlapping' into 'overlap'
L444 The darker red the more significant, not?
Figure 4 P-value scale looks like -log10(p-value)

Level of Interest

Please indicate how interesting you found the manuscript: Choose an item.

Quality of Written English

Please indicate the quality of language in the manuscript: Choose an item.

Declaration of Competing Interests

Please complete a declaration of competing interests, considering the following questions:

- Have you in the past five years received reimbursements, fees, funding, or salary from an organisation that may in any way gain or lose financially from the publication of this manuscript, either now or in the future?
- Do you hold any stocks or shares in an organisation that may in any way gain or lose financially from the publication of this manuscript, either now or in the future?
- Do you hold or are you currently applying for any patents relating to the content of the manuscript?
- Have you received reimbursements, fees, funding, or salary from an organization that holds or has applied for patents relating to the content of the manuscript?
- Do you have any other financial competing interests?
- Do you have any non-financial competing interests in relation to this paper?

If you can answer no to all of the above, write 'I declare that I have no competing interests' below. If your reply is yes to any, please give details below.

I declare that I have no competing interests

I agree to the open peer review policy of the journal. I understand that my name will be included on my report to the authors and, if the manuscript is accepted for publication, my named report including any attachments I upload will be posted on the website along with the authors' responses. I agree for my report to be made available under an Open Access Creative Commons CC-BY license (http://creativecommons.org/licenses/by/4.0/). I understand that any comments which I do not wish to be included in my named report can be included as confidential comments to the editors, which will not be published.

Choose an item.

To further support our reviewers, we have joined with Publons, where you can gain additional credit to further highlight your hard work (see: https://publons.com/journal/530/gigascience). On publication of this paper, your review will be automatically added to Publons, you can then choose whether or not to claim your Publons credit. I understand this statement.

Yes Choose an item.
