# Supplementary material for: GALLO: An R package for genomic annotation and integration of multiple data sources in livestock for positional candidate loci
Source: Gigascience. 2020 Dec 30;9(12):giaa149. doi: 10.1093/gigascience/giaa149 (PMC7772745; doi:10.1093/gigascience/giaa149)
Supplement: giaa149_Reviewer_1_Report_Revision_1 — Aniek Bouwman -- 11/10/2020 Reviewed [file giaa149_reviewer_1_report_revision_1.pdf]

Reviewer Report

Title: GALLO: An R package for Genomic Annotation and integration of multiple data source in livestock for positional candidate LOci

Version: Revision 1 **Date:** 11/10/2020

Reviewer name: Aniek Bouwman

Reviewer Comments to Author:

The authors have clarified the questions I had and included the clarification appropriately in the manuscript.

L298-302: this should be in the discussion not in the conclusion. One reviewer suggested to include more species than just livestock and the authors responded that it is possible as long as the species has a resource list like the AnimalQTL database. "We acknowledge this comment in the revised version of the manuscript and have included a sentence highlighting the applicability to other species (Lines 298-302)." This sentence is added to the conclusion, however, I urge discussing it in the discussion. It is a point of discussion and not a conclusion of the core manuscript. (If properly discussed it may be included in the conclusion.)

Some more textual errors arose in the newly written sections:

L277: remove one 'the'

L278: change 'sama' into 'same'

L282: change 'easy to be handle' into easy to handle of easy to be handled

L283: change 'have' into 'has'

Just a note for future revisions: For this comment & answer below (Reviewer 1) I didn't find the sections on the lines indicated, but elsewhere (141-153 & 277-285). Please make sure you refer to the correct line-numbers in the future to accommodate the reviewers.

The authors indicated that the R package is similar to BiomaRt, and gave performance differences in term of execution time of comparable commands. BiomaRt is a renowned package and was faster. It would be nice if the authors can indicate what benefits GALLO has over BiomaRt. Why was this package needed (e.g. what did you miss in biomaRt)?

Also it may be worthwhile to explicitly indicate why R is the appropriate language for this package. There are thing mentioned scattered over the paper, e.g. like visuals and no need for intermediate output files, please summarize them somewhere.

Answer: Thank you for the comment. The comparison between GALLO and other available tools is better discussed on lines 241-253 and 468-476 of the revised version of the manuscript.

Level of Interest

Please indicate how interesting you found the manuscript: Choose an item.

Quality of Written English

Please indicate the quality of language in the manuscript: Choose an item.

Declaration of Competing Interests

Please complete a declaration of competing interests, considering the following questions:

- Have you in the past five years received reimbursements, fees, funding, or salary from an organisation that may in any way gain or lose financially from the publication of this manuscript, either now or in the future?
- Do you hold any stocks or shares in an organisation that may in any way gain or lose financially from the publication of this manuscript, either now or in the future?
- Do you hold or are you currently applying for any patents relating to the content of the manuscript?
- Have you received reimbursements, fees, funding, or salary from an organization that holds or has applied for patents relating to the content of the manuscript?
- Do you have any other financial competing interests?
- Do you have any non-financial competing interests in relation to this paper?

If you can answer no to all of the above, write 'I declare that I have no competing interests' below. If your reply is yes to any, please give details below.

I declare that I have no competing interests

I agree to the open peer review policy of the journal. I understand that my name will be included on my report to the authors and, if the manuscript is accepted for publication, my named report including any attachments I upload will be posted on the website along with the authors' responses. I agree for my report to be made available under an Open Access Creative Commons CC-BY license (http://creativecommons.org/licenses/by/4.0/). I understand that any comments which I do not wish to be included in my named report can be included as confidential comments to the editors, which will not be published.

Choose an item.

To further support our reviewers, we have joined with Publons, where you can gain additional credit to further highlight your hard work (see: https://publons.com/journal/530/gigascience). On publication of this paper, your review will be automatically added to Publons, you can then choose whether or not to claim your Publons credit. I understand this statement.

Yes Choose an item.
