# Supplementary material for: GALLO: An R package for genomic annotation and integration of multiple data sources in livestock for positional candidate loci
Source: Gigascience. 2020 Dec 30;9(12):giaa149. doi: 10.1093/gigascience/giaa149 (PMC7772745; doi:10.1093/gigascience/giaa149)
Supplement: giaa149_Reviewer_2_Report_Original_Submission — Hussain Bahbahani -- 9/30/2020 Reviewed [file giaa149_reviewer_2_report_original_submission.pdf]

Reviewer Report

Title: GALLO: An R package for Genomic Annotation and integration of multiple data source in livestock for positional candidate LOci

Version: Original Submission **Date:** 9/30/2020

Reviewer name: Hussain Bahbahani

Reviewer Comments to Author:

I think the GALLO package is useful to scientists specialized in overall genome analyses. Although some of the functions in GALLO can be found in other softwares such as bedtools, the idea of QTL enrichment analysis is highly useful. It is also good to combine all of these tools into one package to further help researchers in conducting the required tasks.
Two issues I would like to raise to further improve the package:
1- I do recommend to include a function that allow for gene enrichment analysis that complement the qtl enrichment analysis.
2- Further explanation is required for the hypergeometric test approach to further understand how the QTL enrichment analysis is performed.

Level of Interest

Please indicate how interesting you found the manuscript: Choose an item.

Quality of Written English

Please indicate the quality of language in the manuscript: Choose an item.

Declaration of Competing Interests

Please complete a declaration of competing interests, considering the following questions:

- Have you in the past five years received reimbursements, fees, funding, or salary from an organisation that may in any way gain or lose financially from the publication of this manuscript, either now or in the future?
- Do you hold any stocks or shares in an organisation that may in any way gain or lose financially from the publication of this manuscript, either now or in the future?
- Do you hold or are you currently applying for any patents relating to the content of the manuscript?
- Have you received reimbursements, fees, funding, or salary from an organization that holds or has applied for patents relating to the content of the manuscript?
- Do you have any other financial competing interests?
- Do you have any non-financial competing interests in relation to this paper?

If you can answer no to all of the above, write 'I declare that I have no competing interests' below. If your reply is yes to any, please give details below.

No to all six points

I agree to the open peer review policy of the journal. I understand that my name will be included on my report to the authors and, if the manuscript is accepted for publication, my named report including any attachments I upload will be posted on the website along with the authors' responses. I agree for my report to be made available under an Open Access Creative Commons CC-BY license (http://creativecommons.org/licenses/by/4.0/). I understand that any comments which I do not wish to be included in my named report can be included as confidential comments to the editors, which will not be published.

Choose an item.

To further support our reviewers, we have joined with Publons, where you can gain additional credit to further highlight your hard work (see: https://publons.com/journal/530/gigascience). On publication of this paper, your review will be automatically added to Publons, you can then choose whether or not to claim your Publons credit. I understand this statement.

Yes Choose an item.
