# Supplementary material for: GALLO: An R package for genomic annotation and integration of multiple data sources in livestock for positional candidate loci
Source: Gigascience. 2020 Dec 30;9(12):giaa149. doi: 10.1093/gigascience/giaa149 (PMC7772745; doi:10.1093/gigascience/giaa149)
Supplement: giaa149_Reviewer_3_Report_Original_Submission — Julian Taylor -- 10/5/2020 Reviewed [file giaa149_reviewer_3_report_original_submission.pdf]

Reviewer Report

Title: GALLO: An R package for Genomic Annotation and integration of multiple data source in livestock for positional candidate LOci

Version: Original Submission **Date:** 10/5/2020

Reviewer name: Julian Taylor

Reviewer Comments to Author:

This article discusses a newly developed R package GALLO that allows users to quickly annotate quantitative trait loci (QTLs) or genes obtained from genome wide association studies of livestock traits. The package focusses on providing a simple method for linking QTLs/genes to candidate regions in downloadable livestock genomic databases. The package also provides functionality for post-processing of the results through graphical representations and QTL enrichment analyses.
Its clear this package does fill a need for users working specifically in genome wide association research of livestock traits. However, I have outlined some issues associated with the article/package and its possible alignment with the journal aims and scope.
This is quite a simple package. Its main task is matching and returning overlapping content between two data frames in R where one of the data frame has a potentially large number of rows associated with it. In my opinion, this innate package simplicity reduces the strength of the article/package and its alignment with publication in GigaScience.
The functionality has been written specifically for livestock genetics. Why can't this be more general and provide functionality for a other related biological organisms such as heavily researched crops like wheat, maize or barley? I understand this may not be the authors intent but the narrow scope of the package lessens its potential for publication in a quality journal such as GigaScience.
From a visibility perspective it feels like it would be more natural for this package to be in the Bioconductor repository so it could potentially link with overarching gene annotation packages such as AnnotationData.
The software package is a very recent submission to CRAN. From past experience, the publication of packaged code that has been recently created can be problematic. Immature code has the potential to require many more dramatic amendments, additions and bug fixes.
I would have liked the ability to immediately test the code with the data sets that are mentioned in the Method section of the paper. However, the submitted R script does not contain code that matches the code mentioned in the manuscript. In fact, the script contains path names from the authors local computer.
The title of the paper has been expanded from the title of the R package. Im not sure there is good justification for this and I am immediately concerned about the spelling error in the title for the article. It should be the plural ``sources''.
Following from this previous point, although the paper is quite well written, it needs a pre-submission editor with english as their first language to proofread the main document text. This would create a more succinct manuscript through removal of repeated content and more general punctuation issues.

Level of Interest

Please indicate how interesting you found the manuscript: Choose an item.

Quality of Written English

Please indicate the quality of language in the manuscript: Choose an item.

Declaration of Competing Interests

Please complete a declaration of competing interests, considering the following questions:

- Have you in the past five years received reimbursements, fees, funding, or salary from an organisation that may in any way gain or lose financially from the publication of this manuscript, either now or in the future?
- Do you hold any stocks or shares in an organisation that may in any way gain or lose financially from the publication of this manuscript, either now or in the future?
- Do you hold or are you currently applying for any patents relating to the content of the manuscript?
- Have you received reimbursements, fees, funding, or salary from an organization that holds or has applied for patents relating to the content of the manuscript?
- Do you have any other financial competing interests?
- Do you have any non-financial competing interests in relation to this paper?

If you can answer no to all of the above, write 'I declare that I have no competing interests' below. If your reply is yes to any, please give details below.

I declare that I have no competing interests

I agree to the open peer review policy of the journal. I understand that my name will be included on my report to the authors and, if the manuscript is accepted for publication, my named report including any attachments I upload will be posted on the website along with the authors' responses. I agree for my report to be made available under an Open Access Creative Commons CC-BY license (http://creativecommons.org/licenses/by/4.0/). I understand that any comments which I do not wish to be included in my named report can be included as confidential comments to the editors, which will not be published.

Choose an item.

To further support our reviewers, we have joined with Publons, where you can gain additional credit to further highlight your hard work (see: https://publons.com/journal/530/gigascience). On publication of

this paper, your review will be automatically added to Publons, you can then choose whether or not to claim your Publons credit. I understand this statement.

Yes Choose an item.
