# Supplementary material for: GALLO: An R package for genomic annotation and integration of multiple data sources in livestock for positional candidate loci
Source: Gigascience. 2020 Dec 30;9(12):giaa149. doi: 10.1093/gigascience/giaa149 (PMC7772745; doi:10.1093/gigascience/giaa149)
Supplement: giaa149_Reviewer_4_Report_Original_Submission — Daniel Fischer -- 10/5/2020 Reviewed [file giaa149_reviewer_4_report_original_submission.pdf]

Reviewer Report

Title: GALLO: An R package for Genomic Annotation and integration of multiple data source in livestock for positional candidate LOci

Version: Original Submission **Date:** 10/6/2020

Reviewer name: Daniel Fischer

Reviewer Comments to Author:

Overall the manuscript is well written, easy and logical to follow and also presents an interesting addition to the
toolbox of genomic data analysis with R. Despite the fact, that the manuscript makes an overall good impression
to me, I have a few comments that I would like the authors to address. In detail these are
Specific R-package comments
1. Please check the styling of the code chunks in the manual (e.g. spacing, linebreaks, etc.)
2. import_gff_gtf(): I think the function could estimate the filetype from the filename (strsplit -> ifelse)
so that this parameter could be optional.
3. find_genes_qtls_around_markers(): Please add also a match.arg for the `marker` input
4. Instead of referring to the table() command in line 142 (actually, I am not sure how to get the number of
genes with it), I would recommend to create S3 classes for important return objects and then create own
summary(), print() and possibly even plot() functions for it.
5. QTLenrich_plot(): In the vignette, the scale for the p-value goes up to 100. If you use the label 'P-value', please
keep it between 0 and 1, or change the label name. Also, I am not sure about the colors, in the example of the
vignette, the 'P-value' with 100 is red, whereas smaller p-values are white (in contrast to what is written
in the Figure3 caption). So, currently the description and the labels do not match.
Further, although white coloured bubbles are less informative and maybe this is a problem with my screen, but
from the figure I hardly could see any bubbles (besides the red ones...), maybe you could slightly adjust the
colours or the background?
How do you handle the situation, when a large dark bubble is covering a smaller (dark) bubble, would the user see
that or would that be hidden? Maybe using a frame and then plotting from large to small could solve this?
6. Something is odd with your parallel code. When I run the code below, the runtime is getting longer

with more cores I use:

```
> system.time(out.genes<-find_genes_qtls_around_markers(db_file=gtfGenes,
+                                 marker_file=QTLmarkers[rep(1:141,500),], method = "gene",
+                                 marker = "snp", interval = 500000, nThreads = 2))
You are using the method: gene with snp
  user  system elapsed
  0.81    0.28    5.45
> system.time(out.genes<-find_genes_qtls_around_markers(db_file=gtfGenes,
+                                 marker_file=QTLmarkers[rep(1:141,500),], method = "gene",
+                                 marker = "snp", interval = 500000, nThreads = 4))
You are using the method: gene with snp
  user  system elapsed
  0.87    0.32    6.30
> system.time(out.genes<-find_genes_qtls_around_markers(db_file=gtfGenes,
+                                 marker_file=QTLmarkers[rep(1:141,500),], method = "gene",
+                                 marker = "snp", interval = 500000, nThreads = NULL))
You are using the method: gene with snp
  user  system elapsed
  0.87    0.24    1.77
```

The same is true for all other functions I tried that have a nThread option. Whenever I choose NULL, it is faster than 2 or 4...

Further, I would prefer that the parallel functions accept nThreads=1 as valid input.

7. plot_qtl_info() really easily creates an error that the figure margins are too large. Please catch this better. Also, I think you require many graphical parameters from the user to enter, what makes the use of the plotting functions kind of cumbersome. I think you could add functions that estimate the best fitting values for the user as default. Especially that the user needs to change the par() settings shouldn't happen often.
8. In the vignette 0.3.3.2 it should say dev.off() instead of dev.off
9. In QTLenrich_plot() there are smaller bubbles than mentioned in the legend. Please add also the small ones to the legend
10. There are still few notes and warnings in the cran check, that probably easily can be resolved. I think that should be done.

Minor comments:

l.1: I suppose 'livestock' should be capitalized also in the title to get the abbreviation GALLO?

l.47: Please add an date when you checked those numbers from animal QTLdb, when I checked they appear larger

l.70: The 'functional' you do not have in other descriptions of the name, maybe it would be nice to be consistent

l.139: (and others): Please format code snippets consistent (data(...)) e.g. with monospace or italic, as you did.

Further, I would prefer to use quotions rather than variable names in the data calls (like data("QTLwindow"))
l.145: Though hardly noticable by the user, I wouldn't say that the performances are similar between the compared tools. Biomart seems to be faster by factor 22 and BEDtools by factor 7. Maybe you could rephrase it?
General comments:
1. Maybe it is a matter of taste or formatting guidelines, but I would prefer seeing code snippets written in a monospace rather then using italics.
2. Please check that code snippets are consistent formatted throughout the manuscript

Level of Interest

Please indicate how interesting you found the manuscript: Choose an item.

Quality of Written English

Please indicate the quality of language in the manuscript: Choose an item.

Declaration of Competing Interests

Please complete a declaration of competing interests, considering the following questions:

- Have you in the past five years received reimbursements, fees, funding, or salary from an organisation that may in any way gain or lose financially from the publication of this manuscript, either now or in the future?
- Do you hold any stocks or shares in an organisation that may in any way gain or lose financially from the publication of this manuscript, either now or in the future?
- Do you hold or are you currently applying for any patents relating to the content of the manuscript?
- Have you received reimbursements, fees, funding, or salary from an organization that holds or has applied for patents relating to the content of the manuscript?
- Do you have any other financial competing interests?
- Do you have any non-financial competing interests in relation to this paper?

If you can answer no to all of the above, write 'I declare that I have no competing interests' below. If your reply is yes to any, please give details below.

'I declare that I have no competing interests

I agree to the open peer review policy of the journal. I understand that my name will be included on my report to the authors and, if the manuscript is accepted for publication, my named report including any attachments I upload will be posted on the website along with the authors' responses. I agree for my report to be made available under an Open Access Creative Commons CC-BY license (http://creativecommons.org/licenses/by/4.0/). I understand that any comments which I do not wish to be included in my named report can be included as confidential comments to the editors, which will not be published.

Choose an item.

To further support our reviewers, we have joined with Publons, where you can gain additional credit to further highlight your hard work (see: https://publons.com/journal/530/gigascience). On publication of this paper, your review will be automatically added to Publons, you can then choose whether or not to claim your Publons credit. I understand this statement.

Yes Choose an item.
